# Origins of the selectivity of late transition metals of Group 9 and Group 10 for oxidative addition of C–H *vs.* C–Cl bonds

**DOI:** 10.1039/d6sc00090h

**Published:** 2026-04-08

**Authors:** Yehao Qiu, Alistair J. Sterling, Kevin K. Ikeda, Alexander Zech, Matthias Loipersberger, Diptarka Hait, Martin Head-Gordon, John F. Hartwig

**Affiliations:** a Department of Chemistry, University of California Berkeley California 94720 USA mhg@cchem.berkeley.edu jhartwig@berkeley.edu; b Department of Chemistry and Biochemistry, The University of Texas at Dallas Richardson Texas 75080 USA

## Abstract

Oxidative addition of carbon–hydrogen or carbon–halogen bonds occurs in many reactions catalyzed by late transition-metal complexes. Metals of Group 9 typically react with C–H bonds faster than C–X bonds, while those of Group 10 react with C–X bonds faster than C–H bonds. We conducted comparative computational studies on the oxidative addition of C–H and C–Cl bonds in chlorobenzene to complexes of Rh, Ir, Pd, and Pt. Our calculations show that addition of an aryl C–Cl bond to complexes of Rh(i) or Ir(i) is thermodynamically more favorable but kinetically less favorable than addition of an aryl C–H bond. Oxidative addition of a C–Cl bond to Pd(0) or Pt(0) complexes is also thermodynamically favorable but addition of an aryl C–H bond to Pd(0) or Pt(0) is less favorable and endergonic. Energy decomposition analysis and natural population analysis of the reactions with Ir(i) and Pd(0) suggest that interactions in the transition state between the electrophilic Ir(i) and the more electron-rich C–H bond are more stabilizing than those between Ir(i) and the more electrophilic C–Cl bond and account for the higher reactivity of C–H bonds, whereas the weakness of the Pd–H bond causes addition of the C–H bond to Pd(0) to be endergonic and less reactive than the C–Cl bond; additional factors, such as Pd(0) and Pt(0) nucleophilicity, also contribute to these selectivities.

## Abstract 2

The selectivity of Pd, Pt, Rh, and Ir complexes for the oxidative additions of carbon–hydrogen *vs.* carbon–halogen bonds is controlled by differences in thermodynamics, electronic properties, and degrees of distortions in the transition states.

## Graphical abstract

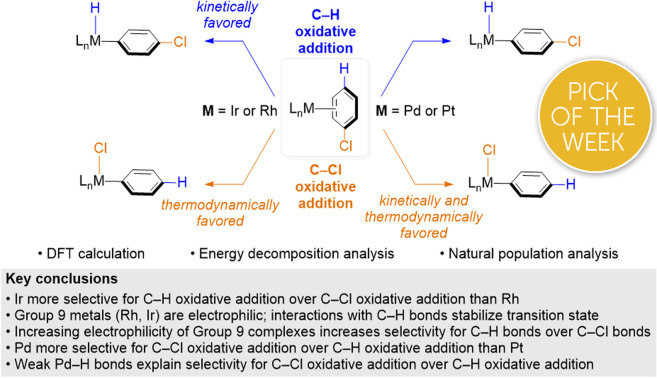

## 1 Introduction

The oxidative addition of a single bond (Y–Z) to a transition-metal center (M) typically forms a new M–Y and a new M–Z bond by cleavage of the Y–Z bond. The formal oxidation state of the metal center is increased by +2 in this process.^1^ The oxidative additions of carbon–hydrogen (C–H) and carbon–halogen (C–X) bonds to transition-metal complexes are key steps in many important catalytic reactions, such as the functionalization of C–H bonds^2–7^ and cross-coupling reactions.^8–14^ Iridium complexes are well-known to activate or functionalize C–H bonds, but they rarely undergo oxidative addition of carbon–halogen bonds.^4,15–18^ In contrast, palladium complexes readily undergo oxidative additions of carbon–halogen or carbon–pseudohalogen bonds and catalyze a large variety of cross-coupling reactions, but they rarely undergo oxidative addition of C–H bonds.^8–14^ Although many Pd-catalyzed functionalizations of C–H bonds have been reported, Pd complexes cleave C–H bonds, in almost all cases, by electrophilic activation or a concerted-metalation-deprotonation (CMD) mechanism.^19–26^ During a CMD process, the formal oxidation state of the Pd center does not change. Studies on the origins of the high selectivity of Pd complexes to oxidatively add C–X bonds over C–H bonds and the high selectivity of Ir complexes to oxidatively add C–H bonds over C–X bonds would answer fundamental questions about the reactivity of complexes of these transition metals, which catalyze a wide range of reactions, and will help establish foundational principles for the future development of catalytic reactions.

Computational methods, including density functional theory (DFT) and energy decomposition analysis (EDA), have been a powerful tool for understanding the property, reactivity and selectivity of transition-metal complexes.^27–35^ However, most of such studies focused on individual types of transition-metal complexes or catalysts^36–44^ and comparative studies of the reactivity and selectivity of complexes containing transition metals from different groups and rows are rare. For example, energy barriers were calculated for the oxidative addition of aryl C–H and C–Cl bonds to cationic and neutral iridium complexes, but detailed explanation for the selectivity of these complexes and comparisons with transition metals from different groups and rows were not provided.^36,37^ We envision that a comparative analysis of the oxidative addition of C–H and C–X bonds to Ir and Pd complexes based on thermodynamic and kinetic data, partial charges, and EDA will reveal the key factors that determine the difference in selectivity of these complexes for oxidative additions of C–H *versus* C–X bonds and, thus, will facilitate the design of transition metal catalysts.

**Scheme 1:**
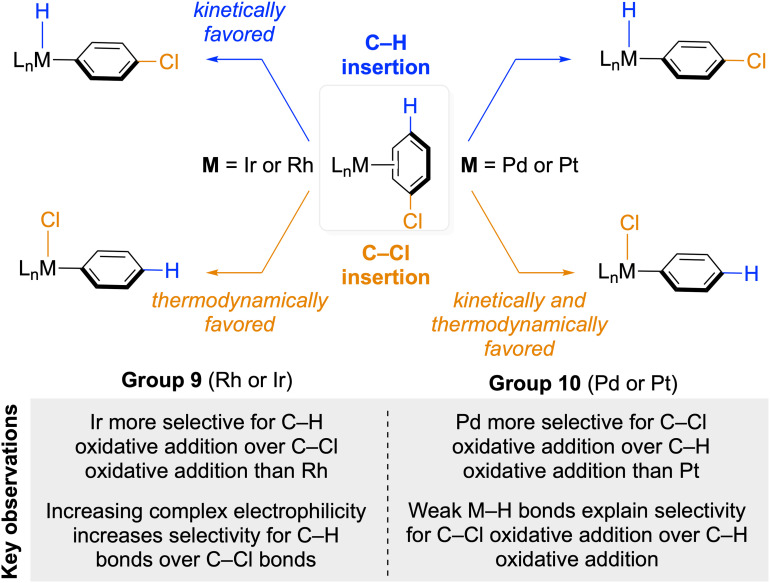
Oxidative addition of the *para*-C–H bond and the C–Cl bond in chlorobenzene to Group 9 and Group 10 transition metal complexes and key observations from this work and previous experimental studies.

Here, we report computational investigations of the origin of the high selectivity of Pd complexes for oxidative addition of C–Cl bonds over C–H bonds and the origin of the high selectivity of Ir complexes for oxidative addition of C–H bonds over C–Cl bonds. Density functional theory calculations and energy decomposition analysis were performed on the (L1)iridium complexes (L1 = 2,6-bis((dimethylphosphino)methyl)pyridine) and (L2)palladium complexes (L2 = (1-adamantyl)-di-*tert*-butylphosphine) undergoing oxidative addition of the C–H or the C–Cl bond in chlorobenzene (PhCl). To investigate the effect of second-row *versus* third-row transition metals on oxidative addition and the effect of the overall charge of the complex, we also computed oxidative additions of the same C–H and C–Cl bonds to (L1)rhodium complexes, (L2)platinum complexes, and a neutral iridium(i) complex containing an LXL-type ligand L3 (L3 = bis(2-(dimethylphosphino)phenyl)amide). These computations suggest that oxidative addition of an aryl C–H bond by the (L2)Pd(0) complex is endergonic, whereas oxidative addition of an aryl C–Cl bond to (L2)Pd(0) is exergonic. Oxidative addition of a C–H bond to the (L1)Ir(i) complex is less exergonic than that of a C–Cl bond, but the barrier to oxidative addition of a C–H bond to Ir is lower than that of a C–Cl bond. The selectivity of the cationic (L1)Ir complex for the oxidative addition of an aryl C–H bond over an aryl C–Cl bond is greater than that of the neutral (L3)Ir complex. Oxidative additions of both the C–H and the C–Cl bond to third-row metals is more exergonic (or less endergonic) than that to second-row metals, and the barriers to oxidative addition to second-row metals are higher than those to third-row metals. Energy decomposition analysis and natural population analysis (NPA) reveal that transfer of electron density from the breaking bond to Ir occurs before transfer of electron density from Ir to the breaking bond during oxidative addition of an aryl C–H or C–Cl bond to iridium complexes, and that a more electron-donating ligand on Ir leads to an earlier transition state.^45^ The cationic (L1)iridium(i) complex is highly selective for oxidative addition of C–H bonds over C–Cl bonds because the stabilization afforded by charge transfer between the d orbitals on Ir and the σ and σ* orbitals of the C–H bond in the transition state is greater than that between Ir and the C–Cl bond in the analogous transition state. In contrast, the high selectivity of (L2)palladium(0) complexes for the oxidative addition of C–Cl bonds over C–H bonds is primarily caused by the endergonicity of the oxidative addition of C–H bonds and the weakness of the Pd–H bond (Scheme 1).

## 2 Results and discussions

### 2.1 Selection of model systems for oxidative additions of C–H and C–Cl bonds

We chose to study oxidative additions of the C–Cl and C–H bonds in chlorobenzene (PhCl) to the cationic Ir complex of the tridentate PNP pincer ligand L1 (L1 = 2,6-bis((dimethylphosphino)methyl)pyridine), which contains an L-type nitrogen donor, and oxidative addition of the same arene to the Pd complex containing the trialkylphosphine ligand L2 (L2 = (1-adamantyl)-di-*tert*-butylphosphine) because activation of aryl C–H bonds by Ir(i) complexes containing ligands similar to L1 and oxidative addition of aryl halides to (L2)Pd(0) complexes are well-documented.^36,46–48^ Iridium complexes of 2,6-bis((di-*tert*-butylphosphino)methyl)pyridine (tBuL1), which is a bulky analog of ligand L1, were reported to undergo selective oxidative addition of aryl C–H bonds over aryl C–Cl bonds.^46,48^ Results from calculations on (tBuL1)Ir^+^ complexes are consistent with reported experimental results, suggesting that our choice of ligand L1 is suitable for this study (see SI). We chose the bulky ligand L2 because the monoligated species (L2)Pd(0) was reported to undergo oxidative addition of aryl halides^47^ and complications caused by competing oxidative addition to Pd(0) species containing different numbers of phosphine ligands would be avoided. We also computed oxidative addition to the Ir complex of a PNP pincer ligand containing an X-type nitrogen donor L3 (L3 = bis(2-(dimethylphosphino)phenyl)amide) to assess the effect of the charge of the complex on the thermodynamics of and barriers to oxidative addition of the C–Cl and C–H bonds and computed oxidative addition of the same C–H or C–Cl bond in PhCl to (L1)Rh(i) and (L2)Pt(0) complexes to assess the effect of the row of the transition metals (2nd *vs.* 3rd) on the oxidative addition. Computations on this set of complexes also revealed the effect of the group (Group 9 *vs.* Group 10 metals) on the oxidative addition by enabling a comparison between the reactivity of (L1)Rh and of (L2)Pd and the reactivity of (L1)Ir and of (L2)Pt.

### 2.2 Geometries and energies of the reactants, transition states, and products

**Fig. 1:**
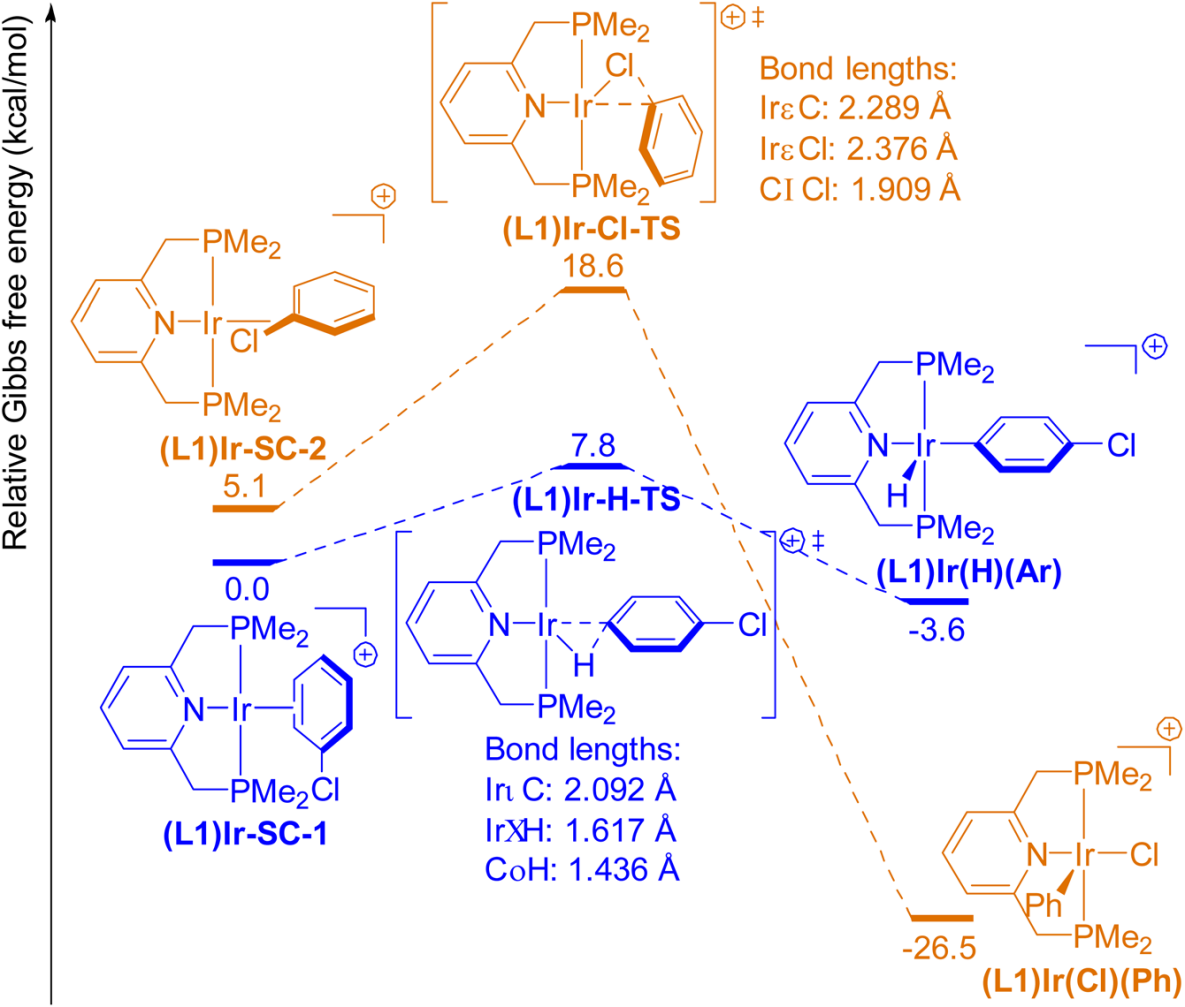
Free-energy diagram for the oxidative addition of the *para*-C–H bond (blue pathway) and the C–Cl bond (orange pathway) in PhCl to a cationic (L1)Ir(i) complex.

**Fig. 2:**
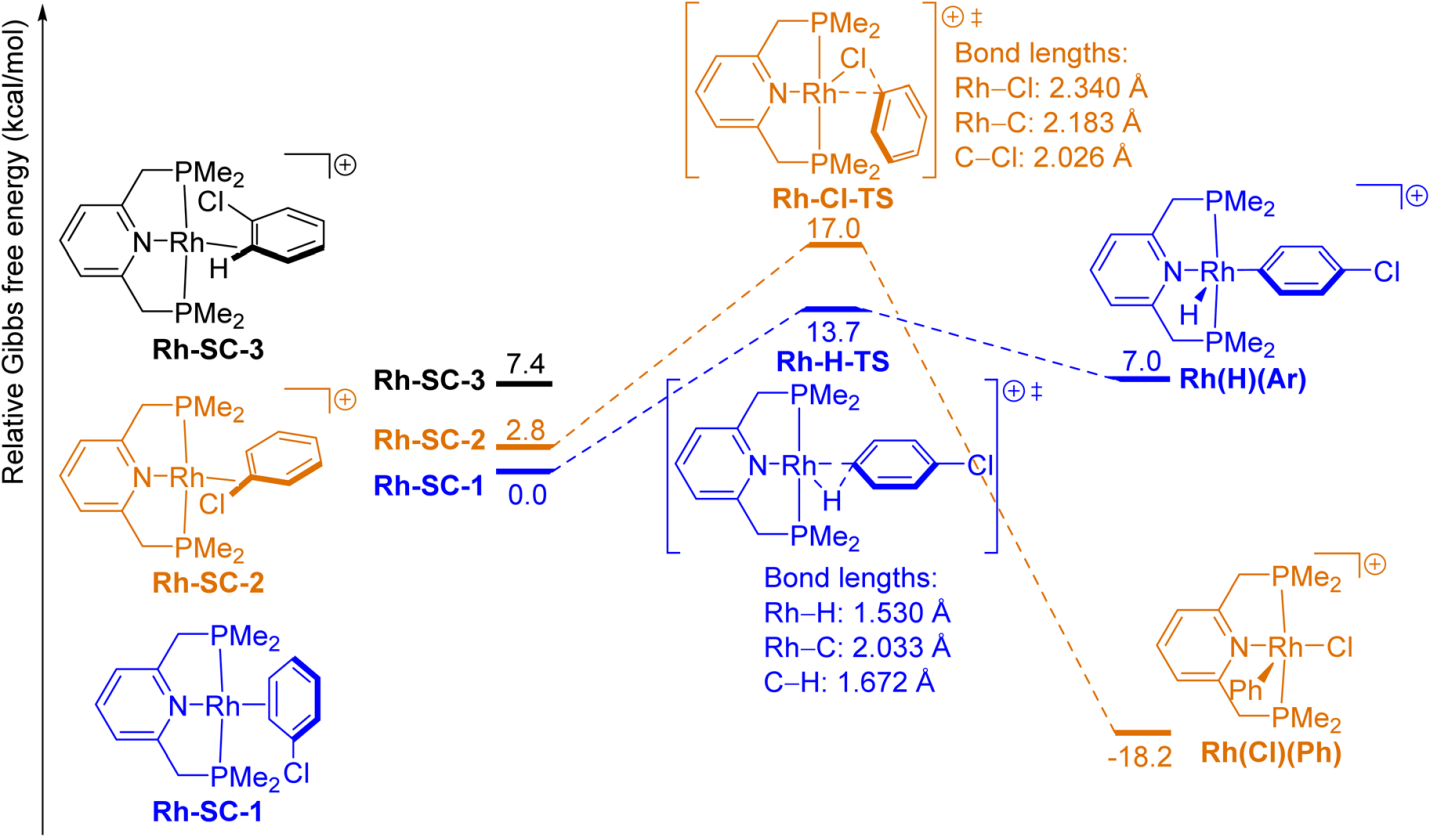
Free-energy diagram of the oxidative addition of the *para*-C–H bond (blue pathway) and the C–Cl bond (orange pathway) in PhCl to (L1)Rh(i) complexes.

**Fig. 3:**
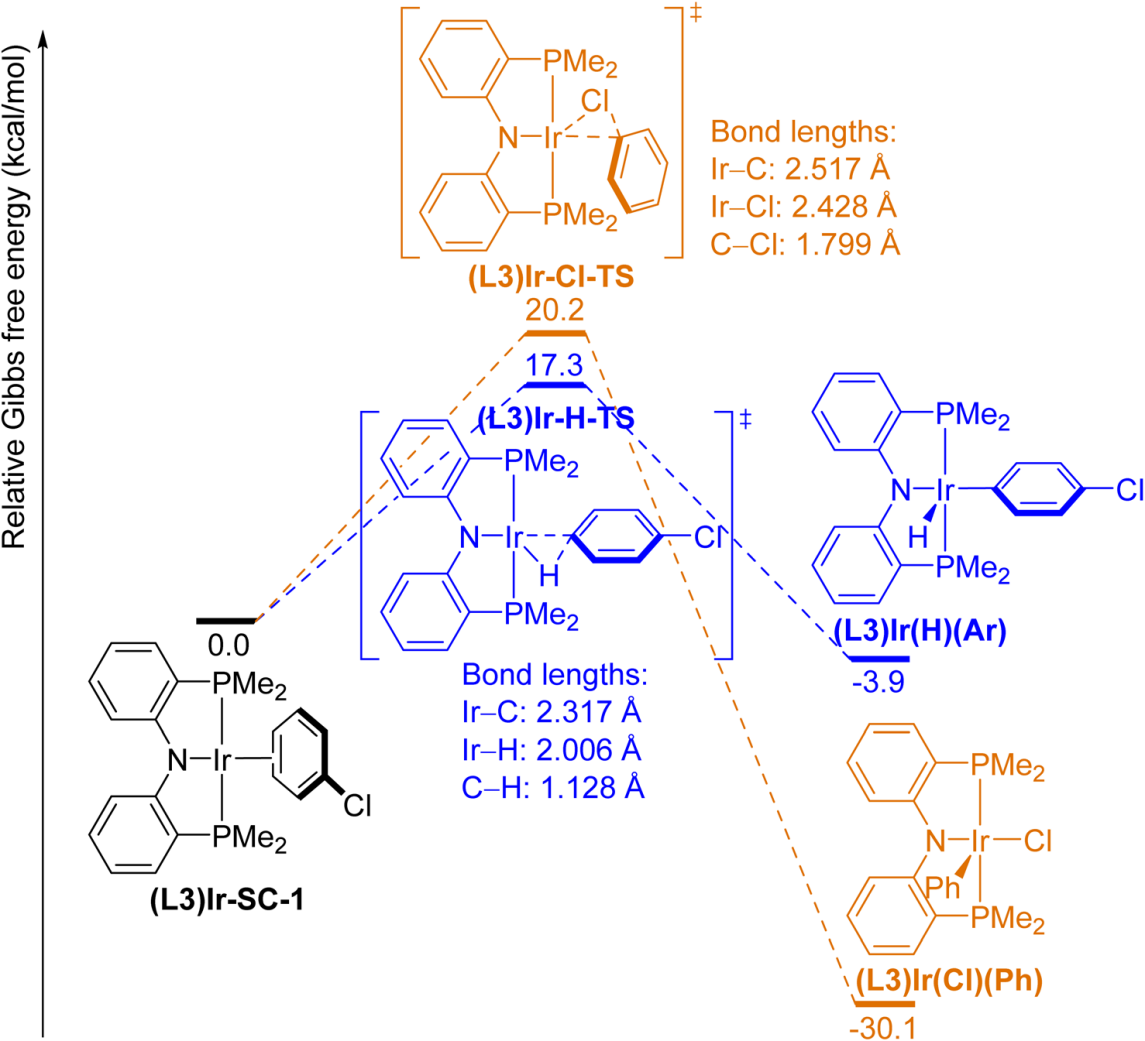
Free-energy diagram of the oxidative addition of the *para*-C–H bond (blue pathway) and the C–Cl bond (orange pathway) in PhCl to (L3)Ir(i) complexes.

**Fig. 4:**
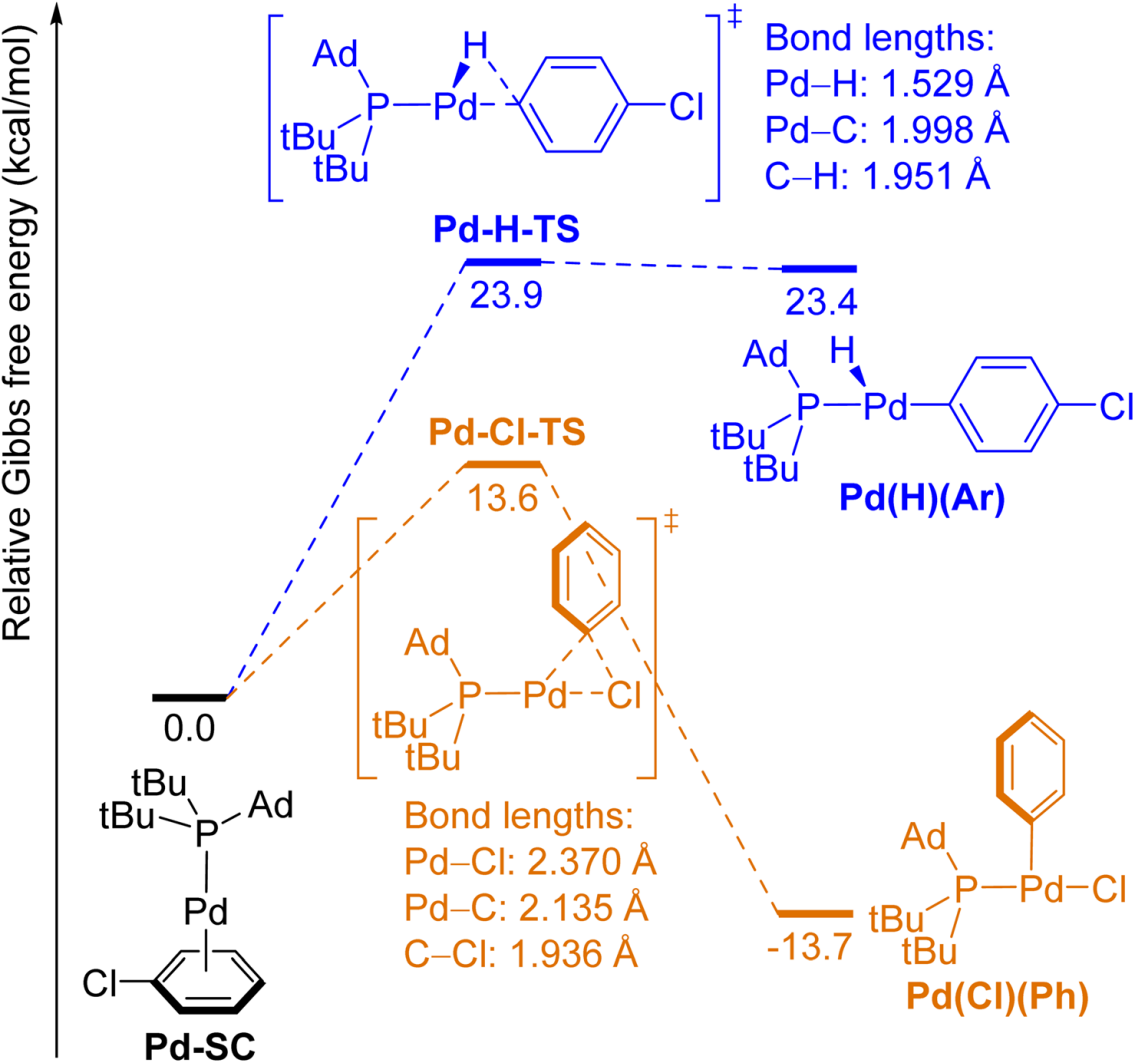
Free-energy diagram for the oxidative addition of the *para*-C–H bond (blue pathway) and the C–Cl bond (orange pathway) in PhCl to (L2)Pd(0) complexes.

**Fig. 5:**
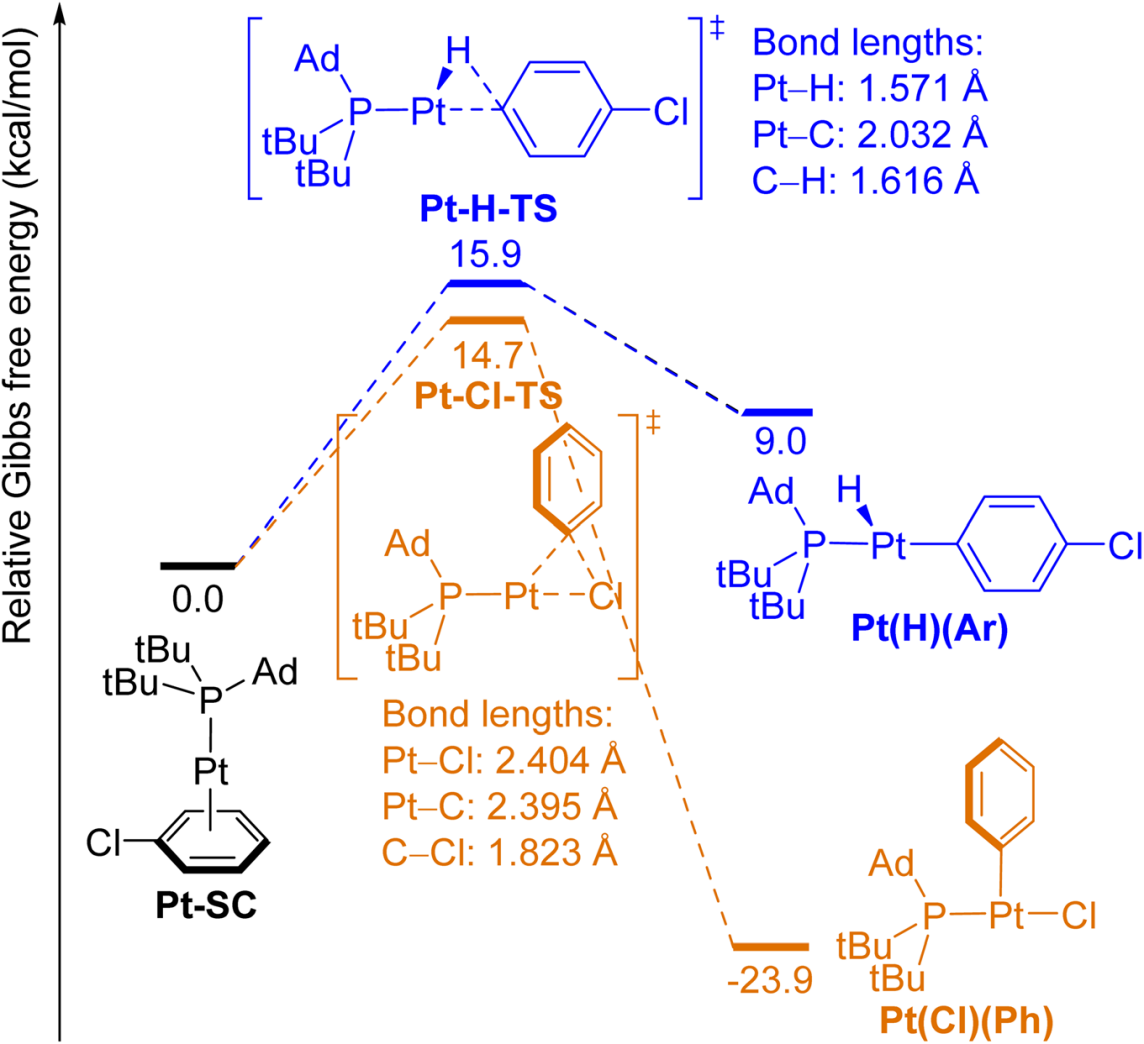
Free-energy diagram of the oxidative addition of the *para*-C–H bond (blue pathway) and the C–Cl bond (orange pathway) in PhCl to (L2)Pt(0) complexes.

We optimized the geometries and calculated the relative Gibbs free energies (Δ*G*) of the ground-state and the transition-state structures in these model oxidative additions. The free-energy diagrams of these reactions are shown in Fig. 1–5.

#### 2.2.1 Oxidative additions to cationic complexes of transition metals of Group 9

For reactions with (L1)Ir complexes, the η^2^-complex between the iridium center and the C(2)–C(3) double bond of PhCl is the lowest-energy structure ((L1)Ir-SC-1, Fig. 1, SC denotes “starting complex”) prior to the transition state for oxidative addition. This complex is usually formed by displacement of a labile L-type ligand (such as cyclooctene) on the iridium precursor by chlorobenzene.^46,48^ The σ-complex between the Ir center and the C–Cl bond of PhCl ((L1)Ir-SC-2, Fig. 1), which is 5.1 kcal mol^−1^ higher in energy than (L1)Ir-SC-1, was located as another local minimum geometry at this stage of the reaction coordinate. We computed the pathway for oxidative addition of the C–H bond that is *para* to the Cl atom in the PhCl within (L1)Ir-SC-1 to form the product (L1)Ir(H)(Ar). Although oxidative addition of the C–H bond that is *ortho* to the chlorine in PhCl to (L1)Ir(i) and (L1)Rh(i) complexes is kinetically and thermodynamically more favorable than addition of the *para*-C–H bond (see SI), we chose to study oxidative addition of the *para*-C–H bond because interactions between the chlorine and the metal center are negligible during this reaction and do not complicate our analysis of the C–H activation process.

The σ-complex (L1)Ir-SC-2 undergoes oxidative addition of the C–Cl bond to form the product (L1)Ir(Cl)(Ph), which adopts the square pyramidal geometry with the phenyl ligand occupying the axial position. The oxidative addition of the C–Cl bond to the cationic (L1)Ir complex (Δ*G*(Ir–Cl) = −26.5 kcal mol^−1^) is more exergonic than that of the C–H bond (Δ*G*(Ir–H) = −3.6 kcal mol^−1^), but the barrier to oxidative addition of the C–Cl bond to (L1)Ir (Δ*G*^‡^(Ir–Cl) = 18.6 kcal mol^−1^) is higher than that for oxidative addition of the C–H bond (Δ*G*^‡^(Ir–H) = 7.8 kcal mol^−1^, Fig. 1). The large difference between the barriers to oxidative additions of the C–H and the C–Cl bond (ΔΔ*G*^‡^ = 10.8 kcal mol^−1^) suggests that (L1)Ir^+^ undergoes solely oxidative addition of the C–H bond when reacting with PhCl. Such high selectivity of (L1)Ir complexes for oxidative addition of carbon–hydrogen bonds over carbon–halogen bonds was confirmed by experimental results on similar iridium complexes containing pincer ligands that are more sterically hindered than L1 (see SI) and are consistent with previously reported computational studies on similar complexes.^36,37,48,49^

The computed energies for the oxidative addition of the C–H and C–Cl bonds of chlorobenzene to the analogous cationic (L1)Rh(i) are shown in Fig. 2. Like the reactants (L1)Ir-SC-1 and (L1)Ir-SC-2, the π-complex between the Rh center and PhCl (Rh-SC-1), as well as the σ-complex between Rh and the C–Cl bond in PhCl (Rh-SC-2), were computed to be local minima prior to the transition states for oxidative additions involving (L1)Rh complexes. The σ-complex between Rh and the *ortho*-C–H bond in PhCl (Rh-SC-3) is a third stable complex in this portion of the reaction coordinate and was computed to lie 7.4 kcal mol^−1^ higher in free energy than Rh-SC-1.

The structures of the transition states and the products for the oxidative addition of C–H or C–Cl bonds to the cationic (L1)Rh fragment are similar to their Ir analogues. The oxidative additions of C–H and C–Cl bonds to (L1)Rh^+^ are less thermodynamically favorable than the oxidative additions to (L1)Ir^+^ (Δ*G*(Ir–H) = −3.6 kcal mol^−1^, Δ*G*(Rh–H) = 7.0 kcal mol^−1^; Δ*G*(Ir–Cl) = −26.5 kcal mol^−1^, Δ*G*(Rh–Cl) = −18.2 kcal mol^−1^), and these thermodynamic data are consistent with the known trend of weaker metal–hydrogen, metal–carbon and metal–halogen bonds to second-row, late transition metals than to third-row, late transition metals.^1^ In the case of late transition metals from Group 9, the Ir–H bond is stronger than the Rh–H bond because the former is more ionic than the latter.^50^ These computed data are also consistent with experimental reports that oxidative additions of C–H bonds to Rh(i) complexes are less thermodynamically favorable than those to analogous Ir(i) complexes.^51–54^ For example, the oxidative addition of aryl C–H bonds to a rhodium(i) complex typically requires a strong base, and stable Rh(iii)(H)(Ar) complexes are rare.^55–57^ In contrast, iridium(i) complexes readily cleave C–H bonds in the absence of bases, and several stable Ir(iii)(H)(Ar) complexes have been isolated.^46,58^ The computed barrier for oxidative addition of the aryl C–H bonds to the cationic (L1)Rh(i) is higher than that for oxidative addition to the cationic (L1)Ir(i) (Δ*G*^‡^(Ir–H) = 7.8 kcal mol^−1^, Δ*G*^‡^(Rh–H) = 13.7 kcal mol^−1^), whereas the barrier to oxidative addition of the C–Cl bond to (L1)Rh(i)^+^ is similar to that for oxidative addition to (L1)Ir(i)^+^ (Δ*G*^‡^(Ir–Cl) = 18.6 kcal mol^−1^, Δ*G*^‡^(Rh–Cl) = 17.0 kcal mol^−1^). The difference between the barriers to oxidative additions of a C–H and a C–Cl bond to (L1)Rh^+^ (ΔΔ*G*^‡^(Rh) = 3.3 kcal mol^−1^) is much smaller than the corresponding value for (L1)Ir^+^ (ΔΔ*G*^‡^(Ir) = 10.8 kcal mol^−1^), indicating that Rh(i) complexes are less selective for the oxidative addition of an aryl C–H bond over an aryl C–Cl bond than Ir(i) complexes are. Indeed, several rhodium(i) complexes were reported to cleave aryl–halide bonds over aryl C–H bonds^59–62^ because the difference between barriers is small and because oxidative addition of a C–H bond is less thermodynamically favorable than that of a carbon–halogen bond.

#### 2.2.2 Oxidative additions to a neutral Ir complex containing an LXL-type pincer ligand

Neutral Ir complexes containing LXL-type pincer ligands have been reported to undergo selective oxidative addition of aryl C–H bonds over C–Cl bonds.^37,58^ To determine the effects of the charge of the complex on the oxidative addition of C–Cl and C–H bonds, we computed the ground-state and transition-state structures for oxidative additions to the neutral (L3)Ir complex (Fig. 3), which is structurally closely related to the cationic (L1)Ir. The formal oxidation state of Ir in both (L1)Ir and (L3)Ir complexes is +1. The π-complex between the (L3)iridium center and the C(3)–C(4) bond in PhCl ((L3)-Ir-SC-1) was the lowest-energy structure located prior to the transition states. Stable σ-complexes between the metal center and a C–H or a C–Cl bond, which were located for the cationic Ir and Rh complexes ((L1)Ir-SC-2, Rh-SC-2, and Rh-SC-3), were not found for the neutral (L3)Ir complex. Oxidative addition of the C–Cl bond to (L3)Ir is more exergonic by 3.6 kcal mol^−1^ than to (L1)Ir^+^ (Δ*G*((L1)Ir–Cl) = −26.5 kcal mol^−1^, Δ*G*((L3)Ir–Cl) = −30.1 kcal mol^−1^), and the barrier to oxidative addition of the C–Cl bond to (L3)Ir is slightly higher than to (L1)Ir^+^ by 1.6 kcal mol^−1^ (Δ*G*^‡^((L3)Ir–Cl) = 20.2 kcal mol^−1^, Fig. 3; Δ*G*^‡^((L1)Ir–Cl) = 18.6 kcal mol^−1^, Fig. 1). Oxidative addition of the *para*-C–H bond in PhCl to the neutral (L3)Ir complex was computed to be exergonic by −3.9 kcal mol^−1^, and this value is similar to the corresponding value for the cationic (L1)Ir complex (Δ*G*((L1)Ir–H) = −3.6 kcal mol^−1^, Fig. 1). However, the computed barrier for oxidative addition of the C–H bond to the neutral (L3)Ir (Δ*G*^‡^((L3)Ir–H) = 17.3 kcal mol^−1^, Fig. 3) is significantly higher than the corresponding barrier for addition to (L1)Ir^+^ (Δ*G*^‡^((L1)Ir–H) = 7.8 kcal mol^−1^, Fig. 1). Indeed, the barrier for oxidative addition of the C–H bond to the neutral (L3)Ir is comparable to the barrier for oxidative addition of the C–Cl bond to the same (L3)Ir complex. These results are consistent with reported experimental data^46,58^ suggesting that the selectivity of cationic (PNP)Ir complexes for oxidative addition of aryl C–H bonds over C–X bonds is greater than that of the neutral (PNP)Ir complexes. The bond lengths of the C–H and the C–Cl bonds that are being cleaved in the transition states for the neutral (L3)Ir complex are shorter than those in the transition states for the cationic (L1)Ir (C–H bond length in (L3)Ir-H-TS = 1.128 Å, C–H bond length in (L1)Ir-H-TS = 1.436 Å; C–Cl bond length in (L3)Ir-Cl-TS = 1.799 Å, C–Cl bond length in (L1)Ir-Cl-TS = 1.909 Å), whereas the distances between Ir and the two atoms in the bond that is being broken in the transition states for the reactions of (L3)Ir are longer than those in the transition states for reactions of (L1)Ir^+^. These results suggest that the transition states for oxidative additions to the neutral (L3)Ir complex are earlier than those to the cationic (L1)Ir complex, especially for the oxidative addition of the C–H bond.

#### 2.2.3 Oxidative additions to complexes of transition metals of Group 10

The computed energies for oxidative addition of the *para*-C–H bond and the C–Cl bond in PhCl to (L2)Pd(0) are shown in Fig. 4. The complex between (L2)Pd and the π-bond between C(2) and C(3) in PhCl (Pd-SC) was computed to be the lowest-energy structure prior to the transition states for either type of oxidative addition. This complex is formed by substitution of one L2 ligand in (L2)_2_Pd, which is the resting state of the catalytic reaction, by chlorobenzene.^47^ Oxidative addition of the C–Cl bond to Pd forms the three-coordinate, T-shaped product Pd(Cl)(Ph), in which the phosphine and the chloride ligands are *trans* to each other. This reaction is exergonic (Δ*G*(Pd–Cl) = −13.7 kcal mol^−1^) and is computed to occur with a relatively low barrier (Δ*G*^‡^(Pd–Cl) = 13.6 kcal mol^−1^). In contrast, oxidative addition of the *para* C–H bond to (L2)Pd(0) is endergonic (Δ*G*(Pd–H) = 23.4 kcal mol^−1^), and the barrier to oxidative addition of the C–H bond (Δ*G*^‡^(Pd–H) = 23.9 kcal mol^−1^) is significantly higher than that of the C–Cl bond (ΔΔ*G*^‡^ = 10.3 kcal mol^−1^). In agreement with Hammond's postulate, the structure of transition state Pd-H-TS is similar to that of the product Pd(H)(Ar). The small difference in energy (0.5 kcal mol^−1^) between Pd-H-TS and Pd(H)(Ar) also implies that reductive elimination to form the C–H bond from the arylpalladium hydride is nearly barrierless. The structures of reactant complexes, transition states, and products of the oxidative addition of C–H and C–Cl bonds in PhCl to the (L2)Pt(0) unit (Fig. 5) are similar to those of the Pd counterparts (shown in Fig. 4). Oxidative addition of the C–H bond to (L2)Pt(0) is less endergonic than that to (L2)Pd(0) (Δ*G*(Pt–H) = 9.0 kcal mol^−1^, Δ*G*(Pd–H) = 23.4 kcal mol^−1^), and oxidative addition of the C–Cl bond to Pt is more exergonic than that to Pd (Δ*G*(Pt–Cl) = −23.9 kcal mol^−1^, Δ*G*(Pd–Cl) = −13.7 kcal mol^−1^). Like the energetic differences between reactions with Rh and Ir complexes (*vide supra*), these data agree with the trend that the metal–hydrogen, metal–carbon and metal–halogen bonds to second-row, late transition metals are weaker than those to third-row late transition metals.^1^ The computed barrier for oxidative addition of the C–H bond to the (L2)Pt(0) complex is lower than that to (L2)Pd(0) (Δ*G*^‡^(Pt–H) = 15.9 kcal mol^−1^, Δ*G*^‡^(Pd–H) = 23.9 kcal mol^−1^), whereas the barrier for oxidative addition of the C–Cl bond to (L2)Pt(0) is similar to that to (L2)Pd(0) (Δ*G*^‡^(Pt–Cl) = 14.7 kcal mol^−1^, Δ*G*^‡^(Pd–Cl) = 13.6 kcal mol^−1^) even though oxidative addition of the C–Cl bond to Pt is more exergonic than that to Pd.

### 2.3 Thermodynamics of the oxidative additions and correlations with bond strengths

To investigate the correlation between the strengths of the bonds that are broken or formed during the oxidative additions to (L1)Ir (L3)Ir (L1)Rh (L2)Pd, and (L2)Pt complexes and the thermodynamics of these reactions, we computed the vertical bond dissociation energies (vBDEs) of the C–H, C–Cl, M–H, and M–Cl bonds that are broken or formed during these reactions. These results are summarized in Table 1. Vertical bond dissociation energies (vBDEs) are equivalent to the interaction energy in a distortion/interaction analysis of single bonds and, therefore, provide an estimate of the relative strengths of chemical bonds and thermodynamics of the reactions. These data show that the vBDE of a C(aryl)–H bond is higher than that of a C(aryl)–Cl bond (entries 21–22), whereas the vBDE of a metal–hydrogen bond is lower than that of a metal–chlorine bond for each metal. These data are consistent with our computational results showing that oxidative addition of a C(aryl)–H bond to a metal center is less exergonic than that of a C(aryl)–Cl bond. Data from Table 1 indicate that the vBDEs of M–H and M–Cl bonds for third-row transition metals (Ir, Pt) are higher than those for second-row metals (Rh, Pd). This trend is consistent with our computations showing that oxidative additions of C–H or C–Cl bonds to (L1)Ir are more exergonic than to (L1)Rh and that oxidative additions of these bonds to (L2)Pt are more thermodynamically favorable than to (L2)Pd. Electronegativities of elements in different oxidation states and with different coordinate numbers have been evaluated^63^ and computational studies suggested that a more electronegative transition metal generally forms a stronger M–H bond than a less electronegative transition metal does.^50^ Our vBDE values are consistent with this trend since Ir(iii) (*χ* = 1.664) is more electronegative than Rh(iii) (*χ* = 1.622) and Pt(ii) (*χ* = 1.432) is more electronegative than Pd(ii) (*χ* = 1.346). Specifically, the Pd–H bond (entry 14) is the weakest among all calculated metal–hydrogen bonds and the Pd–C(Ar) and Pd–C(Ph) bonds (entries 13 and 15) are the weakest among all calculated M–C(Ar) and M–C(Ph) bonds, respectively. Such weakness of Pd–H and Pd–C bonds is consistent with the high endergonicity of oxidative addition of an aryl C–H bond to (L2)Pd.

**Table 1:** Computed vertical bond dissociation energies (vBDEs) of C–H, C–Cl, M–H, and M–Cl bonds that are broken or formed during reactions with Rh, Ir, Pd, and Pt complexes

Entry	Compound	Bond	vBDE (kcal mol^−1^)
1	(L1)Ir(H)(Ar)	Ir–C(Ar)	98.9
2	(L1)Ir(H)(Ar)	Ir–H	81.5
3	(L1)Ir(Cl)(Ph)	Ir–C(Ph)	78.6
4	(L1)Ir(Cl)(Ph)	Ir–Cl	107.4
5	(L3)Ir(H)(Ar)	Ir–C(Ar)	95.4
6	(L3)Ir(H)(Ar)	Ir–H	76.4
7	(L3)Ir(Cl)(Ph)	Ir–C(Ph)	73.5
8	(L3)Ir(Cl)(Ph)	Ir–Cl	111.0
9	Rh(H)(Ar)	Rh–C(Ar)	84.7
10	Rh(H)(Ar)	Rh–H	71.4
11	Rh(Cl)(Ph)	Rh–C(Ph)	69.5
12	Rh(Cl)(Ph)	Rh–Cl	97.1
13	Pd(H)(Ar)	Pd–C(Ar)	78.3
14	Pd(H)(Ar)	Pd–H	65.4
15	Pd(Cl)(Ph)	Pd–C(Ph)	64.0
16	Pd(Cl)(Ph)	Pd–Cl	103.5
17	Pt(H)(Ar)	Pt–C(Ar)	93.3
18	Pt(H)(Ar)	Pt–H	82.0
19	Pt(Cl)(Ph)	Pt–C(Ph)	83.7
20	Pt(Cl)(Ph)	Pt–Cl	110.5
21	PhCl	C–Cl	101.9
22	PhCl	*para*-C–H	120.1

### 2.4 Energy decomposition analysis of transition states and ground states

To investigate the factors that influence the barriers to oxidative addition of C–H and C–Cl bonds in PhCl to complexes containing Rh, Ir, Pd, and Pt, we conducted energy decomposition analysis (EDA) of the transition states and reactants by partitioning each structure into two fragments: one containing chlorobenzene and the other containing the metal center and the ligand L1, L2 or L3. The results are summarized in Tables 2 and 3. In the following subsections, we rationalize the trends in barriers to oxidative addition based on the data from EDA.

**Table 2:** Results from energy decomposition analysis of the ground states and transition states of oxidative addition of the C–H and C–Cl bonds in chlorobenzene to complexes of Ir, Rh, Pd, and Pt^a^

Structure	Δ*E*_FRZ_	Δ*E*_POL_	Δ*E*_CT_	Δ*E*_M→L_	Δ*E*_L→M_	Δ*E*_INT_	Δ*E*^PhCl^_GD_	ΔE^(**L**)M^_GD_	Δ*E*_GD_ + Δ*E*_INT_
(L1)Ir-SC-1	74.6	−54.8	−76.7	−37.0	−33.1	−56.9	12.3	4.7	−39.9
(L1)Ir-H-TS	98.5	−72.4	−97.8	−42.3	−43.1	−71.7	40.6	5.6	−25.5
(L1)Ir-Cl-TS	67.2	−41.2	−63.6	−33.4	−26.5	−37.6	13.4	4.4	−19.7
(L3)Ir-SC-1	94.6	−64.1	−90.0	−52.3	−28.4	−59.5	16.3	2.6	−40.7
(L3)Ir-H-TS	34.1	−24.7	−34.2	−13.7	−15.5	−24.8	1.6	1.3	−21.8
(L3)Ir-Cl-TS	42.1	−25.3	−42.0	−19.6	−17.1	−25.3	3.4	3.4	−18.4
Rh-SC-1	30.2	−21.6	−51.4	−25.9	−21.9	−42.8	7.8	2.7	−32.3
Rh-H-TS	61.5	−43.4	−102.3	−53.6	−36.0	−84.3	69.0	3.6	−11.7
Rh-Cl-TS	51.1	−26.8	−64.3	−40.2	−20.3	−40.0	24.1	3.5	−12.5
Pd-SC	29.3	−15.9	−44.4	−30.8	−11.6	−31.0	3.7	1.6	−25.8
Pd-H-TS	60.9	−58.9	−94.7	−63.0	−24.7	−92.6	93.0	3.9	4.3
Pd-Cl-TS	43.6	−18.7	−55.5	−42.4	−10.9	−30.6	16.2	1.6	−12.8
Pt-SC	64.0	−37.7	−65.3	−38.2	−22.6	−38.9	5.9	3.9	−29.1
Pt-H-TS	108.4	−82.9	−102.5	−56.5	−36.4	−77.0	59.3	7.8	−9.9
Pt-Cl-TS	39.3	−20.7	−42.1	−24.3	−15.9	−23.4	5.3	1.4	−16.7

a All energies are reported in kcal mol^−1^. Abbreviations: Δ*E*_FRZ_ = change in energy attributable to the frozen density term; Δ*E*_POL_ = change in energy attributable to the polarization term; Δ*E*_CT_ = change in energy attributable to the charge transfer term; Δ*E*_M→L_ = the part of Δ*E*_CT_ caused by charge transfer from the metal-containing fragment to the PhCl fragment; Δ*E*_L→M_ = the part of Δ*E*_CT_ caused by charge transfer from PhCl to the metal-containing fragment; Δ*E*_INT_ = Δ*E*_FRZ_ + Δ*E*_POL_ + Δ*E*_CT_, the total interaction energy; Δ*E*^PhCl^_GD_ = destabilization caused by distorting the geometry of the PhCl fragment from its isolated, lowest-energy ground-state structure; “GD” stands for geometry distortion; Δ*E*^(L)M^_GD_ = destabilization caused by distorting the geometry of the (L)M (L = L1, L2, or L3, M = Ir, Rh, Pt, or Pd) fragment from its isolated, lowest-energy ground-state structure.

**Table 3:** Difference in each EDA term between the transition state to oxidative addition of the C–H bond and that of the C–Cl bond^a^

Process	ΔΔ*E*_FRZ_	ΔΔ*E*_POL_	ΔΔ*E*_CT_	ΔΔ*E*_M→L_	ΔΔ*E*_L→M_	ΔΔ*E*_INT_	ΔΔ*E*^PhCl^_GD_	ΔΔ*E*^(**L**)M^_GD_	ΔΔ*E*_GD_ + ΔΔ*E*_INT_ (=ΔΔ*E*^‡^)
(L1)Ir-H-TS → (L1)Ir-Cl-TS	−31.3	31.2	34.2	8.9	16.6	34.1	−27.2	−1.2	5.8
(L3)Ir-H-TS → (L3)Ir-Cl-TS	7.9	−0.6	−7.8	−5.8	−1.6	−0.5	1.8	2.1	3.4
Rh-H-TS → Rh-Cl-TS	−10.4	16.6	38.0	13.4	15.7	44.3	−44.9	−0.1	−0.8
Pd-H-TS → Pd-Cl-TS	−17.3	40.2	39.2	20.6	13.8	62.0	−76.8	−2.3	−17.1
Pt-H-TS → Pt-Cl-TS	−69.1	62.2	60.4	32.2	20.5	53.6	−54.0	−6.4	−6.8

a All energies are reported in kcal mol^−1^. Abbreviations: ΔΔ*E*_FRZ_ = energy change attributable to the frozen density term; ΔΔ*E*_POL_ = energy change attributable to the polarization term; ΔΔ*E*_CT_ = energy change attributable to the charge transfer term; ΔΔ*E*_M → L_ = the part of ΔΔ*E*_CT_ caused by charge transfer from the metal-containing fragment to the PhCl fragment; ΔΔ*E*_L → M_ = the part of ΔΔ*E*_CT_ caused by charge transfer from PhCl to the metal-containing fragment; ΔΔ*E*_INT_ = ΔΔ*E*_FRZ_ + ΔΔ*E*_POL_ + ΔΔ*E*_CT_, the total interaction energy; ΔΔ*E*^PhCl^_GD_ = destabilization caused by distorting the geometry of the PhCl fragment from the C–H oxidative addition TS geometry to the C–Cl oxidative addition TS geometry; “GD” stands for geometry distortion; ΔΔ*E*^(L)M^_GD_ = destabilization caused by distorting the geometry of the (L)M (L = L1, L2, or L3, M = Ir, Rh, Pt, or Pd) fragment from the C–H oxidative addition TS geometry to the C–Cl oxidative addition TS geometry.

#### 2.4.1 Barriers to oxidative addition of C(aryl)–H bonds to complexes of second-row transition metals are higher than those to complexes of third-row transition metals because the PhCl fragment in transition states of second-row metals is highly distorted

A comparison between Fig. 1 and 2 indicates that the barrier to oxidative addition of the aryl C–H bond to the cationic (L1)Rh is 5.9 kcal mol^−1^ higher than that to the analogous (L1)Ir. Likewise, a comparison between Fig. 4 and 5 indicates that the barrier to oxidative addition of the aryl C–H bond to (L2)Pd is 8.0 kcal mol^−1^ higher than that to the analogous (L2)Pt. Energy decomposition analysis of the transition states suggests that the C–H bond in the PhCl fragment in the transition states for reactions with second-row metals is more distorted than it is with third-row metals and that this relative degree of distortion is the primary cause of the difference in barrier for oxidative addition to second-row transition metals *versus* third-row metals. For example, the difference between interaction energies of transition states (L1)Ir-H-TS and Rh-H-TS (Δ*E*_INT_((L1)Ir-H-TS) = −71.7 kcal mol^−1^, Δ*E*_INT_((L1)Rh-H-TS) = −84.3 kcal mol^−1^, a difference of 12.6 kcal mol^−1^) is much smaller than the difference between distortion energies of the PhCl fragment in Rh-H-TS and (L1)Ir-H-TS (Δ*E*^PhCl^_GD_(Rh-H-TS) = 69.0 kcal mol^−1^, Δ*E*^PhCl^_GD_((L1)Ir-H-TS) = 40.6 kcal mol^−1^, a difference of 28.4 kcal mol^−1^). The interaction energy between the PhCl fragment and the (L2)M fragment in Pd-H-TS is 15.6 kcal mol^−1^ more stabilizing than that in Pt-H-TS (Δ*E*_INT_(Pd-H-TS) = −92.6 kcal mol^−1^, Δ*E*_INT_(Pt-H-TS) = −77.0 kcal mol^−1^) but the destabilization caused by distortion of the PhCl fragment in Pd-H-TS is 33.7 kcal mol^−1^ larger than that in Pt-H-TS (Δ*E*^PhCl^_GD_(Pd-H-TS) = 93.0 kcal mol^−1^, Δ*E*^PhCl^_GD_(Pt-H-TS) = 59.3 kcal mol^−1^). For transition metals from either Group 9 or Group 10, the difference in Δ*E*^PhCl^_GD_ between second-row and third-row metals is much larger than the difference in Δ*E*_INT_ or Δ*E*^(L)M^_GD_ and, therefore, is the main contributor to the difference in barrier to oxidative addition for second-row *versus* third-row transition metals.

As shown in Table 4, the *para*-C–H bond in PhCl that is being cleaved in transition state Rh-H-TS is longer than that in (L1)Ir-H-TS (entries 2–3), and the same bond that is being cleaved in transition state Pd-H-TS is longer than that in Pt-H-TS (entries 4–5). The greater distance between the carbon and the hydrogen atoms undergoing oxidative addition in the transition states involving second-row metals is consistent with the greater degree of distortion of the PhCl fragment in these structures, as determined from energy decomposition analysis. These results indicate that the transition states to oxidative addition of the aryl C–H bond to second-row transition metals are later in the reaction coordinate than those to third-row transition metals. These trends are consistent with the periodic trend in polarizability of late transition metals. We propose that less elongation of the C–H bond in PhCl is required to achieve the greatest overlap between the σ* orbital of the C–H bond and the d orbitals on the Ir or Pt centers than is required for that on the Rh or Pd centers because a third-row transition metal is more polarizable than a second-row transition metal.

**Table 4:** Bond Length of the *para*-C–H bond in the PhCl fragment in computed structures

Entry	Structure	*para*-C–H bond length (Å)
1	PhCl	1.092
2	Rh-H-TS	1.672
3	(L1)Ir-H-TS	1.436
4	Pd-H-TS	1.951
5	Pt-H-TS	1.616

#### 2.4.2 The electrophilicity of Ir(i) leads to high selectivity of Ir complexes for oxidative addition of C–H bonds over C–Cl bonds

Energy decomposition analysis of transition states (L1)Ir-H-TS and (L1)Ir-Cl-TS suggests that the PhCl fragment in (L1)Ir-H-TS is more distorted than that in (L1)Ir-Cl-TS (Δ*E*^PhCl^_GD_((L1)Ir-H-TS) = 40.6 kcal mol^−1^, Δ*E*^PhCl^_GD_((L1)Ir-Cl-TS) = 13.4 kcal mol^−1^), whereas the total interaction energy between the PhCl fragment and the (L1)Ir fragment in (L1)Ir-H-TS is more negative, *i.e.*, more stabilizing, than that in (L1)Ir-Cl-TS (Δ*E*_INT_((L1)Ir-H-TS) = −71.7 kcal mol^−1^, Δ*E*_INT_((L1)Ir-Cl-TS) = −37.6 kcal mol^−1^). These data suggest that (L1)Ir-H-TS is a later transition state than (L1)Ir-Cl-TS is. Similar analyses of the Δ*E*^PhCl^_GD_ and the Δ*E*_INT_ term for transition states containing (L1)Rh (L2)Pd, and (L2)Pt (see Table 2) reveal the same trend: the transition states for oxidative addition of an aryl C–H bond to these complexes are later than those for oxidative addition of an aryl C–Cl bond.

The difference between the interaction energies of the two transition states containing (L1)Ir (Δ*E*_INT_((L1)Ir-H-TS) – Δ*E*_INT_((L1)Ir-Cl-TS) = −34.1 kcal mol^−1^) is larger than the difference between the distortion energy of these transition states (Δ*E*^PhCl^_GD_((L1)Ir-H-TS) – Δ*E*^PhCl^_GD_((L1)Ir-Cl-TS) = 27.2 kcal mol^−1^). Therefore, significant stabilization afforded by electronic interactions between the Ir center and the aryl C–H bond that is being cleaved in the transition state contributes to the low barrier to oxidative addition of aryl C–H bonds to Ir complexes. Such stabilizing electronic interactions are partially reflected in Δ*E*_M→L_ and Δ*E*_L→M_, which represent stabilization caused by charge transfer from the metal to the arene and from the arene to the metal, respectively. As shown in Table 2, Δ*E*_M→L_((L1)Ir-H-TS) is 8.9 kcal mol^−1^ more stabilizing than Δ*E*_M→L_((L1)Ir-Cl-TS), and Δ*E*_L→M_((L1)Ir-H-TS) is 16.6 kcal mol^−1^ more stabilizing than Δ*E*_L→M_((L1)Ir-Cl-TS). This comparison indicates that the interactions between d orbitals on Ir and the σ and σ* orbitals of the C–H bond are more stabilizing than those between d orbitals on Ir and the orbitals of the C–Cl bond in the two transition states.

To understand why orbital interactions between Ir and a C–H bond are more stabilizing than those between Ir and a C–Cl bond, we conducted natural population analysis of ground states and transition states for the oxidative additions of C–H and C–Cl bonds to the cationic (L1)Ir complex. Results from such analysis indicate that the partial charge on Ir changes from −0.02 ((L1)Ir-SC-1) to −0.12 ((L1)Ir-H-TS), and then to +0.10 ((L1)Ir(H)(Ar)) during oxidative addition of the C–H bond, and the sum of partial charges on the carbon and hydrogen atoms of the C–H bond that is being cleaved changes from −0.10 ((L1)Ir-SC-1) to +0.04 ((L1)Ir-H-TS), and then to −0.09 ((L1)Ir(H)(Ar), Table 5, left). These results suggest that transfer of electron density from the occupied σ orbital of the C–H bond to the vacant d orbitals of Ir occurs before the transfer of electron density from the occupied d orbital of Ir to the vacant σ* orbital of the C–H bond, and the Ir center contains partial negative charge in the transition state, despite the overall +1 charge of the whole complex. We propose that such transfer of electron density from the C–H bond to the positively charged, electrophilic (L1)Ir fragment stabilizes the transition state for oxidative addition of the C–H bond.

**Table 5:** Natural charges of atoms in the ground states and transition states for oxidative addition of C–H and C–Cl bonds to (L1)Ir complexes

Structure	Atom^a^	Charge^b^	Structure	Atom^a^	Charge^b^
(L1)Ir-SC-1	Ir	−0.02	(L1)Ir-SC-2	Ir	−0.24
	α-H	0.25		Cl	0.17
	α-C(Ar)	−0.35		α-C(Ar)	−0.05
(L1)Ir-H-TS	Ir	−0.12	(L1)Ir-Cl-TS	Ir	0.00
	α-H	0.25		Cl	0.03
	α-C(Ar)	−0.21		α-C(Ar)	−0.02
(L1)Ir(H)(Ar)	Ir	0.10	(L1)Ir(Cl)(Ph)	Ir	0.37
	α-H	0.15		Cl	−0.46
	α-C(Ar)	−0.24		α-C(Ar)	−0.07

a α-C(Ar) refers to the carbon atom in PhCl that is bound to Ir in the product (L1)Ir(H)(Ar) or (L1)Ir(Cl)(Ph); α-H refers to the hydrogen atom that is bound to α-C(Ar) in PhCl.

b Charge is determined by natural population analysis, and is reported in units of the elementary charge (*e*).

Similar analysis of the oxidative addition of a C–Cl bond to (L1)Ir shows that the partial charge on Ir decreases from −0.02 ((L1)Ir-SC-1) to −0.24 ((L1)Ir-SC-2) as the reaction progresses from the lowest-energy reactant (L1)Ir-SC-1 to the σ-complex (L1)Ir-SC-2. Between (L1)Ir-SC-2 and the product of oxidative addition, the partial charge on Ir increases monotonically from −0.24 ((L1)Ir-SC-2) to 0.00 ((L1)Ir-Cl-TS), and then to 0.37 ((L1)Ir(Cl)(Ph)), whereas the partial charge on Cl decreases monotonically from 0.17 ((L1)Ir-SC-2) to 0.03 ((L1)Ir-Cl-TS), and then to −0.46 ((L1)Ir(Cl)(Ph)), and the partial charge on carbon undergoes little change (Table 5, right). The more negative partial charge on Ir (−0.24) in (L1)Ir-SC-2 than that in (L1)Ir-SC-1 (−0.02) and the partial positive charge on Cl (+0.17) in the σ-complex (L1)Ir-SC-2 are attributed to coordination of Cl to the Ir center. The large difference in partial charges on Ir and Cl in the product (L1)Ir(Cl)(Ph) (+0.37 for Ir, −0.46 for Cl) suggests that this Ir–Cl bond is partially ionic in character, consistent with the large BDE of Ir–Cl bonds (Table 1, entry 4) and the exergonicity of oxidative addition of C–Cl bonds. Such changes in partial charges suggest that, unlike oxidative addition of the C–H bond, transfer of electron density from the Cl atom to the cationic (L1)Ir occurs before the step that breaks the C–Cl bond. During the bond-breaking step, transfer of electron density from Ir to the σ* orbital of the C–Cl bond is not accompanied by transfer of electron density to Ir, and we propose that this loss of electron density from the electrophilic (L1)Ir fragment destabilizes the transition state and accounts for the high barrier to oxidative addition of C–Cl bonds to (L1)Ir complexes.

In summary, comparing the oxidative additions of the aryl C–H bond and the aryl C–Cl bond to (L1)Ir^+^, we attribute the low barrier of oxidative addition of the C–H bond to the matching polarity of the electrophilic Ir(i) atom and the electron-rich C–H bond. In contrast, the α-C(Ar) atom in the C–Cl bond is electrophilic because Cl is more electronegative than C, resulting in a mismatch of polarity between the electrophilic Ir(i) atom and the electrophilic α-C(Ar) atom, and, thus, a higher activation barrier.

#### 2.4.3 Effects of the overall charge of the Ir complex on oxidative addition

Energy decomposition analysis shows that interactions caused by charge transfer between the iridium complex and the PhCl fragment (Δ*E*_CT_) in the neutral ground state (L3)Ir-SC-1 are more stabilizing than those in the cationic ground state (L1)Ir-SC-1 by 13.3 kcal mol^−1^. Specifically, Δ*E*_M→L_((L3)Ir-SC-1) is 15.3 kcal mol^−1^ more negative than Δ*E*_M→L_((L1)Ir-SC-1), suggesting that backbonding from d-orbitals of the neutral (L3)Ir to the π-system of PhCl is more stabilizing than that from d orbitals of the cationic (L1)Ir. The PhCl fragment in the neutral transition states (L3)Ir-H-TS and (L3)Ir-Cl-TS is significantly less distorted than that in their cationic counterparts, (L1)Ir-H-TS and (L1)Ir-Cl-TS, suggesting that (L3)Ir-H-TS and (L3)Ir-Cl-TS are earlier transition states than (L1)Ir-H-TS and (L1)Ir-Cl-TS. This conclusion is consistent with our analysis based on bond lengths in the transition states (see Section 2.2.2). The interaction energy in (L3)Ir-Cl-TS is less stabilizing than that in (L1)Ir-Cl-TS (Δ*E*_INT_((L3)Ir-Cl-TS) = −25.3 kcal mol^−1^, Δ*E*_INT_((L1)Ir-Cl-TS) = −37.6 kcal mol^−1^; a difference of 12.3 kcal mol^−1^) whereas the interaction energy in (L3)Ir-H-TS is significantly less stabilizing than that in (L1)Ir-H-TS (Δ*E*_INT_((L3)Ir-H-TS) = −24.8 kcal mol^−1^, Δ*E*_INT_((L1)Ir-H-TS) = −71.7 kcal mol^−1^; a difference of 46.9 kcal mol^−1^), consistent with the significantly higher barrier to oxidative addition of the C–H bond to (L3)Ir than that to (L1)Ir^+^.

To understand why the interaction energy in the neutral (L3)Ir-H-TS is significantly less stabilizing than that in the cationic (L1)Ir-H-TS, and to understand why transition states for (L3)Ir are earlier than those for (L1)Ir^+^, we conducted natural population analysis on (L3)Ir structures and the results are summarized in Table 6.

**Table 6:** Natural charges of atoms in the ground states and transition states for oxidative addition of C–H and C–Cl bonds to (L3)Ir complexes

Structure	Atom^a^	Charge^b^	Structure	Atom^a^	Charge^b^
(L3)Ir-SC-1	Ir	0.06	(L3)Ir-SC-1	Ir	0.06
	α-H	0.22		Cl	0.00
	α-C(Ar)	−0.18		α-C(Ar)	0.01
(L3)Ir-H-TS	Ir	−0.16	(L3)Ir-Cl-TS	Ir	−0.09
	α-H	0.27		Cl	0.09
	α-C(Ar)	−0.35		α-C(Ar)	−0.03
(L3)Ir(H)(Ar)	Ir	0.08	(L3)Ir(Cl)(Ph)	Ir	0.39
	α-H	0.11		Cl	−0.55
	α-C(Ar)	−0.17		α-C(Ar)	−0.06

a α-C(Ar) refers to the carbon atom in PhCl that is bound to Ir in the product (L3)Ir(H)(Ar) or (L3)Ir(Cl)(Ph); α-H refers to the hydrogen atom that is bound to α-C(Ar) in PhCl.

b Charge is determined by natural population analysis, and is reported in units of the elementary charge (*e*).

During oxidative addition of the C–H bond to the neutral (L3)Ir, the partial charge on Ir changes from +0.06 ((L3)Ir-SC-1) to −0.16 ((L3)Ir-H-TS), then to +0.08 ((L3)Ir(H)(Ar)). During oxidative addition of the C–Cl bond, the partial charge on Ir changes from +0.06 ((L3)Ir-SC-1) to −0.09 ((L3)Ir-Cl-TS), then to +0.39 ((L3)Ir(Cl)(Ph)). Data from Tables 5 and 6 suggest that electron density is transferred from the PhCl fragment to the Ir center before it is donated to the PhCl fragment from the Ir center during oxidative addition of the C–H or the C–Cl bond to both the cationic (L1)Ir complex and the neutral (L3)Ir complex. We hypothesize that the accumulation of partial negative charge on Ir in the transition states can be attributed to two processes: (1) the π-system in PhCl dissociates from the Ir center as the π-complex (L1)Ir-SC-1 or (L3)Ir-SC-1 is converted to the transition state, and the electron density that was originally donated from the d orbitals of Ir to the π* orbitals of PhCl is transferred back to the Ir center; (2) the σ orbital of the C–H bond or the lone pair on Cl donates electron density to the Ir center. Because the neutral (L3)Ir complex is less electrophilic (more electron-rich) than the cationic (L1)Ir^+^ complex, transfer of electron density from PhCl to (L3)Ir is less stabilizing than that to (L1)Ir^+^ and donation of electron density from (L3)Ir to PhCl is more stabilizing than that from (L1)Ir^+^. Consistent with our analysis, the stable σ-complex (L1)Ir-SC-2, in which the chlorine atom coordinates to the cationic iridium center, was located prior to the transition state but an analogous σ-complex for the neutral (L3)Ir could not be found, presumably due to the unfavorable Pauli repulsion between the electron-rich (L3)Ir and the chlorine atom in PhCl. Because charge transfer from PhCl to Ir occurs before charge transfer from Ir to PhCl, because the charge transfer from PhCl to (L3)Ir is less stabilizing than that to (L1)Ir^+^, and because the charge transfer from (L3)Ir to PhCl is more stabilizing than that from (L1)Ir^+^, the transition states for the neutral (L3)Ir are earlier than those for the cationic (L1)Ir. We further hypothesize that the significantly less stabilizing interaction energy in (L3)Ir-H-TS than that in (L1)Ir-H-TS can be attributed to two factors: (1) the interactions between a C–H bond that is prone to donate electron density and the less electrophilic, neutral (L3)Ir complex are less stabilizing than those between the same C–H bond and the more electrophilic, cationic (L1)Ir complex; (2) (L3)Ir-H-TS is an earlier transition state than (L1)Ir-H-TS, and the orbitals of the approaching Ir and PhCl fragments in (L3)Ir-H-TS overlap less than those in (L1)Ir-H-TS do.

**Fig. 6:**
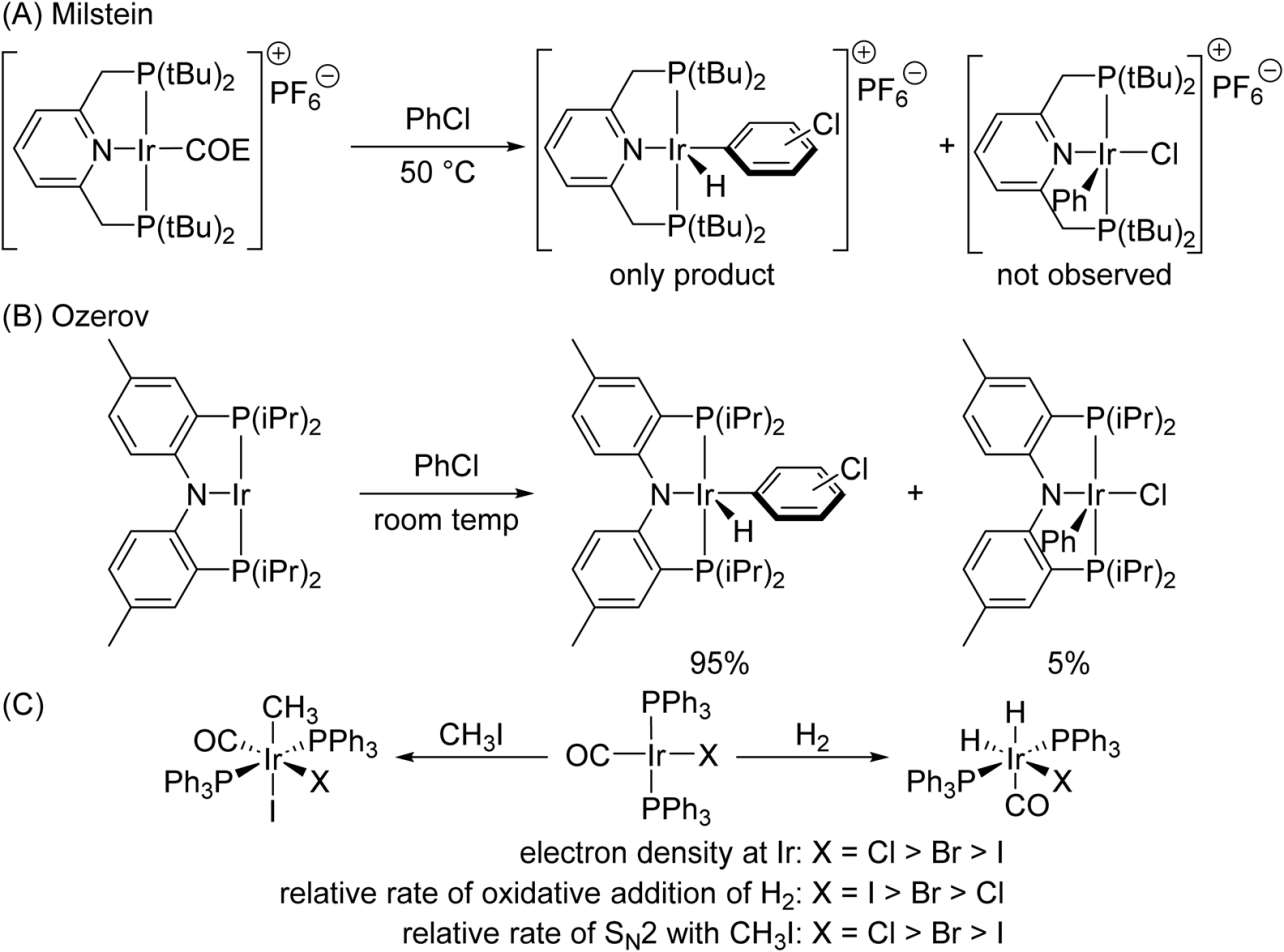
(A) Oxidative additions with a cationic (PNP)Ir complex (COE = cyclooctene); (B) oxidative additions with a neutral (PNP)Ir complex; (C) trends in relative rates of reactions with Vaska-type complexes.

In summary, we propose that orbital interactions between (L1)Ir and a C–H bond are more stabilizing than those between (L1)Ir and a C–Cl bond because the (L1)Ir complex is electrophilic and a C–H bond is more prone to donate electron density to Ir than is a C–Cl bond, presumably due to the high electronegativity of chlorine. The interactions between two electrophilic fragments, *i.e.*, the cationic Ir(i) complex and the C–Cl bond, significantly destabilize the transition state for oxidative addition of the C–Cl bond, even though oxidative addition of the C–Cl bond is thermodynamically more favorable than that of the C–H bond. Consistent with our explanation and data from experiments and calculations, the neutral (L3)Ir complex, which is less electrophilic (less electron-poor) than (L1)Ir^+^, is less selective for the oxidation addition of C–H bonds over C–Cl bonds than is (L1)Ir^+^ because orbital interactions in the transition state between the C–H bond and the less electron-poor Ir complex are less stabilizing than those with (L1)Ir^+^, which is highly electrophilic. Indeed, reaction of PhCl with a cationic (PNP)Ir complex containing an L-type N donor formed the products solely from oxidative addition of the C–H bonds (Fig. 6(A))^46^ whereas reaction of PhCl with a more electron-rich, neutral (PNP)Ir complex containing an X-type N donor formed a mixture of products from oxidative addition of the C–H and C–Cl bonds (Fig. 6(B)).^58^

Our explanation for the high selectivity of an electrophilic Ir complex to oxidatively add C–H bonds over C–Cl bonds is also consistent with the trend in reactivities of Vaska-type complexes.^1^ As shown in Fig. 6(C), the complex containing a more electron-rich Ir center reacts faster than the more electron-poor Ir center toward an S_N_2 reaction with methyl iodide because interactions between the electrophilic C–I bond and an electron-rich Ir nucleophile stabilize the transition state. However, the complex containing the more electron-rich Ir center undergoes oxidative addition of hydrogen more slowly than the more electron-poor Ir center because H_2_ is prone to donate electron density, and the interactions between H_2_ and an electron-poor Ir center are more stabilizing than those between H_2_ and an electron-rich Ir center. These results suggest that conclusions from this work are applicable to a broad scope of transition metal complexes from Group 9 containing ligands beyond the PNP class.

#### 2.4.4 Weak Pd–H bond causes the high selectivity of Pd complexes for oxidative addition of C–Cl bonds over C–H bonds

Energy decomposition analysis suggests that the PhCl fragment in transition state Pd-H-TS is significantly more distorted than it is in Pd-Cl-TS (Δ*E*^PhCl^_GD_(Pd-H-TS) = 93.0 kcal mol^−1^, Δ*E*^PhCl^_GD_(Pd-Cl-TS) = 16.2 kcal mol^−1^). The difference in distortion energy (76.8 kcal mol^−1^) between Pd-H-TS and Pd-Cl-TS is greater than the difference in interaction energy between these two structures (Δ*E*_INT_(Pd-H-TS) = −92.6 kcal mol^−1^, Δ*E*_INT_(Pd-Cl-TS) = −30.6 kcal mol^−1^, different by 62 kcal mol^−1^). Therefore, significant destabilization caused by distortion in the transition state Pd-H-TS is a major contributor to the high barrier to oxidative addition of C–H bonds to Pd species. We recognize that this conclusion offers limited insight into the high selectivity of Pd complexes to oxidatively add C–Cl bonds over C–H bonds because this difference in distortion energy results from the endergonicity of the oxidative addition of C–H bonds to Pd species (*vide supra*). In agreement with Hammond's postulate, which states that the transition state of an endergonic reaction is more similar in structure to the product than to the reactant, the structure of Pd-H-TS is similar to that of the product Pd(H)(Ar), and the C–H bond is broken in the transition state (bond length = 1.951 Å, Table 4, entry 4). Such a long distance between the carbon and the hydrogen atom naturally leads to large distortion energy. In this case, the conclusion derived from EDA also can be derived from conventional analysis of structures and thermodynamics. Therefore, the high selectivity of Pd complexes to undergo oxidative addition of C–Cl bonds over C–H bonds is directly related to the endergonicity of oxidative addition of C–H bonds *versus* the exergonicity of the addition of C–Cl bonds. We propose that such endergonicity stems primarily from the well-established, low BDE of Pd–H bonds^64–66^ and the trend that late transition metals form weaker M–H and M–C bonds than do early transition metals.^1^ This conclusion applies to a broad range of palladium complexes because a weak Pd–H bond is an intrinsic property of the metal, regardless of the identity of the ancillary ligand. The weakness of Pd–H and Pd–C bonds facilitates reductive eliminations from Pd to form C–H and C–C bonds that are well established to occur during the many palladium-catalyzed cross-coupling reactions.

To investigate whether the endergonicity of the oxidative addition of C–H bonds to Pd(0) complexes is related to the electronic properties of the metal complexes, we calculated the partial charge of the PhCl fragment based on Atoms-In-Molecules (AIM) or Natural Population Analysis (NPA) for a series of π-complexes. These results are summarized in Table 7. The partial charge of the PhCl fragment in (L2)Pd-SC (entry 3) is more negative than that in Rh, Ir, or Pt complexes containing ligand L1 or L2 (entries 1, 2, 4). This result suggests that (L2)Pd(0) is the most electron-donating complex among the four, and this conclusion is consistent with the high selectivity of the Pd(0) complex to undergo oxidative addition of the more electrophilic C–Cl bonds over the less electrophilic C–H bonds. However, the partial charge of the PhCl fragment in the neutral Ir complex (L3)Ir-SC is more negative than it is in (L2)Pd-SC, (Table 7, entries 3, 5), even though (L3)Ir complexes are selective for oxidative additions of C–H bonds over carbon–halogen bonds.^58^ These results suggest that, in general, a more nucleophilic organometallic complex tends to be selective for oxidative additions of C–Cl bonds over C–H bonds, and a more electrophilic complex tends to be selective for oxidative addition of C–H bonds over C–Cl bonds. This trend is consistent with the lower selectivity of neutral (L3)Ir for addition of C–H bonds over C–Cl bonds than the selectivity of the cationic (L1)Ir. However, the measure of charge on the arene alone does not account for the selectivity in the reactions of C–H *vs.* C–X bonds when comparing Ir to Pd complexes. Such selectivity is influenced by many factors, including the strengths of the M–H and M–C bonds in the products, the electrophilicity and nucleophilicity of the metal complex, and pathways of electron transfer between the arene and the metal center.

**Table 7:** Computed partial charge of the PhCl fragment in π-complexes

Entry	Complex	AIM charge of PhCl (*e*)	NPA charge of PhCl (*e*)
1	(L1)Ir-SC-1	−0.027	−0.036
2	(L1)Rh-SC-1	−0.007	0.010
3	(L2)Pd-SC	−0.138	−0.184
4	(L2)Pt-SC	−0.094	−0.181
5	(L3)Ir-SC	−0.247	−0.254

## 3 Conclusion

We conducted DFT calculations and energy decomposition analysis of iridium, rhodium, palladium, and platinum complexes containing ligands L1, L2 or L3 (L1 = 2,6-bis((dimethylphosphino)methyl)pyridine, L2 = (1-adamantyl)-di-*tert*-butylphosphine, L3 = bis(2-(dimethylphosphino)phenyl)amide) to investigate the origins of the high selectivity of Pd complexes to undergo oxidative addition of C–Cl bonds over C–H bonds, as well as the high selectivity of Ir complexes to oxidative add C–H bonds over C–Cl bonds. Computations suggest that oxidative addition of a C–H bond to the (L2)Pd(0) complex is endergonic, whereas oxidative addition of a C–Cl bond to (L2)Pd(0) is exergonic. Oxidative addition of a C–H bond to the cationic (L1)Ir(i) complex is less exergonic than that of a C–Cl bond, but the barrier to oxidative addition of a C–H bond is lower than that of a C–Cl bond. Consistent with bond dissociation energies of metal–hydrogen, metal–carbon and metal–chlorine bonds for transition metals from groups 9 and 10, the oxidative addition of C–H or C–Cl bonds to third-row metals is more exergonic (or less endergonic) than that to second-row metals. Energy decomposition analysis of the transition states suggests that the barriers to oxidative addition of C–H or C–Cl bonds to second-row transition metals are higher than those to third-row transition metals primarily because the difference between the destabilizing distortion energy of the PhCl fragment in the transition states for the second-row metal is greater than that difference in the stabilizing interaction energy of transition states for the third-row metal. Iridium complexes of the ligand L1 are highly selective towards oxidative addition of C–H bonds because the stabilization afforded by charge transfer between the d orbitals on Ir and the σ and σ* orbitals of the C–H bond is greater than that between Ir and the orbitals of the C–Cl bond. Results from natural population analysis suggest that these iridium(i) complexes are electrophilic, and the comparison between oxidative addition of an aryl C–H bond to the cationic (L1)Ir and the neutral (L3)Ir complex shows that the selectivity of a more electrophilic Ir complex for oxidative addition of a C–H bond over a C–Cl bond is greater than that of a less electrophilic one. During oxidative addition of either a C–H or a C–Cl bond to an Ir complex, charge transfer from the breaking bond to the Ir center occurs before the charge transfer from Ir to the breaking bond. Transition states involving a more electrophilic Ir complex are later than those involving a less electrophilic one because charge transfer from the breaking bond to the more electrophilic Ir center is more stabilizing than it is to the less electrophilic Ir center and because charge transfer from the more electrophilic Ir center to the breaking bond is less stabilizing than that from the less electrophilic Ir center.

The high selectivity of a palladium(0) complex to undergo oxidative addition of a C–Cl bond over a C–H bond is closely related to the endergonicity of the reaction with a C–H bond, which, in turn, can be attributed primarily to the low bond dissociation energy (BDE) of the Pd–H bond. Analyses based on partial charges suggest that a more electrophilic transition metal complex tends to be more selective for the oxidative addition of C–H bonds over C–Cl bonds and a more nucleophilic complex tends to be selective for the oxidative addition of carbon–halogen bonds over C–H bonds. Yet there are exceptions to this trend, such as neutral iridium(i) complexes containing X-type ligands, and the electronic property is only one of the factors that affect selectivity.

This work demonstrates that energy decomposition analysis, when combined with analyses of thermodynamics, reaction barriers, and partial charges, is a powerful tool to understand the origins of the selectivities of transition metal complexes. As demonstrated by the example of the neutral ligand L1*versus* the anionic ligand L3, the properties of the ligand affect the reactivity and selectivity of metal complexes. Nevertheless, the conclusions from this work are transferable to a broad range of complexes of Group 9 and 10 metals because many of our conclusions are based on the intrinsic properties of the metals, such as the electrophilicity of iridium, the instability of a Pd–H bond, and the periodic trends of second-row *versus* third-row transition metals, rather than specific properties of the ligands. This study should provide a valuable template for computational studies of the fundamental reactivities of transition-metal complexes with varying oxidation states, overall charges, and ligands.

## Author contributions

J. F. H. and M. H.-G. conceived the project and analyzed the data. Y. Q. and A. J. S. conducted computations, analyzed collected data, and drafted the initial manuscript. K. K. I., A. Z., M. L., and D. H. conducted computations and analyzed collected data. All authors edited or approved the final version of the paper.

## Conflicts of interest

M. H.-G. is a part-owner of Q-Chem Inc, whose software was used for calculations reported here.

## Supplementary Material

SC-OLF-D6SC00090H-s001

## Data Availability

The data supporting this article have been included as part of the supplementary information (SI). Supplementary information: the computational methods, a brief introduction to energy decomposition analysis, discussions on oxidative additions to related Rh and Ir complexes, and the energies and atomic coordinates of all optimized structures. See DOI: https://doi.org/10.1039/d6sc00090h.
