# Supplementary material for: Origins of the selectivity of late transition metals of Group 9 and Group 10 for oxidative addition of C–H vs. C–Cl bonds
Source: Chem Sci. 2026 Apr 8;17(21):10441–55. doi: 10.1039/d6sc00090h (PMC13100908; doi:10.1039/d6sc00090h)
Supplement: SC-017-D6SC00090H-s001 [file SC-017-D6SC00090H-s001.pdf]

Supplementary Information (SI) for Chemical Science.
This journal is © The Royal Society of Chemistry 2026

Supporting Information for

Origins of the Selectivity of Late Transition Metals of Group 9 and Group 10 for Oxidative Addition of C–H vs C–Cl Bonds

Yehao Qiu,[†, 1] Alistair J. Sterling,[†, 1, 2] Kevin Ikeda,[1] Alexander Zech,[1] Matthias Loipersberger,[1] Diptarka Hait,[1] Martin Head-Gordon*[, 1], and John F. Hartwig**[, 1]

[1]Department of Chemistry, University of California, Berkeley, California, 94720, United States
[2]Present address: Department of Chemistry and Biochemistry, The University of Texas at Dallas, Richardson, Texas, 75080, United States

[†]These authors contributed equally to this work.
*mhg@cchem.berkeley.edu
**jhartwig@berkeley.edu

Table of Contents

1. **Computational Methods** 2
2. **Distortion/Interaction Model and Energy Decomposition Analysis** 3
3. **Oxidative Addition of *ortho*- versus *para*-C–H bond in PhCl to (L1)Ir and (L1)Rh Complexes** 4
4. **Oxidative Addition of Aryl C–H and C–Cl Bonds to (tBuL1)Ir and (tBuL1)Rh Complexes** 6
5. **Energies of Optimized Structures** 8
6. **References** 10
7. **Atomic Coordinates of Optimized Structures** 12

1. Computational Methods

All calculations were performed with the Q-Chem (Version 5.4.1) program.[1] Geometry optimizations were conducted with the PBE0 functional[2] and the basis sets def2-SVP for H atoms, def2-SVPD for O, Cl, and P atoms, and def2-TZVP (with matching Karlsruhe effective core potential, def2-ECP) for metal centers (**BS1**),[3] with Grimme's D3 dispersion correction with Becke-Johnson damping (D3BJ)[4] in the gas phase. The nature of each stationary point was evaluated by accompanying frequency calculations (all positive eigenvalues for ground states and precisely one negative eigenvalue for transition states). Further single-point energy calculations were performed on the optimized structures with the def2-TZVPD basis set (with def2-ECP for metal centers) for all atoms (**BS2**) using the SMD solvation model with chlorobenzene as the solvent. In all cases, Gibbs free energies for **BS2** were estimated by adding the thermal corrections obtained from frequency calculations using **BS1** to the electronic energies computed at **BS2**.

Energy decomposition analysis was performed using the second-generation absolutely localized molecular orbital energy decomposition analysis (ALMO-EDA).[5] Natural Bond Orbital (NBO) and Natural Population Analysis (NPA) were conducted with the NBO 5.0 program.[6] AIM calculations were conducted with the Multiwfn (Version 3.8) program.[7]

Literature suggests that the PBE0 functional performs well for Pd-mediated bond activation reactions and for calculating barriers to a broad set of transition-metal-mediated insertion and bond activation reactions.[8-11] Nevertheless, computed energy differences on the order of 1–3 kcal/mol lie near the expected uncertainty of DFT methods for these systems and should be interpreted qualitatively in the context of experimental observations and other computational data.

2. Distortion/Interaction Model and Energy Decomposition Analysis

The Activation Strain Model (ASM)[12-14] or the Distortion/Interaction Model[15-16] decomposes the potential energy surface $\Delta E(\zeta)$ along the reaction coordinate ζ of a given chemical reaction into two terms: the reaction strain or distortion energy, $\Delta E_{\text{strain}}(\zeta)$, and the interaction energy, $\Delta E_{\text{int}}(\zeta)$;[17] that is,

$$\Delta E(\zeta) = \Delta E_{\text{strain}}(\zeta) + \Delta E_{\text{int}}(\zeta) \qquad \text{(S1)}$$

The distortion energy, $\Delta E_{\text{strain}}(\zeta)$, refers to the energy required to distort the geometries of the reactants from their lowest-energy ground states to the reactive conformations. By definition, the distortion energy is destabilizing, *i.e.*, $\Delta E_{\text{strain}}(\zeta) > 0$. The interaction energy, $\Delta E_{\text{int}}(\zeta)$, refers to the change in energy caused by interactions between the distorted reactive fragments and is stabilizing, *i.e.*, $\Delta E_{\text{int}}(\zeta) < 0$. Therefore, the sum of $\Delta E_{\text{strain}}(\zeta)$ and $\Delta E_{\text{int}}(\zeta)$ determines the energy profile of a chemical reaction along the reaction coordinate. At the transition state, the potential energy surface reaches a first-order saddle point; that is, for $\zeta = \zeta(\text{TS})$,

$$\frac{\partial \Delta E(\zeta)}{\partial \zeta} = \frac{\partial \Delta E_{strain}(\zeta)}{\partial \zeta} + \frac{\partial \Delta E_{int}(\zeta)}{\partial \zeta} = 0 \qquad (S2)$$

Rearrangement of eq S2 gives the following equation:

$$\frac{\partial \Delta E_{strain}(\zeta)}{\partial \zeta} = -\frac{\partial \Delta E_{int}(\zeta)}{\partial \zeta} \qquad (S3)$$

This equation shows that the rate by which $\Delta E_{\text{strain}}(\zeta)$ increases is equal to the rate by which $\Delta E_{\text{int}}(\zeta)$ decreases at the transition state.

According to Energy Decomposition Analysis based on Absolutely Localized Molecular Orbitals (ALMO EDA),[18-20] the interaction energy, ΔE_{int}, can be further decomposed into three terms: the frozen density term (ΔE_{FRZ}), the polarization term, (ΔE_{POL}), and the charge transfer term, (ΔE_{CT}). The frozen density term (ΔE_{FRZ}) is defined as the change in energy caused by bringing infinitely separated, distorted reactive fragments into the reactive complex without any relaxation of the molecular orbitals (MOs) on those fragments and describes primarily the Pauli repulsion, permanent electrostatic interaction and dispersion of the reactants. The polarization term (ΔE_{POL}) refers to the stabilization energy attributable to relaxation of localized MOs on each reactive fragment in the field produced by all other fragments and quantifies the polarization of electron density that is induced by the approaching reactive fragments. Lastly, the charge transfer term (ΔE_{CT}) describes the stabilization caused by charge transfer from occupied orbitals of one reactive fragment to virtual orbitals of another fragment. ALMO EDA allows the interaction energy (ΔE_{int}) to be interpreted in chemically meaningful terms, such as electrostatic interactions and orbital interactions,[20] and has been used for the analysis of many chemical systems.[18-25]

3. Oxidative Addition of *ortho-* versus *para*-C–H bond in PhCl to (L1)Ir and (L1)Rh Complexes

Consistent with published experimental[26-27] and computational[28-29] studies, our calculations suggest that oxidative addition of the *ortho*-C–H bond in PhCl to (**L1**)Ir and (**L1**)Rh complexes is more favorable than that of the *para*-C–H bond (see Figures S1 and S2) because during the reaction with the *ortho*-C–H bond, the proximal chlorine atom partially coordinates to the metal center and stabilizes the transition state and the product whereas such stabilization is absent in the reaction with the *para*-C–H bond due to the long distance between Cl and the metal center. Although the reaction with the *para*-C–H bond is less energetically favorable than that with the *ortho*-C–H bond, we still chose the former as the model system so that our analysis can focus on the interactions between the metal complex and the C–H bond, without interfering interactions between Cl and the metal center.

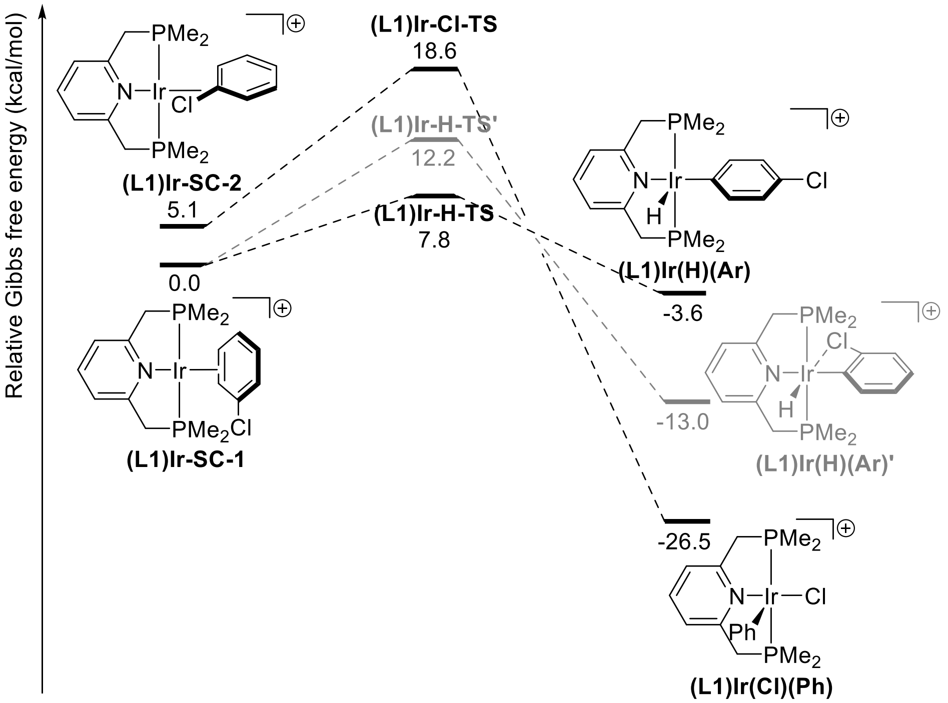

Figure S1. Free-energy diagram of the oxidative addition of the *ortho*- and *para*-C–H bonds as well as the C–Cl bond in PhCl to (**L1**)Ir(I) complexes.

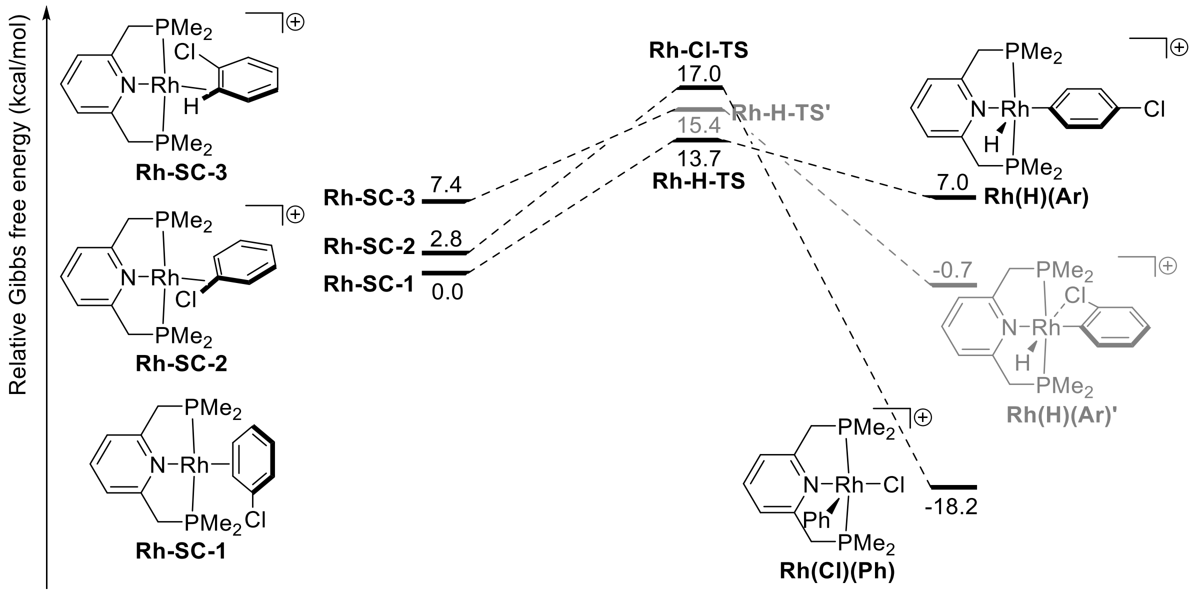

Figure S2. Free-energy diagram of the oxidative addition of the *ortho*- and *para*-C–H bonds as well as the C–Cl bond in PhCl to (**L1**)Rh(I) complexes.

4. Oxidative Addition of Aryl C–H and C–Cl Bonds to (tBuL1)Ir and (tBuL1)Rh Complexes

To investigate the steric effect of the ligand on the selectivity of oxidative addition, we calculated Ir and Rh complexes containing the ligand, 2,6-bis((di-tert-butylphosphino)methyl)pyridine (**tBuL1**), which is more sterically demanding than the ligand **L1** containing methyl groups on phosphorus. The results are shown in Figures S3 and S4. For (**L1**)Rh and (**L1**)Ir complexes, the π-complex between the C(2)–C(3) bond of the chlorobenzene and the metal center (**Ir-SC-1** or **Rh-SC-1**) is the lowest-energy structure prior to the transition state. For (**tBuL1**)Rh and (**tBuL1**)Ir complexes, however, the σ-complex between the C–Cl bond and the metal center (**Ir-SC-2** or **Rh-SC-2**) was found to be the lowest-energy structure prior to the transition state. We propose that steric interactions between PhCl and the *tert*-butyl substituents in **(tBuL1)Ir-SC-1** and **(tBuL1)Rh-SC-1** significantly destabilize these structures, whereas such interactions are negligible in **(tBuL1)Ir-SC-2** and **(tBuL1)Rh-SC-2** because the phenyl ring is positioned far away from the *tert*-butyl groups.

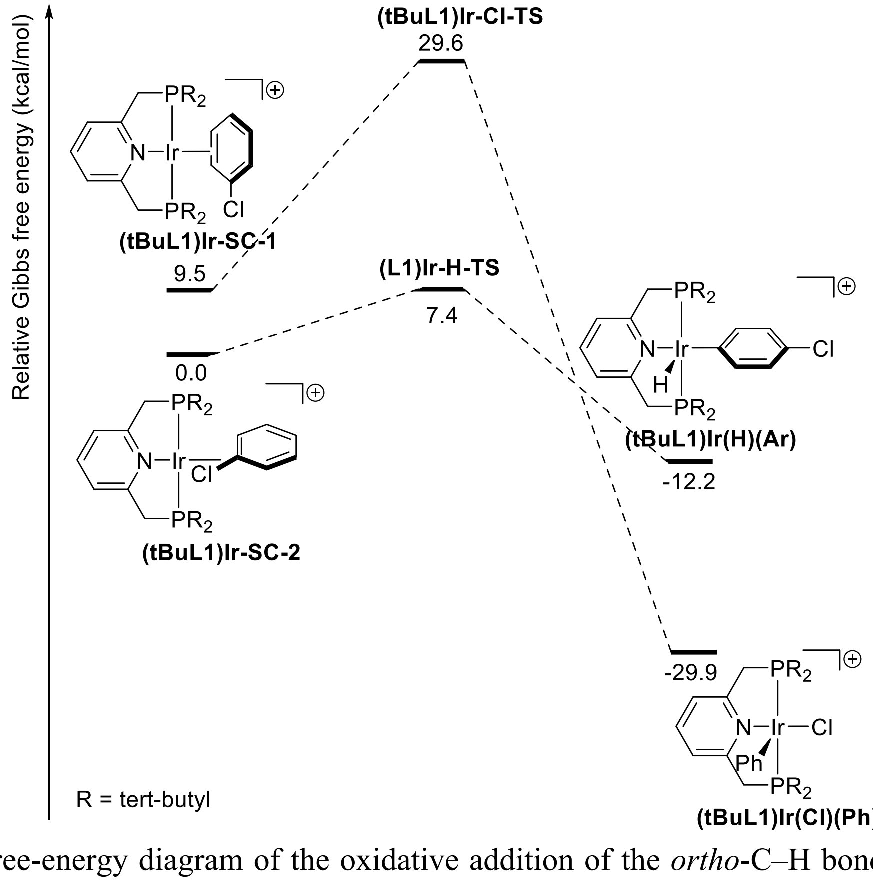

Figure S3. Free-energy diagram of the oxidative addition of the *ortho*-C–H bond and the C–Cl bond in PhCl to (**tBuL1**)Ir(I) complexes.

Oxidative addition of an aryl C–H bond to (**tBuL1**)M^+ (M = Ir or Rh) is thermodynamically more favorable than that to the corresponding (**L1**)M^+ complex, whereas the ΔG value for oxidative addition of an aryl C–Cl bond to (**L1**)M^+ is similar to that for oxidative addition to

($\mathbf{tBuL1}$)M^+. The barrier to oxidative addition of an aryl C–Cl bond to (**tBuL1**)M^+ is significantly higher (by over 10 kcal/mol) than that to the corresponding (**L1**)M^+ complex, whereas the barrier to oxidative addition of an aryl C–H bond to (**L1**)M^+ is similar to that for addition to (**tBuL1**)M^+. These computed data suggest that Group 9 metal complexes containing the bulky **tBuL1** ligand are more selective for the oxidative addition of aryl C–H bonds over aryl C–Cl bonds than those containing the **L1** ligand, and that products from C–H activation are the only products that will be formed when PhCl reacts with (**L1**)M^+ or (**tBuL1**)M^+. These conclusions are consistent with reported experimental results.[26, 30]

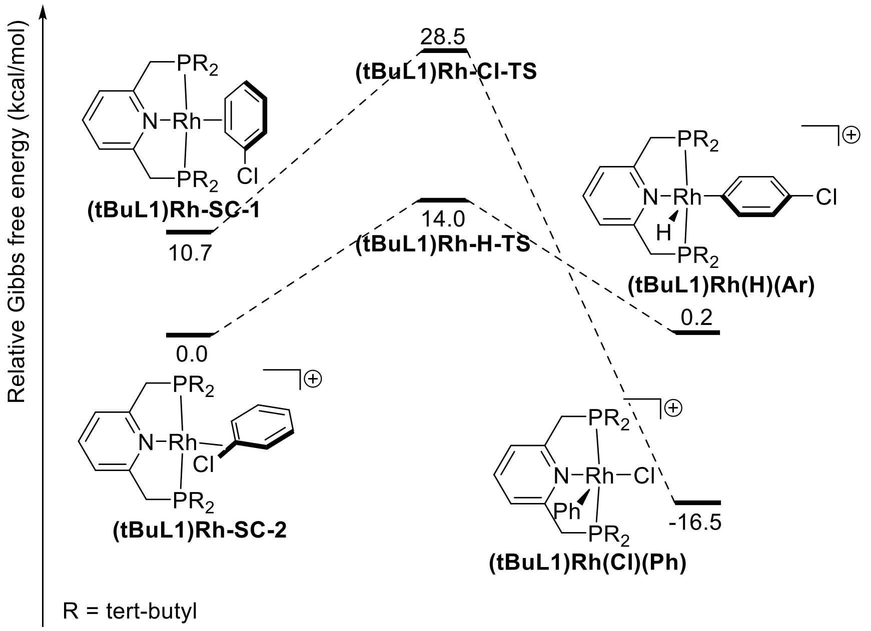

Figure S4. Free-energy diagram of the oxidative addition of the *ortho*-C–H bond and the C–Cl bond in PhCl to (**tBuL1**)Rh(I) complexes.

5. Energies of Optimized Structures

Table S1. Energies of Optimized Structures.

Structure	E(BS1) (Hartree)	Thermal Correction to Enthalpy (kcal/mol)	Thermal Correction to Entropy (cal mol^{-1} K^{-1})	E(BS2) (Hartree)
**PhCl**	-691.129164	61.607	76.062	-691.525282
**(L1)Ir-SC-1**	-1962.181526	251.223	165.752	-1963.391189
**(L1)Ir-SC-2**	-1962.170445	251.463	172.489	-1963.380206
**(L1)Ir-H-TS**	-1962.157335	248.287	171.445	-1963.371382
**(L1)Ir-H-TS'**	-1962.158370	248.478	166.716	-1963.366919
**(L1)Ir-Cl-TS**	-1962.146672	250.201	167.072	-1963.359270
**(L1)Ir(H)(Ar)**	-1962.175185	249.188	172.899	-1963.390307
**(L1)Ir(H)(Ar)'**	-1962.201173	249.517	166.486	-1963.408819
**(L1)Ir(Cl)(Ph)**	-1962.222598	251.358	169.023	-1963.432110
**(tBuL1)Ir-SC-1**	-2433.042523	472.378	216.834	-2434.733940
**(tBuL1)Ir-SC-2**	-2433.051535	471.282	222.380	-2434.744512
**(tBuL1)Ir-H-TS**	-2433.034647	468.982	218.060	-2434.729622
**(tBuL1)Ir-Cl-TS**	-2433.011701	472.080	213.343	-2434.704118
**(tBuL1)Ir(H)(Ar)**	-2433.067677	469.771	219.489	-2434.763988
**(tBuL1)Ir(Cl)(Ph)**	-2433.104305	472.306	217.965	-2434.792742
**(L3)Ir-SC-1**	-2153.151397	278.606	180.774	-2154.518228
**(L3)Ir-H-TS**	-2153.117016	277.339	181.896	-2154.488068
**(L3)Ir-Cl-TS**	-2153.109490	277.727	183.898	-2154.483123
**(L3)Ir(H)(Ar)**	-2153.149517	276.823	188.817	-2154.517776
**(L3)Ir(Cl)Ph)**	-2153.201794	278.987	184.741	-2154.564878
**Rh-SC-1**	-1968.379119	251.145	166.474	-1969.587231
**Rh-SC-2**	-1968.373452	251.398	171.036	-1969.581072
**Rh-SC-3**	-1968.366137	250.749	169.508	-1969.573384
**Rh-H-TS**	-1968.345644	248.103	170.792	-1969.558572
**Rh-H-TS'**	-1968.351146	248.173	167.493	-1969.557481
**Rh-Cl-TS**	-1968.346519	250.075	167.555	-1969.557957
**Rh(H)(Ar)**	-1968.355429	248.863	172.087	-1969.569809
**Rh(H)(Ar)'**	-1968.378926	249.191	166.770	-1969.585106
**Rh(Cl)(Ph)**	-1968.405346	251.238	168.352	-1969.615421
**(tBuL1)Rh-SC-1**	-2439.241827	472.339	216.871	-2440.933043
**(tBuL1)Rh-SC-2**	-2439.254160	471.414	221.053	-2440.945183
**(tBuL1)Rh-H-TS**	-2439.226933	469.189	216.613	-2440.920996
**(tBuL1)Rh-Cl-TS**	-2439.213208	471.750	214.167	-2440.904360
**(tBuL1)Rh(H)(Ar)**	-2439.246840	469.173	220.295	-2440.942206
**(tBuL1)Rh(Cl)(Ph)**	-2439.287183	472.770	215.302	-2440.976254
**Pd-SC**	-1864.760693	379.232	185.012	-1866.018376
**Pd-H-TS**	-1864.712937	376.132	187.779	-1865.974077
**Pd-Cl-TS**	-1864.739286	378.877	180.220	-1865.998335
**Pd(H)(Ar)**	-1864.713222	376.862	189.243	-1865.975306

**Pd(Cl)(Ph)**	-1864.776740	380.118	182.106	-1866.043072
**Pt-SC**	-1856.208349	379.190	188.052	-1857.468860
**Pt-H-TS**	-1856.178406	376.548	186.891	-1857.439838
**Pt-Cl-TS**	-1856.185409	379.160	181.569	-1857.448425
**Pt(H)(Ar)**	-1856.187422	377.478	190.508	-1857.450664
**Pt(Cl)(Ph)**	-1856.245618	380.247	182.265	-1857.511305

6. References

[1] E. Epifanovsky, A. T. B. Gilbert, X. Feng, J. Lee, Y. Mao, N. Mardirossian, P. Pokhilko, A. F. White, M. P. Coons, A. L. Dempwolff, Z. Gan, D. Hait, P. R. Horn, L. D. Jacobson, I. Kaliman, J. Kussmann, A. W. Lange, K. U. Lao, D. S. Levine, J. Liu, S. C. McKenzie, A. F. Morrison, K. D. Nanda, F. Plasser, D. R. Rehn, M. L. Vidal, Z.-Q. You, Y. Zhu, B. Alam, B. J. Albrecht, A. Aldossary, E. Alguire, J. H. Andersen, V. Athavale, D. Barton, K. Begam, A. Behn, N. Bellonzi, Y. A. Bernard, E. J. Berquist, H. G. A. Burton, A. Carreras, K. Carter-Fenk, R. Chakraborty, A. D. Chien, K. D. Closser, V. Cofer-Shabica, S. Dasgupta, M. d. Wergifosse, J. Deng, M. Diedenhofen, H. Do, S. Ehlert, P.-T. Fang, S. Fatehi, Q. Feng, T. Friedhoff, J. Gayvert, Q. Ge, G. Gidofalvi, M. Goldey, J. Gomes, C. E. González-Espinoza, S. Gulania, A. O. Gunina, M. W. D. Hanson-Heine, P. H. P. Harbach, A. Hauser, M. F. Herbst, M. H. Vera, M. Hodecker, Z. C. Holden, S. Houck, X. Huang, K. Hui, B. C. Huynh, M. Ivanov, Á. Jász, H. Ji, H. Jiang, B. Kaduk, S. Kähler, K. Khistyaev, J. Kim, G. Kis, P. Klunzinger, Z. Koczor-Benda, J. H. Koh, D. Kosenkov, L. Koulias, T. Kowalczyk, C. M. Krauter, K. Kue, A. Kunitsa, T. Kus, I. Ladjánszki, A. Landau, K. V. Lawler, D. Lefrancois, S. Lehtola, et al., *J. Chem. Phys.* 2021, **155**, 084801.

[2] M. Ernzerhof, G. E. Scuseria, *J. Chem. Phys.* 1999, **110**, 5029-5036.

[3] F. Weigend, R. Ahlrichs, *Phys. Chem. Chem. Phys.* 2005, **7**, 3297-3305.

[4] S. Grimme, S. Ehrlich, L. Goerigk, *J. Comput. Chem.* 2011, **32**, 1456-1465.

[5] P. R. Horn, Y. Mao, M. Head-Gordon, *Phys. Chem. Chem. Phys.* 2016, **18**, 23067-23079.

[6] E. D. B. Glendening, J. K.; Reed, A. E.; Carpenter, J. E.; Bohmann, J. A.; Morales, C. M.; Weinhold F., Theoretical Chemistry Institute, University of Wisconsin, Madison, 2001.

[7] T. Lu, F. Chen, *J. Comput. Chem.* 2012, **33**, 580-592.

[8] K. H. Hopmann, *Organometallics* 2016, **35**, 3795-3807.

[9] M. M. Quintal, A. Karton, M. A. Iron, A. D. Boese, J. M. L. Martin, *J. Phys. Chem. A* 2006, **110**, 709-716.

[10] M. Steinmetz, S. Grimme, *ChemistryOpen* 2013, **2**, 115-124.

[11] T. Weymuth, E. P. A. Couzijn, P. Chen, M. Reiher, *Journal of Chemical Theory and Computation* 2014, **10**, 3092-3103.

[12] L. P. Wolters, F. M. Bickelhaupt, *WIREs Comput. Mol. Sci.* 2015, **5**, 324-343.

[13] I. Fernández, F. M. Bickelhaupt, *Chem. Soc. Rev.* 2014, **43**, 4953-4967.

[14] F. M. Bickelhaupt, *J. Comput. Chem.* 1999, **20**, 114-128.

[15] D. H. Ess, K. N. Houk, *J. Am. Chem. Soc.* 2007, **129**, 10646-10647.

[16] D. H. Ess, K. N. Houk, *J. Am. Chem. Soc.* 2008, **130**, 10187-10198.

[17] F. M. Bickelhaupt, K. N. Houk, *Angew. Chem. Int. Ed.* 2017, **56**, 10070-10086.

[18] R. Z. Khaliullin, E. A. Cobar, R. C. Lochan, A. T. Bell, M. Head-Gordon, *J. Phys. Chem. A* 2007, **111**, 8753-8765.

[19] Y. Mao, M. Loipersberger, P. R. Horn, A. Das, O. Demerdash, D. S. Levine, S. P. Veccham, T. Head-Gordon, M. Head-Gordon, *Annu. Rev. Phys. Chem.* 2021, **72**, 641-666.

[20] M. v. Hopffgarten, G. Frenking, *WIREs Comput. Mol. Sci.* 2012, **2**, 43-62.

[21] N. I. Saper, A. Ohgi, D. W. Small, K. Semba, Y. Nakao, J. F. Hartwig, *Nat. Chem.* 2020, **12**, 276-283.

[22] Y. Mao, M. Loipersberger, K. J. Kron, J. S. Derrick, C. J. Chang, S. M. Sharada, M. Head-Gordon, *Chem. Sci.* 2021, **12**, 1398-1414.

[23] R. Z. Khaliullin, A. T. Bell, M. Head-Gordon, *Chemistry – A European Journal* 2009, **15**, 851-855.

[24] J. Thirman, E. Engelage, S. M. Huber, M. Head-Gordon, *Phys. Chem. Chem. Phys.* 2018, **20**, 905-915.

[25] P. R. Horn, E. J. Sundstrom, T. A. Baker, M. Head-Gordon, *J. Chem. Phys.* 2013, **138**, 134119.

[26] E. Ben-Ari, M. Gandelman, H. Rozenberg, L. J. W. Shimon, D. Milstein, *J. Am. Chem. Soc.* 2003, **125**, 4714-4715.

[27] L. Fan, S. Parkin, O. V. Ozerov, *J. Am. Chem. Soc.* 2005, **127**, 16772-16773.

[28] H. Wu, M. B. Hall, *Can. J. Chem.* 2009, **87**, 1460-1469.

[29] H. Wu, M. B. Hall, *Dalton Trans.* 2009, 5933-5942.
[30] E. Ben-Ari, R. Cohen, M. Gandelman, L. J. W. Shimon, J. M. L. Martin, D. Milstein, *Organometallics* 2006, **25**, 3190-3210.

7. Atomic Coordinates of Optimized Structures

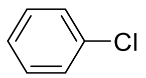

12
PhCl

H	-2.1061947027	-2.1572311959	0.0025503860
C	-1.5656771096	-1.2078321649	0.0015922233
C	-0.1722221293	-1.2150088364	-0.0020815730
H	0.3874967102	-2.1515391852	-0.0039374734
C	0.5093504087	0.0001702929	-0.0032438303
Cl	2.2480910851	0.0022044978	-0.0079395517
C	-0.1747642572	1.2140271194	-0.0010278469
H	0.3828897468	2.1517832857	-0.0022376803
C	-1.5681473663	1.2039547160	0.0025319143
H	-2.1107184647	2.1521801908	0.0043845697
C	-2.2665996069	-0.0027020663	0.0038499480
H	-3.3586529638	-0.0037927604	0.0065420621

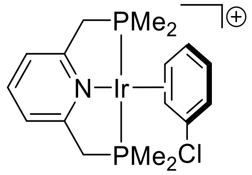

(L1)Ir-SC-1

46
(L1)Ir-SC-1

Ir	-0.00971	-0.20592	0.30374
P	0.69112	2.00314	0.19707
P	-1.27833	-2.09856	0.23783
N	-1.87737	0.71131	0.10427
C	-0.84082	2.77882	0.87328
C	-2.03497	2.03051	0.39093
C	-3.27416	2.65325	0.26191
C	-4.37921	1.91614	-0.14277
C	-4.21262	0.56713	-0.42772
C	-2.95251	-0.00985	-0.31200
C	-2.73969	-1.44748	-0.67247
C	1.97390	2.94386	1.08855
H	1.73265	4.01595	1.03095
H	2.01436	2.62697	2.13834
H	2.95489	2.77972	0.62851
C	0.79344	2.64824	-1.50601
H	-0.07571	2.29442	-2.07776
H	0.82803	3.74758	-1.51985
H	1.69716	2.24342	-1.98393
C	-1.95262	-2.75279	1.80284
H	-2.41367	-1.93392	2.37176
H	-2.69799	-3.54013	1.61603
H	-1.13413	-3.17036	2.40639
C	-0.78977	-3.59641	-0.67926

H	-1.61900	-4.31749	-0.72554
H	0.06039	-4.07545	-0.17200
H	-0.48342	-3.32714	-1.69950
H	-0.76994	2.67960	1.97029
H	-0.92143	3.85100	0.64141
H	-3.36525	3.71399	0.49821
H	-5.36055	2.38616	-0.23581
H	-5.05227	-0.04758	-0.75562
H	-3.65361	-2.04047	-0.51802
H	-2.48196	-1.51478	-1.74374
H	4.30811	0.77382	1.97753
C	3.59482	0.22469	1.35893
C	3.71464	0.29907	-0.05654
H	4.50767	0.89157	-0.51640
C	2.83799	-0.39594	-0.84595
Cl	2.97710	-0.34647	-2.57011
C	1.80803	-1.23176	-0.28963
H	1.48422	-2.06202	-0.92116
C	1.70472	-1.29249	1.13729
H	1.26539	-2.17428	1.61219
C	2.61480	-0.53155	1.94356
H	2.55676	-0.61835	3.03047

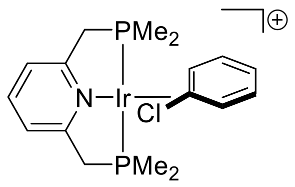

(L1)Ir-SC-2

46

(L1)Ir-SC-2

Ir	0.05050	0.65815	-0.60486
P	0.05242	2.90417	-0.97108
P	0.54469	-1.52288	-0.11657
N	1.96245	0.98420	-0.02433
C	1.86696	3.13060	-1.20236
C	2.60728	2.14291	-0.35452
C	3.91510	2.38482	0.04727
C	4.60793	1.42444	0.77271
C	3.95652	0.24100	1.09144
C	2.63695	0.04577	0.70021
C	1.90183	-1.19995	1.08807
C	-0.71343	3.67205	-2.43499
H	-0.42903	4.73046	-2.52998
H	-0.41020	3.12489	-3.33796
H	-1.80690	3.60645	-2.33935
C	-0.40775	4.01844	0.39884
H	0.11658	3.70602	1.31217
H	-0.15777	5.06445	0.16627
H	-1.48928	3.93763	0.57805
C	1.30265	-2.53028	-1.43638

H	2.10167	-1.95343	-1.92139
H	1.71136	-3.47111	-1.03849
H	0.53923	-2.75854	-2.19348
C	-0.54851	-2.70102	0.74331
H	0.00588	-3.60481	1.03678
H	-1.37130	-2.98672	0.07297
H	-0.98439	-2.22510	1.63149
H	2.05244	2.89617	-2.26511
H	2.21698	4.15804	-1.02449
H	4.38596	3.32735	-0.23408
H	5.64028	1.59485	1.08288
H	4.45772	-0.54261	1.66107
H	2.59048	-2.04788	1.21903
H	1.39621	-1.03284	2.05502
H	-3.98466	-1.82303	-1.72083
C	-3.92757	-1.58586	-0.65704
C	-3.10379	-0.56543	-0.20127
Cl	-2.15275	0.32447	-1.39438
C	-2.99577	-0.22150	1.14032
H	-2.32280	0.58288	1.44346
C	-3.75089	-0.94378	2.06257
H	-3.68779	-0.69166	3.12339
C	-4.58514	-1.97853	1.63745
H	-5.17306	-2.53884	2.36712
C	-4.67409	-2.29630	0.28305
H	-5.32915	-3.10369	-0.05124

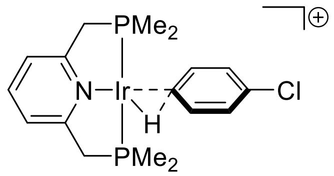

(L1)Ir-H-TS

46

(L1)Ir-H-TS

Ir	0.2894193524	-0.0688930085	-0.3099103199
P	0.1107903806	2.2180999014	-0.4161578292
P	1.1160415190	-2.2140097556	-0.2720662551
N	2.2604494616	0.3743675626	0.2969101364
C	1.9003729121	2.6630173347	-0.4926919268
C	2.7450988270	1.6405982512	0.1986669460
C	4.0095959235	1.9678948853	0.6790321040
C	4.8100973978	0.9789672594	1.2370729532
C	4.3199973002	-0.3188303270	1.3088428163
C	3.0371551203	-0.5972609712	0.8461839077
C	2.4657106222	-1.9770273460	0.9635445233
C	-0.5888846609	3.1631826463	-1.8106630727
H	-0.2244340954	4.2009123585	-1.7757878999
H	-0.2958874201	2.7016333386	-2.7631786963
H	-1.6842396737	3.1822869702	-1.7422169828
C	-0.5445440170	3.0691889672	1.0565365163

H	-0.0267184333	2.7044556086	1.9543872637
H	-0.4294497411	4.1604439704	0.9786142999
H	-1.6110085869	2.8177251963	1.1524377024
C	1.9705436899	-2.6968366305	-1.8099504883
H	2.6160160750	-1.8728183133	-2.1427701880
H	2.5729090415	-3.6054535985	-1.6616766305
H	1.2213815199	-2.8811509948	-2.5927436685
C	0.3016707358	-3.7563779739	0.2592127237
H	1.0081019817	-4.5976120635	0.2024346851
H	-0.5535403258	-3.9638569678	-0.3994361526
H	-0.0627037450	-3.6636424756	1.2912538379
H	2.1612109952	2.6590749768	-1.5658941482
H	2.1060232768	3.6759990479	-0.1156498224
H	4.3638251890	2.9960962060	0.5944050021
H	5.8088153765	1.2170081415	1.6092268431
H	4.9196552257	-1.1228963952	1.7375144565
H	3.2563802391	-2.7399200285	0.8987667946
H	1.9892943145	-2.0860335888	1.9534954455
C	-4.4829480273	-0.4935635779	0.5488587469
C	-3.5581403991	-1.4044156378	1.0593715442
H	-3.8801805929	-2.1566917412	1.7813834099
C	-2.2346383798	-1.3524111674	0.6369168590
C	-1.7816347190	-0.3592543873	-0.2540261643
C	-2.7556808481	0.5061384626	-0.7898734690
H	-2.4726563199	1.2285501222	-1.5561407826
C	-4.0873184673	0.4555459631	-0.3909842453
H	-4.8222519713	1.1424928414	-0.8137583817
H	-0.8618989218	-0.5419575943	-1.3421813275
Cl	-6.1303549055	-0.5616768090	1.0649924278
H	-1.5362048485	-2.0797376635	1.0478190968

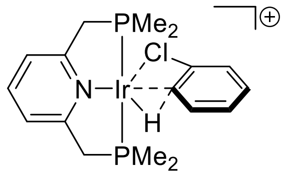

(L1)Ir-H-TS'

46

(L1)Ir-H-TS'

Ir	0.0731194393	0.1583378132	0.1132008161
P	-0.2250197147	-2.1156774593	-0.1858212144
P	0.9362681706	2.2544564152	0.3396088371
N	2.0896693600	-0.3643786107	-0.1004417659
C	1.4443686240	-2.6840235817	0.3556021072
C	2.4818649643	-1.6616346311	0.0094203026
C	3.8142032788	-2.0266490423	-0.1626396237
C	4.7678059584	-1.0495120621	-0.4173276183
C	4.3618777942	0.2759715545	-0.5125811025
C	3.0153265411	0.5962124592	-0.3685816638
C	2.5419931916	2.0091053868	-0.5370043459
C	-1.3771070245	-3.2363565458	0.6601236720

H	-1.1420103519	-4.2785796521	0.3976460514
H	-1.3123317775	-3.0940630997	1.7456141337
H	-2.4029978224	-3.0052360039	0.3435162745
C	-0.3506322141	-2.6091026629	-1.9381953563
H	0.4427759260	-2.1189899822	-2.5192533743
H	-0.2766129300	-3.7002414455	-2.0572825184
H	-1.3199388996	-2.2643205075	-2.3264222788
C	1.3891798252	2.7938763300	2.0208114690
H	1.9592378588	1.9963636069	2.5165718202
H	1.9856223293	3.7179294275	1.9949199301
H	0.4721362716	2.9692983823	2.6006414672
C	0.2021613982	3.7367864837	-0.4200945496
H	0.8594357221	4.6101198221	-0.2971828709
H	-0.7627159279	3.9431875684	0.0648125072
H	0.0205234695	3.5625193488	-1.4891204873
H	1.3845203258	-2.7708088733	1.4547928124
H	1.7157878259	-3.6757956317	-0.0359524075
H	4.0954732947	-3.0770804999	-0.0770367925
H	5.8190130288	-1.3183917431	-0.5407847898
H	5.0813676858	1.0702352954	-0.7158591006
H	3.3186567592	2.7274260200	-0.2341967567
H	2.3332570650	2.1897129403	-1.6059714485
H	-5.5560528085	0.6834516405	-1.8327777570
C	-4.5621196913	0.6428628387	-1.3826731851
C	-3.4669078709	1.2149183215	-2.0309581349
H	-3.5959804963	1.7062179244	-2.9977872226
C	-2.2058529208	1.1702748945	-1.4455568645
H	-1.3587522727	1.6211788633	-1.9666683661
C	-1.9831644055	0.5089934285	-0.2210733687
C	-3.1202833183	-0.0034149388	0.4306253533
Cl	-2.9670909450	-0.6638935100	2.0359931185
C	-4.3920391490	0.0444442524	-0.1392907995
H	-5.2402329773	-0.3710189854	0.4070468344
H	-1.1833130582	0.7791010236	0.9115164795

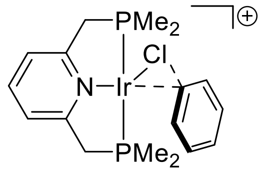

(L1)Ir-Cl-TS

46

(L1)Ir-Cl-TS

Ir	-0.1286887046	-0.0351098356	-0.4871899343
P	0.4174162963	-2.2723668774	-0.6262554990
P	0.1414731467	2.2459504355	-0.4337544435
N	1.6776455094	-0.0043136968	0.4591629858
C	1.3094541914	-2.3575694562	0.9874049176
C	2.1364294332	-1.1174369046	1.1001149509
C	3.3403132991	-1.1027407281	1.7969812945
C	4.0989829148	0.0597993409	1.8444585832

C	3.6506258525	1.1754900451	1.1462365154
C	2.4537425876	1.1176605406	0.4423833057
C	1.9844510435	2.2545013363	-0.4070767394
C	-0.6220437308	-3.7712493329	-0.6813965113
H	0.0090557339	-4.6578135904	-0.8384178895
H	-1.1774654424	-3.8973848995	0.2568054452
H	-1.3381177543	-3.6921357945	-1.5119147211
C	1.6907772833	-2.6140517397	-1.8883627232
H	2.4512074756	-1.8218976890	-1.8637644949
H	2.1661686303	-3.5926475167	-1.7242971843
H	1.2179224503	-2.6016819547	-2.8806587182
C	-0.2605681458	3.2610779617	1.0319715021
H	0.0602502038	2.7297203073	1.9389124741
H	0.2515424274	4.2330818967	0.9733194890
H	-1.3417982910	3.4305789051	1.1013760912
C	-0.3323947026	3.2906206230	-1.8458551063
H	0.0928183204	4.3009822575	-1.7558391098
H	-1.4285419544	3.3669849976	-1.8831416939
H	0.0102771629	2.8213009063	-2.7777933131
H	0.5160595188	-2.3592267059	1.7560340383
H	1.9240915150	-3.2596024696	1.1241293092
H	3.6698095196	-2.0107811992	2.3036554206
H	5.0381812894	0.0906434450	2.4001737330
H	4.2350276125	2.0962170172	1.1225930791
H	2.4162495590	3.2118083143	-0.0812190518
H	2.3079670526	2.0745085703	-1.4470291679
H	-3.2393200458	-2.1701765341	2.7606438009
C	-3.0856390848	-1.2386044144	2.2108751099
C	-2.5380064139	-1.2907808242	0.9317817025
H	-2.3170437982	-2.2434296807	0.4583839598
C	-2.2969611392	-0.0909939839	0.2435610998
Cl	-2.1907986361	-0.1648363791	-1.6612679531
C	-2.7980040620	1.1212209299	0.7480152048
H	-2.7661905052	2.0190099522	0.1301981786
C	-3.3475510237	1.1500584718	2.0231846940
H	-3.7094852037	2.0990234647	2.4260976208
C	-3.4704395577	-0.0220536706	2.7731292295
H	-3.9127698740	0.0075722919	3.7703799828

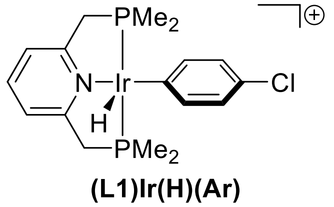

(L1)Ir(H)(Ar)

46

(L1)Ir(H)(Ar)

H	0.4625790293	-0.0545889842	1.5470520180
Ir	0.3494001501	0.0082719182	0.0183443522
P	0.4453661570	2.2961190223	0.1778721883
P	0.8350329766	-2.2366069985	-0.0567472495

N	2.4912948590	0.2123526093	-0.1363719395
C	2.1051675934	2.5854708309	-0.5976589605
C	3.0402837758	1.4263881845	-0.3779597955
C	4.4207182322	1.5797570399	-0.4686715233
C	5.2397922012	0.4637387558	-0.3369633789
C	4.6634087021	-0.7764051693	-0.0891715956
C	3.2794641981	-0.8739433487	0.0298955834
C	2.6241174686	-2.1728226627	0.4170931017
C	-0.7163204181	3.3476406691	-0.7402685136
H	-0.4299468243	4.4082904535	-0.6943370495
H	-0.7649105565	3.0192320668	-1.7875487921
H	-1.7142963057	3.2147215056	-0.2979346879
C	0.5390300967	3.0293526497	1.8404203548
H	1.3247927029	2.5251140026	2.4194198190
H	0.7526854167	4.1067891411	1.7867079956
H	-0.4223238583	2.8784117352	2.3514799408
C	0.7954955127	-3.1023271653	-1.6620486029
H	1.3428514274	-2.5209890683	-2.4172281529
H	1.2470406548	-4.1018137630	-1.5758477449
H	-0.2483288665	-3.2109348711	-1.9891540812
C	0.0514298098	-3.3888690726	1.1057416965
H	0.5102378319	-4.3870862900	1.0608714248
H	-1.0137335498	-3.4617729025	0.8429104826
H	0.1244716631	-2.9874265129	2.1253365418
H	1.9106941279	2.6807698322	-1.6810644868
H	2.5632672101	3.5335046551	-0.2782939622
H	4.8431750604	2.5670653273	-0.6597464345
H	6.3239329457	0.5608939933	-0.4222972821
H	5.2795487892	-1.6683937790	0.0333018544
H	3.1909021195	-3.0357966435	0.0364424800
H	2.6384381969	-2.2483199858	1.5190485085
H	-4.5119913076	0.8367250645	1.7183223527
C	-3.8877439511	0.3747920144	0.9511382879
C	-2.4991626914	0.4386644382	1.0262321691
H	-2.0564783911	0.9624296329	1.8786729436
C	-1.6665309112	-0.1563862685	0.0587157259
C	-2.3154438842	-0.8336479006	-0.9920062309
H	-1.7281376740	-1.3326672016	-1.7713795027
C	-3.7048639091	-0.8884166286	-1.1014140912
H	-4.1825568157	-1.4071526703	-1.9345806357
C	-4.4873415910	-0.2839578576	-0.1221647063
Cl	-6.2155527959	-0.3564515283	-0.2341368956

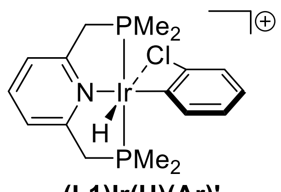

(L1)Ir(H)(Ar)'

46
(L1)Ir(H)(Ar)’

H	-0.2677536641	-0.0525582217	1.8489713440
Ir	-0.0286199203	-0.0372176884	0.3150948296
P	-0.2026042854	-2.3294646291	0.2860529895
P	-0.4719234338	2.2084594725	0.4032737097
N	-2.1210859401	-0.1588080935	-0.0872650357
C	-1.6941054438	-2.4611520091	-0.8011251014
C	-2.6342232538	-1.3118892961	-0.5697268347
C	-3.9892900066	-1.4158123564	-0.8778417139
C	-4.8124808358	-0.3082953357	-0.7150939089
C	-4.2674548284	0.8750543601	-0.2289512078
C	-2.9138516595	0.9216985299	0.0961778458
C	-2.2955870857	2.1424797492	0.7128613800
C	1.0560508955	-3.3882300497	-0.4819630855
H	0.7039308210	-4.4248615265	-0.5829850807
H	1.3188801852	-2.9835131000	-1.4687356623
H	1.9562465573	-3.3675060734	0.1484778026
C	-0.6578720830	-3.1671368065	1.8348302881
H	-1.5233131800	-2.6583224863	2.2812164111
H	-0.9023392752	-4.2244430006	1.6564480490
H	0.1839139493	-3.1017881322	2.5382213278
C	-0.2672327192	3.1958986820	-1.1116734195
H	-0.7862939239	2.7020696117	-1.9446369336
H	-0.6640884961	4.2130896735	-0.9793043969
H	0.8027334718	3.2509449218	-1.3578287150
C	0.2113656031	3.2366436005	1.7335265462
H	-0.2275878177	4.2447970036	1.7334186557
H	1.2980993751	3.3115534715	1.5857253650
H	0.0284088243	2.7487252666	2.7002720034
H	-1.3074806147	-2.4005198652	-1.8342082244
H	-2.2153645660	-3.4261954568	-0.7111442157
H	-4.3864422265	-2.3584662141	-1.2576908349
H	-5.8743788264	-0.3676728538	-0.9636816337
H	-4.8898940279	1.7590292669	-0.0831413284
H	-2.8176568105	3.0580968438	0.3980751877
H	-2.4068318881	2.0692381958	1.8093380928
H	5.0815468791	0.2392230531	1.8350927195
C	4.3295831951	0.1847277581	1.0442805895
C	2.9743124227	0.1394179239	1.3862342194
H	2.6810891797	0.1564415986	2.4391840853
C	1.9917972181	0.0671773677	0.3908889427
C	2.4731526394	0.0483385608	-0.9167802783
Cl	1.1230104578	-0.0437913193	-2.0953983070
C	3.7955006639	0.0901664163	-1.3151888894
H	4.0823320168	0.0710650205	-2.3680540108
C	4.7413517818	0.1603519010	-0.2895159086
H	5.8034337815	0.1963405827	-0.5394104734

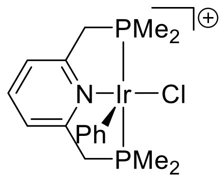

(L1)Ir(Cl)(Ph)

46

(L1)Ir(Cl)(Ph)

Ir	0.3768427002	0.2013618634	0.3009567185
P	-0.8654413731	2.0902487239	0.7776118296
P	1.2796285537	-1.9022716169	0.0057228422
N	-1.2301054460	-0.8216764583	1.0582469298
C	-2.0541705808	1.3212271524	1.9648579505
C	-2.2000911963	-0.1552914627	1.7401358779
C	-3.2993470772	-0.8376182570	2.2554267559
C	-3.3944629494	-2.2151590617	2.1058765670
C	-2.3834265367	-2.8849995647	1.4293290616
C	-1.3163547046	-2.1696196000	0.8950077090
C	-0.2895995660	-2.8617215944	0.0485737863
C	-0.0919451802	3.4606765235	1.6797422095
H	-0.8412761717	4.1887433676	2.0228185400
H	0.4702834327	3.0665518245	2.5371341548
H	0.6226453491	3.9541699006	1.0062327375
C	-1.8559411819	2.8644869607	-0.5319488058
H	-2.4594352248	2.1040798836	-1.0444044545
H	-2.5050256664	3.6459009603	-0.1114529307
H	-1.1728695553	3.3097805120	-1.2690798576
C	2.3120674798	-2.5402891640	1.3588187478
H	1.7794400112	-2.4309403938	2.3141043583
H	2.5614899080	-3.5995951977	1.1992506366
H	3.2372426923	-1.9480913647	1.4047525123
C	2.1254323943	-2.3510635200	-1.5301501238
H	2.3275128499	-3.4309538649	-1.5717530419
H	3.0745500554	-1.7978233916	-1.5709910581
H	1.5060145627	-2.0474451200	-2.3849750061
H	-4.0737346689	-0.2785582540	2.7824730284
H	-4.2496778813	-2.7613826071	2.5094011214
H	-2.4265690950	-3.9653313518	1.2833678526
H	-0.1594142747	-3.9096151581	0.3546792801
H	-0.6565472520	-2.8665048412	-0.9936815721
H	0.5109412149	0.8539359224	-4.7163450899
C	-0.1308906482	0.4933491659	-3.9086392304
C	-1.4079911176	0.0110942099	-4.1883885177
H	-1.7776817418	-0.0141305928	-5.2154165178
C	-2.2070552206	-0.4315457339	-3.1395275774
H	-3.2142814972	-0.8066300494	-3.3379785779
C	-1.7399518697	-0.3980993650	-1.8219853831
H	-2.4146247714	-0.7395356691	-1.0359656058
C	-0.4524234246	0.0766010693	-1.5360980064
C	0.3464969727	0.5279581962	-2.5991296407
H	1.3467280007	0.9144926020	-2.4032156565

H	-3.0383960831	1.8130182086	1.9646077808
H	-1.6262979969	1.4780979358	2.9707686072
Cl	2.3921821180	1.3218310932	0.0405506085

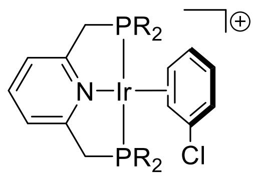

(tBuL1)Ir-SC-1

R = tBu

82

(tBuL1)Ir-SC-1

Ir	-0.1186240283	-0.1977797654	-0.0202883607
P	2.0907655705	0.6612189782	0.1710248995
P	-2.4512081468	-0.2672196056	-0.0781983369
N	-0.5327487563	1.8117433703	-0.4950840708
C	1.5702885657	2.4034802428	0.5380678102
C	0.3480037675	2.7953357264	-0.1992528063
C	0.0976544813	4.1281650128	-0.5202684596
C	-1.0949791789	4.4737900627	-1.1382359388
C	-2.0139340984	3.4671891640	-1.4103505868
C	-1.7119488829	2.1503433052	-1.0809911009
C	-2.6737100454	1.0510880567	-1.3521030770
C	3.2115278110	0.4041963976	1.6911661717
C	3.1098104889	0.8687310899	-1.4174607735
C	-3.1753152946	0.4504605716	1.5323067917
C	-3.5606316537	-1.6483857428	-0.7743088001
H	1.3278577283	2.4028961519	1.6136316834
H	2.3753504355	3.1341815657	0.3906247532
H	0.8418688735	4.8837200126	-0.2655908921
H	-1.3115782367	5.5125795891	-1.3956151344
H	-2.9703936290	3.6897263793	-1.8849363155
H	-3.7006862252	1.4278439218	-1.4484447664
H	-2.3979822082	0.5701828611	-2.3029735925
H	3.0040659197	-3.8396517829	1.3498495253
C	2.1802056062	-3.3578639671	0.8189177533
C	2.2038919035	-3.3310322293	-0.6031293465
H	3.0404991398	-3.7662401910	-1.1520351292
C	1.1365910945	-2.8087635427	-1.2774027084
Cl	1.0600357694	-2.8747276297	-3.0068921251
C	-0.0100488132	-2.2780997552	-0.6008521544
H	-0.9387627266	-2.3882692903	-1.1545110072
C	0.0003547359	-2.2668414978	0.8306112697
H	-0.9422743066	-2.2979893960	1.3817134637
C	1.1235834308	-2.8360762665	1.5148222282
H	1.0842902921	-2.9386725092	2.6013085768
C	-4.4070099232	1.3262958193	1.2995805709
H	-4.7501838853	1.7182026815	2.2704533637
H	-4.1753232786	2.1979000742	0.6700099984
H	-5.2483984031	0.7869521552	0.8503184249

C	-2.0933190479	1.3165679277	2.1928940835
H	-1.8820036414	2.2283472072	1.6188745752
H	-2.4534622687	1.6285007906	3.1859977500
H	-1.1486070896	0.7628683870	2.3154454607
C	-3.5029069337	-0.6935963567	2.4945202255
H	-4.3435898663	-1.3114877165	2.1549171188
H	-2.6334318460	-1.3463495783	2.6665837496
H	-3.7858153587	-0.2674286455	3.4697497728
C	-5.0442360214	-1.2793959788	-0.6934949952
H	-5.4212025373	-1.2651321250	0.3369105256
H	-5.2657054843	-0.3092782470	-1.1611238450
H	-5.6231239567	-2.0415980867	-1.2387284792
C	-3.2120112956	-1.8378801474	-2.2602012904
H	-3.6661713311	-2.7783065137	-2.6085260648
H	-3.6267172428	-1.0337056793	-2.8837369025
H	-2.1339946742	-1.9006421554	-2.4678809281
C	-3.3391772021	-2.9717492177	-0.0301738032
H	-4.0466013870	-3.7164279663	-0.4275720312
H	-2.3312211292	-3.3794127563	-0.1715564343
H	-3.5260134029	-2.8910401955	1.0471024802
C	2.2808857333	0.0525042272	2.8551548527
H	1.5255890363	0.8330644945	3.0376608454
H	1.7498233078	-0.8883939656	2.6848321965
H	2.8773562560	-0.0454620318	3.7762024821
C	3.9967106442	1.6626349854	2.0854886302
H	4.6406252958	2.0448963337	1.2853206804
H	3.3516471369	2.4789920213	2.4363152086
H	4.6525536635	1.3972745508	2.9297469534
C	4.2118393328	-0.7253836281	1.4528469368
H	3.7276849901	-1.6352575373	1.0824960336
H	4.9945533274	-0.4293074534	0.7415930039
H	4.7096464513	-0.9727607555	2.4036835059
C	3.6235111617	-0.4826490909	-1.9152227233
H	2.8036602618	-1.1322538666	-2.2383610849
H	4.2599974681	-0.3078133350	-2.7972445050
H	4.2291301249	-1.0180157316	-1.1741518290
C	2.1452736692	1.4231298450	-2.4727318296
H	1.2413814648	0.8003613826	-2.5572962143
H	1.8408755190	2.4592123223	-2.2649123694
H	2.6516881135	1.4234111951	-3.4507880442
C	4.2932833106	1.8231965102	-1.2611856682
H	4.7293811915	2.0035790380	-2.2565851042
H	4.0112131851	2.8047184166	-0.8542295975
H	5.0899872555	1.4007729048	-0.6347109139

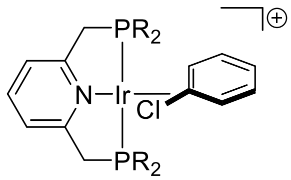

(tBuL1)Ir-SC-2

R = tBu

82

(tBuL1)Ir-SC-2

Ir	-0.0232229702	-0.1736654247	0.0828599307
P	-2.2133171826	0.5723871495	0.1108324103
P	1.9242243583	-1.4308966303	0.0923006597
N	-0.8301209809	-1.8864798620	-0.6453623275
C	-3.0179746993	-1.0894403000	0.1138718240
C	-2.1730676809	-2.1049336700	-0.5782671270
C	-2.7492652765	-3.2663726730	-1.0842833799
C	-1.9493704794	-4.2549143649	-1.6404073572
C	-0.5770711867	-4.0479000991	-1.6709616059
C	-0.0403912184	-2.8623688347	-1.1785218855
C	1.4283845064	-2.6150968504	-1.2375253395
C	-2.8924472135	1.3457351672	1.6967397046
C	-2.8663523064	1.4080253171	-1.4486894851
C	2.0669300239	-2.4823563032	1.6648904133
C	3.6085004842	-0.7993359917	-0.4979651503
H	-3.0808524646	-1.3767964203	1.1764953088
H	-4.0448898942	-1.0927277697	-0.2781579785
H	-3.8309296659	-3.3901775618	-1.0163576143
H	-2.3866884335	-5.1744420658	-2.0341103361
H	0.0964379328	-4.7981428657	-2.0872619335
H	1.9899184137	-3.5592432901	-1.2475753228
H	1.6501243578	-2.0916120765	-2.1796594214
H	1.9288501356	4.3816910962	1.5112046709
C	1.9294562025	4.1747426545	0.4395136674
C	1.4424665620	2.9716698228	-0.0555408273
Cl	0.8359708708	1.7839521839	1.1016849952
C	1.4253030527	2.6611195335	-1.4076469329
H	1.0185089636	1.6931888935	-1.7163010448
C	1.9235549553	3.6033710363	-2.3057036931
H	1.9203184730	3.3817980624	-3.3752470131
C	2.4201340721	4.8212281949	-1.8423620768
H	2.8086790037	5.5562959919	-2.5499826672
C	2.4213257886	5.1041560004	-0.4761341181
H	2.8099544847	6.0581014143	-0.1134423987
C	3.4499905072	-0.3810395192	-1.9653263825
H	4.3092404272	0.2446434512	-2.2523633854
H	3.4388241774	-1.2437723766	-2.6465481809
H	2.5392779946	0.2113655199	-2.1339107253
C	4.7149071650	-1.8493480817	-0.4059257563
H	5.6195174794	-1.4546430359	-0.8955640562
H	4.9860035055	-2.0806287826	0.6327167326
H	4.4508849330	-2.7867749047	-0.9178960777

C	2.7581834281	-1.6461061114	2.7436891334
H	3.8268323155	-1.4975895718	2.5381612975
H	2.2826420273	-0.6591133177	2.8571596443
H	2.6806942224	-2.1667910379	3.7111846613
C	2.7878576381	-3.8151437285	1.4690733181
H	3.8351235806	-3.7048545549	1.1674060298
H	2.7787558918	-4.3650518041	2.4237900565
H	2.2783742047	-4.4529767361	0.7320971656
C	0.6335754827	-2.7763566737	2.1259570288
H	0.6716198447	-3.3016709701	3.0934891044
H	0.0535935733	-1.8475611643	2.2403630675
H	0.0956760056	-3.4261125933	1.4199732108
C	-2.1261269853	0.6933638117	2.8538055333
H	-1.0504500165	0.9076069191	2.8130343991
H	-2.5178826146	1.0812995928	3.8075032933
H	-2.2439528585	-0.4005352794	2.8725322376
C	-2.6328572405	2.8540631759	1.7070864696
H	-1.5835300674	3.0996874095	1.4889828297
H	-3.2717195783	3.3870053474	0.9899222086
H	-2.8671567209	3.2504637361	2.7076655021
C	-4.3876448499	1.0813925263	1.8948300248
H	-4.7042632879	1.5429250267	2.8437148177
H	-5.0067711043	1.5125419427	1.0999831401
H	-4.6202158963	0.0096178045	1.9693852117
C	-2.1325291771	2.7342892086	-1.6562660412
H	-1.0440783880	2.5978709505	-1.6570518985
H	-2.4197337314	3.1563263731	-2.6323853567
H	-2.3793131149	3.4792759990	-0.8901282329
C	-4.3746790913	1.6440400359	-1.4474952440
H	-4.6724030884	2.4187225661	-0.7282139280
H	-4.6826213487	1.9952212750	-2.4453203869
H	-4.9499907167	0.7307704016	-1.2347186946
C	-2.4947158541	0.4628297716	-2.5978153039
H	-1.4179467622	0.2322052955	-2.5938517830
H	-3.0504145791	-0.4858228512	-2.5604683000
H	-2.7412075426	0.9477251982	-3.5554503739
C	3.9999332642	0.4389332891	0.3142029722
H	4.9884932642	0.7871528617	-0.0243061232
H	3.2926013958	1.2617777946	0.1605468030
H	4.0703029355	0.2428090428	1.3909786783

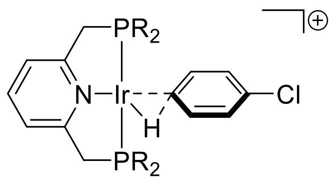

(tBuL1)Ir-H-TS

R = tBu

82

(tBuL1)Ir-H-TS

Ir	0.2497410792	0.0631522163	-0.0584150591

P	-1.1997413183	1.8998517227	-0.1869240138
P	2.1696584590	-1.2388239975	-0.0305876890
N	1.5893245715	1.4987351120	0.6880417378
C	0.0921751520	3.2240413707	-0.1632795301
C	1.3086391397	2.8210973000	0.5966944744
C	2.1602489269	3.7827983923	1.1356702845
C	3.3411964757	3.3880310714	1.7490465478
C	3.6437573702	2.0330416439	1.8035798891
C	2.7515286131	1.1060655401	1.2731196108
C	3.0440099528	-0.3570472541	1.3398306194
C	-2.1136719726	2.3106348902	-1.7931232769
C	-2.2380520883	2.2450807219	1.3492285929
C	3.2264381494	-0.9239661686	-1.5761329274
C	2.1953387263	-3.0563065557	0.4854636858
H	-0.2871373285	4.1971632613	0.1779856451
H	1.8948102421	4.8373428662	1.0509064480
H	4.0239927704	4.1282774946	2.1713886198
H	4.5666716794	1.6805130300	2.2656276143
H	4.1260765395	-0.5429385187	1.3819491696
H	2.6093160246	-0.7567070073	2.2687385074
C	-3.6859523026	-2.8739308570	0.1269739012
C	-2.7620310004	-2.7294173522	1.1638211797
H	-2.9499959039	-3.2041321979	2.1283774533
C	-1.6094108948	-1.9812849354	0.9591247820
C	-1.3650993963	-1.3155729788	-0.2595974048
C	-2.2978820590	-1.5151477281	-1.2899642410
H	-2.1244219873	-1.0744160412	-2.2733394653
C	-3.4501194463	-2.2789205318	-1.1072722254
H	-4.1605183349	-2.4156518600	-1.9242067526
H	-0.2432718163	-1.1112253829	-1.0490762485
Cl	-5.1180426071	-3.8099878207	0.3772104823
H	-0.9104623490	-1.8727781956	1.7889688098
C	2.9671607552	0.5182672806	-2.0287839404
H	3.4863940021	0.6855104590	-2.9856147721
H	1.8911736095	0.7065794115	-2.1645867368
H	3.3564100115	1.2552875584	-1.3120003614
C	2.7597514784	-1.8649129662	-2.6887437001
H	3.0012469768	-2.9155328693	-2.4815456556
H	1.6760006565	-1.7814564677	-2.8636625647
H	3.2681222785	-1.5912722222	-3.6265472846
C	4.7274124434	-1.0829340581	-1.3297345212
H	5.0149657409	-2.0932705372	-1.0196581259
H	5.2626823655	-0.8632858954	-2.2671406173
H	5.0979460864	-0.3695222217	-0.5790617079
C	3.5698813185	-3.7127515746	0.3550910815
H	3.5170399001	-4.7250070922	0.7861601192
H	3.8873725586	-3.8238065881	-0.6900286969
H	4.3506531349	-3.1661922976	0.9047153902
C	1.7684796326	-3.1214296971	1.9568697055
H	1.5098482591	-4.1620245557	2.2059773983
H	2.5761328004	-2.8176354416	2.6380357827
H	0.8845587035	-2.5037182822	2.1653111950

C	1.1691861799	-3.8185785891	-0.3610428704
H	1.1751520755	-4.8766819922	-0.0556048514
H	0.1508634871	-3.4376350506	-0.2129579801
H	1.3956700891	-3.7871938753	-1.4339106300
H	0.3923466343	3.3526806778	-1.2165933286
C	-1.2641618929	1.7244737295	-2.9258250267
H	-1.1781310345	0.6327612952	-2.8595637138
H	-1.7251313714	1.9786367010	-3.8933924952
H	-0.2408669033	2.1304783227	-2.9317308097
C	-3.5085125860	1.6825006170	-1.7999476903
H	-3.4973321207	0.6219716513	-1.5203166070
H	-4.1948532610	2.2089225505	-1.1229462663
H	-3.9304602143	1.7603039586	-2.8143627232
C	-2.2502094192	3.8205720533	-2.0201435519
H	-2.8251619784	3.9805111722	-2.9459143107
H	-2.7888576083	4.3282019984	-1.2109301993
H	-1.2815348663	4.3197303922	-2.1591410382
C	-3.2697226677	1.1293107714	1.5370920944
H	-2.7985852074	0.1398273717	1.5891301190
H	-3.8038633458	1.2986274385	2.4854133040
H	-4.0202836629	1.1056934662	0.7381416847
C	-2.9379527053	3.6021451966	1.3242401885
H	-3.7284184713	3.6464940461	0.5634929923
H	-3.4177072353	3.7745535626	2.3007367795
H	-2.2433607279	4.4381956407	1.1540470555
C	-1.2531032286	2.1974973408	2.5238701415
H	-0.6863039434	1.2531869797	2.5357573535
H	-0.5366140393	3.0321463305	2.5089964461
H	-1.8165874328	2.2682042567	3.4673062739

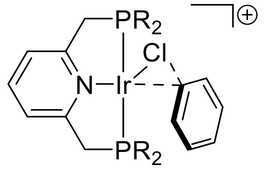

(tBuL1)Ir-Cl-TS

R = tBu

82

(tBuL1)Ir-Cl-TS

Ir	0.0660105518	0.1169787279	0.1939100569
P	2.4070004688	-0.1261481114	0.0405402287
P	-2.1809494373	-0.5409172639	0.3539411925
N	0.1703944592	-1.5505431716	-0.9969336440
C	2.3975419707	-0.8457325892	-1.6662986126
C	1.2573516695	-1.7934998948	-1.7782606717
C	1.3244771848	-2.9097761245	-2.6072798896
C	0.2795740883	-3.8239811775	-2.6247714122
C	-0.7807539237	-3.6260385930	-1.7485951105
C	-0.7978168990	-2.5058821178	-0.9253693918
C	-1.8131924730	-2.3375632925	0.1441843783
C	3.8015651925	1.1772501637	-0.0729409264

C	2.9304340170	-1.5191339493	1.2354438244
C	-3.4017164275	-0.2389611666	-1.0940431167
C	-3.0377622727	-0.5124030539	2.0386002095
H	2.2240917683	0.0156763055	-2.3295417035
H	3.3402869109	-1.3212432003	-1.9710905006
H	2.2079379612	-3.0506282217	-3.2312686403
H	0.3087796410	-4.6955694134	-3.2814855608
H	-1.5909685089	-4.3521961671	-1.6706218595
H	-2.6914946087	-2.9711844089	-0.0188369255
H	-1.3439396348	-2.6537824089	1.0888297974
H	0.8950377322	4.2108263222	-2.6117688760
C	0.1407882657	3.8092229961	-1.9306930281
C	0.5465201686	2.9743254061	-0.8926840757
H	1.5956517657	2.7674501490	-0.7288596100
C	-0.4122928047	2.4079683331	-0.0475390986
Cl	0.1070053100	1.9164594370	1.7372374908
C	-1.7445912470	2.8384353338	-0.1230120068
H	-2.4571653132	2.5109746291	0.6297444621
C	-2.1307481687	3.6907702512	-1.1497170863
H	-3.1755222974	4.0041076858	-1.2151451795
C	-1.1997015905	4.1609754426	-2.0779708580
H	-1.5104153067	4.8331444439	-2.8795967800
C	-4.4410676461	0.8403013210	-0.7841681795
H	-3.9947420679	1.7936239622	-0.4874355839
H	-5.0263853903	1.0295023929	-1.6978319170
H	-5.1499897679	0.5261602973	-0.0064301644
C	-4.1612488350	-1.5146688957	-1.4773418235
H	-4.7274217119	-1.9506300502	-0.6424845170
H	-4.8891489992	-1.2577013173	-2.2628225317
H	-3.5017897770	-2.2847528679	-1.9006799145
C	-2.5501037200	0.1907722521	-2.2948505017
H	-1.9910602446	1.1139159372	-2.1008000435
H	-1.8299309254	-0.5875645845	-2.5842652918
H	-3.2131624255	0.3633272108	-3.1577711126
C	-3.3933633495	0.9210048169	2.4431886226
H	-2.4956812377	1.5486173616	2.5273428731
H	-4.1029329335	1.3985971436	1.7563629590
H	-3.8692366989	0.8989027595	3.4361180556
C	-4.2893389625	-1.3904236054	2.0553223632
H	-4.7062092436	-1.4013851187	3.0748616580
H	-5.0747019438	-1.0151921633	1.3866264125
H	-4.0727549425	-2.4338918949	1.7843552626
C	-2.0256952960	-1.0464546884	3.0602649632
H	-1.0648587434	-0.5157823741	2.9945806878
H	-2.4322475649	-0.8978346419	4.0727104864
H	-1.8366462839	-2.1234910884	2.9480540428
C	1.7454606656	-2.4664665864	1.4647575288
H	2.0196870717	-3.1698942434	2.2662993914
H	0.8432684906	-1.9158268846	1.7721948271
H	1.5083281144	-3.0636987696	0.5737579294
C	3.2492764859	-0.8678195949	2.5855206235
H	4.1465371616	-0.2377132371	2.5639930712

H	2.4047218552	-0.2565059409	2.9409272072
H	3.4236465238	-1.6575023683	3.3330225969
C	4.0933893510	-2.3703975174	0.7227916194
H	5.0313713126	-1.8198947074	0.6106217769
H	4.2765302446	-3.1842472874	1.4423699136
H	3.8513625775	-2.8456821614	-0.2398341243
C	3.5727280615	2.2408684174	1.0080988371
H	4.3753060956	2.9924220475	0.9464166442
H	2.6147216542	2.7629878235	0.9042315891
H	3.5986752143	1.8130359008	2.0188749644
C	5.2086746480	0.5988206171	0.1018879934
H	5.9307564602	1.4140232696	-0.0638633482
H	5.3964893202	0.2050192930	1.1077084158
H	5.4335670891	-0.1845984633	-0.6350940577
C	3.7859322408	1.8111022211	-1.4738310999
H	4.2588444501	1.1500954718	-2.2137747901
H	2.7894390628	2.0667165562	-1.8526397877
H	4.3762630760	2.7400054244	-1.4454071314

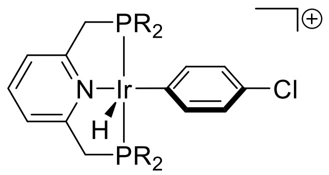

(tBuL1)Ir(H)(Ar)

R = tBu

82

(tBuL1)Ir(H)(Ar)

H	0.0985007061	0.2947131644	1.6168733188
Ir	0.1838266846	0.1269010174	0.0991830293
P	-0.8291088183	2.2099678575	-0.1420686238
P	1.7027139357	-1.6056354828	0.3267233574
N	1.9762531951	1.3154825121	-0.0110263940
C	0.5956228622	3.1246358031	-0.9053055960
C	1.9264175449	2.5834412585	-0.4741202365
C	3.0852652121	3.3499423333	-0.5888111427
C	4.3099105355	2.7919494514	-0.2433017530
C	4.3476317181	1.4852146626	0.2316077705
C	3.1592755944	0.7697896859	0.3558973447
C	3.1331354444	-0.6037672454	0.9526787134
C	-2.1908849177	2.3873604666	-1.4263544218
C	-1.1802894318	3.1276844840	1.4663380065
C	2.2462242572	-2.1996709364	-1.3859179353
C	1.5023135953	-2.9813061566	1.5918637292
H	0.4983659156	2.9707628387	-1.9931098825
H	0.5418408427	4.2111765215	-0.7434831629
H	3.0200348367	4.3728169194	-0.9628102063
H	5.2297033079	3.3728148239	-0.3391430695
H	5.2903825547	1.0211325362	0.5249911129
H	4.1047041604	-1.1037251615	0.8472106376
H	2.9472335232	-0.4922157263	2.0330970871

H	-4.0868267574	-2.1198307695	2.1129123038
C	-3.4583304134	-2.0128253346	1.2267718045
C	-2.3309440854	-1.1924445299	1.2488817779
H	-2.1059743557	-0.6729816440	2.1825040867
C	-1.4914845123	-1.0293221885	0.1314469283
C	-1.8571006146	-1.7559526945	-1.0190224715
H	-1.2456841338	-1.6944373728	-1.9258951634
C	-2.9814755034	-2.5786483603	-1.0692721613
H	-3.2349720507	-3.1256971647	-1.9792346618
C	-3.7825419580	-2.7043981171	0.0623234627
Cl	-5.1822061429	-3.7288731896	0.0217977959
C	3.7456054857	-2.4820705173	-1.4750487286
H	4.0731213622	-3.2509957100	-0.7619499653
H	3.9822097651	-2.8511667140	-2.4857452800
H	4.3496169247	-1.5772859591	-1.3144498133
C	1.4583584859	-3.4434773435	-1.7983934101
H	1.7628234709	-4.3291200430	-1.2259263085
H	0.3735236625	-3.3117537504	-1.6822351603
H	1.6606255719	-3.6556817093	-2.8601486469
C	1.8943703542	-1.0499730713	-2.3424170510
H	2.0847511220	-1.3631630290	-3.3813409306
H	0.8224391222	-0.7793108105	-2.2882472342
H	2.4923932478	-0.1476184996	-2.1554161642
C	1.2438105826	-2.3011620232	2.9415583789
H	0.3801393489	-1.6222234227	2.8915469727
H	1.0124402232	-3.0783431129	3.6858121930
H	2.1126132199	-1.7448107918	3.3212140389
C	0.2725192389	-3.8338341252	1.2591053352
H	0.1131869045	-4.5445671574	2.0850015002
H	-0.6349390863	-3.2235014002	1.1611457597
H	0.3946385798	-4.4228790269	0.3441038948
C	2.7558875795	-3.8522792230	1.6714957241
H	2.6298677948	-4.5942355370	2.4755813598
H	2.9303823145	-4.4102404693	0.7412076486
H	3.6608678292	-3.2712414570	1.9037604409
C	-1.7908452336	1.4552991648	-2.5762739573
H	-1.7503915612	0.4091684483	-2.2476875070
H	-2.5487073095	1.5223767694	-3.3722522955
H	-0.8253700088	1.7271223390	-3.0312176377
C	-2.3413584181	3.8074719622	-1.9746219259
H	-3.1007019437	3.7946557364	-2.7724266371
H	-2.6763466761	4.5274048757	-1.2201212956
H	-1.4117207038	4.1856600844	-2.4245218291
C	-3.5093415476	1.8939318831	-0.8242454011
H	-3.4126232178	0.8855628071	-0.3948748393
H	-3.8905010734	2.5729879173	-0.0489635082
H	-4.2703755950	1.8475581395	-1.6187799495
C	-2.0623677135	2.2479764231	2.3548102436
H	-1.5393164304	1.3253169779	2.6397331812
H	-2.3045484855	2.7973122787	3.2779633551
H	-3.0067215660	1.9638103019	1.8735764035
C	-1.8452624162	4.4843806296	1.2438279256

H	-2.8703908527	4.3870201748	0.8628117567
H	-1.9064078129	5.0120193598	2.2086613363
H	-1.2762409772	5.1286926466	0.5571685209
C	0.1661363661	3.3387906518	2.1682563341
H	0.7176558242	2.3963245008	2.2982416076
H	0.8132939689	4.0496735472	1.6348211246
H	-0.0196247803	3.7570860682	3.1696510223

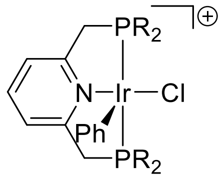

(tBuL1)Ir(Cl)(Ph)

R = tBu

82

(tBuL1)Ir(Cl)(Ph)

Ir	-0.0064331912	-0.0087245518	0.2959029776
P	2.3230729046	0.4255393985	0.1558248229
P	-2.3379334338	0.0231434369	0.1001562744
N	-0.0568080169	0.6157377180	-1.6712311872
C	2.2629906302	1.4050984174	-1.4116199375
C	1.0503275283	1.1487357727	-2.2500179182
C	1.0374597047	1.5550713377	-3.5829264411
C	-0.1353459727	1.4780859461	-4.3202624928
C	-1.2793733561	0.9931213021	-3.6987843521
C	-1.2176036026	0.5528239847	-2.3806310965
C	-2.4161048506	-0.0671586170	-1.7378299898
C	2.9436536823	1.7024333193	1.4097964411
C	-2.8705045460	1.7725047011	0.5716438220
C	-3.5309650009	-1.3006260033	0.7201650591
H	1.9516357151	1.9570218105	-4.0218802780
H	-0.1617259167	1.8036849435	-5.3621214971
H	-2.2268791233	0.9289349129	-4.2352372243
H	-3.3476030371	0.3214998450	-2.1706916200
H	-2.3835696536	-1.1472093028	-1.9556776326
H	0.0535324077	-5.0334552831	1.2030972024
C	0.0227836600	-4.2900612307	0.4025984110
C	-0.0688332534	-4.6956330930	-0.9271307310
H	-0.1125440917	-5.7559271299	-1.1838145624
C	-0.0926382815	-3.7243473946	-1.9231320172
H	-0.1465287833	-4.0163856459	-2.9750018064
C	-0.0393635234	-2.3673298460	-1.5963642657
H	-0.0302265186	-1.6506292793	-2.4168610786
C	0.0285095692	-1.9468614674	-0.2593091289
C	0.0713208352	-2.9366022287	0.7372079341
H	0.1407154673	-2.6494414852	1.7851860021
H	3.1817145844	1.3104454424	-2.0072289257
H	2.2129301113	2.4623199736	-1.1010229890
Cl	-0.0057225111	-0.2757937559	2.6287963463
C	-1.6810976017	2.6780063860	0.2278678279

H	-1.8943714485	3.7020489991	0.5730184988
H	-0.7579757359	2.3635340492	0.7455147388
H	-1.4808838307	2.7276907495	-0.8514573916
C	-4.0937537782	2.2760845552	-0.1920087352
H	-3.9239615742	2.2956495089	-1.2786434315
H	-4.9966722584	1.6885930983	0.0074355431
H	-4.3014404357	3.3125281912	0.1187401457
C	-3.0965057512	1.8300787716	2.0846443753
H	-3.9990070972	1.2873353452	2.3936135431
H	-2.2378885646	1.4212776801	2.6388827633
H	-3.2284063409	2.8811494294	2.3860649773
C	-4.9865601704	-0.8377382196	0.6342406173
H	-5.2758959950	-0.5494619373	-0.3875446477
H	-5.6333093689	-1.6790254758	0.9285190310
H	-5.2101590541	-0.0052717205	1.3132253401
C	-3.1614076512	-1.6438777252	2.1660756392
H	-3.8553342142	-2.4180707289	2.5288113868
H	-2.1386207927	-2.0330136734	2.2396742401
H	-3.2353374135	-0.7835208982	2.8422314850
C	-3.3798342149	-2.5596270642	-0.1416639446
H	-3.7916160162	-2.4260861420	-1.1526413661
H	-2.3424825650	-2.9064369805	-0.2194856229
H	-3.9613814742	-3.3643638919	0.3336742694
C	1.7684678031	2.6437493660	1.6967960569
H	1.3968449671	3.1474353491	0.7900851391
H	0.9398236176	2.1153876041	2.1880020258
H	2.1088983908	3.4359392963	2.3816264775
C	4.1125534025	2.5460838550	0.8905705823
H	3.8676295412	3.1003284314	-0.0273269013
H	4.3561727904	3.2979857863	1.6574830657
H	5.0208701795	1.9634052389	0.7079993065
C	3.3378799646	0.9951335452	2.7087722766
H	2.5260020145	0.3548363240	3.0815797238
H	4.2492354804	0.3932138154	2.5957699260
H	3.5457281537	1.7569627253	3.4765233967
C	5.0239465282	-0.4224185881	-0.1727717515
H	5.6830355427	-1.2529929024	-0.4705206392
H	5.1697726808	0.3852061416	-0.9057386669
H	5.3673923595	-0.0737367794	0.8091218171
C	3.5869631412	-0.9421542401	-0.1443974811
C	3.4317606639	-1.9989014106	0.9526986408
H	4.1698882741	-2.7978824265	0.7799593256
H	3.6082464234	-1.5942280574	1.9571190816
H	2.4349239781	-2.4551703014	0.9362750209
C	3.2762393749	-1.5698285243	-1.5061444232
H	3.4409271900	-0.8710872096	-2.3396578174
H	3.9588247003	-2.4200813457	-1.6593911525
H	2.2536591242	-1.9580792621	-1.5639049186

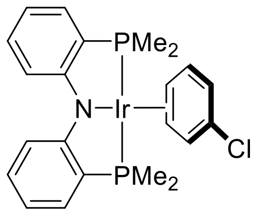

(L3)Ir-SC-1

52
(L3)Ir-SC-1

Ir	-0.17498	-0.54454	0.32688
P	-0.92237	1.62121	-0.01401
P	1.09623	-2.37079	0.83300
N	1.62525	0.41062	0.56945
C	-1.23968	2.25366	-1.70317
H	-1.29764	3.35214	-1.70278
H	-0.41778	1.93135	-2.35614
H	-2.18108	1.82979	-2.07734
C	-2.28533	2.35387	0.96080
H	-2.14417	2.09119	2.01756
H	-2.30053	3.44722	0.85091
H	-3.24132	1.94021	0.62002
C	0.99149	-3.90041	-0.16638
H	1.15654	-3.64928	-1.22273
H	1.73580	-4.64428	0.15230
H	-0.01155	-4.33860	-0.05511
C	1.03671	-3.00687	2.54875
H	1.82351	-3.75662	2.71881
H	0.05303	-3.45690	2.74719
H	1.17932	-2.16178	3.23494
Cl	-4.79498	0.46717	-2.19172
C	-3.69820	-0.34155	-1.11138
C	-3.99746	-0.35956	0.28702
H	-4.89833	0.12330	0.66912
C	-3.15189	-1.00395	1.14026
H	-3.38160	-1.04747	2.20591
C	-1.96272	-1.68855	0.69856
H	-1.69738	-2.55737	1.30918
C	-1.67561	-1.66415	-0.72025
H	-1.14683	-2.50611	-1.17803
C	-2.58817	-0.96999	-1.59721
H	-2.39873	-0.99546	-2.67347
C	0.52755	2.55911	0.55712
C	1.66149	1.77176	0.86222
C	2.80238	-0.31731	0.41084
C	2.76328	-1.72395	0.57973
C	2.73275	2.41764	1.51694
C	2.70528	3.78518	1.75893
C	1.61506	4.56743	1.37298
C	0.52228	3.93468	0.78859
H	3.58673	1.83375	1.85991
H	3.55238	4.24731	2.27283

H	1.60381	5.64330	1.55721
H	-0.36170	4.52298	0.52562
C	3.90926	-2.50814	0.45277
C	5.13010	-1.93307	0.11403
C	5.16955	-0.55809	-0.12803
C	4.03833	0.23643	0.01123
H	3.84222	-3.58974	0.60168
H	6.02648	-2.54679	0.00801
H	6.10441	-0.09015	-0.44796
H	4.10359	1.29947	-0.21935

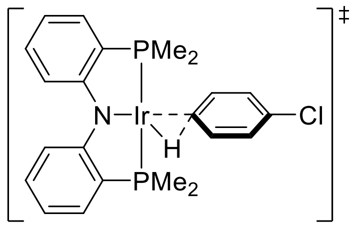

(L3)Ir-H-TS

52

(L3)Ir-H-TS

Ir	0.06844	-0.36483	-0.43318
P	-1.27898	-2.19064	-0.61119
P	0.91108	1.74865	-0.16771
N	-1.67840	0.54239	0.12046
C	-1.85917	-2.71921	-2.26854
H	-2.67035	-3.45780	-2.18189
H	-2.22585	-1.83530	-2.80661
H	-1.02484	-3.15797	-2.83516
C	-0.83082	-3.79435	0.15990
H	-0.61697	-3.63084	1.22505
H	-1.63973	-4.53373	0.06601
H	0.06919	-4.19495	-0.33038
C	1.84640	2.58533	-1.50399
H	1.31999	2.43661	-2.45608
H	1.93646	3.66257	-1.30238
H	2.85332	2.15322	-1.57302
C	1.91549	2.14924	1.31145
H	2.07319	3.23446	1.40101
H	2.88804	1.64121	1.24153
H	1.38733	1.78158	2.20085
C	-4.79584	-1.96462	1.52047
C	-3.77477	-2.45407	0.71384
C	-2.74089	-1.62205	0.28242
C	-2.71351	-0.24070	0.60687
C	-3.72614	0.21859	1.48262
C	-4.73787	-0.62530	1.91816
H	-5.60031	-2.61762	1.86377
H	-3.76863	-3.51063	0.42974
H	-3.69754	1.24464	1.84792
H	-5.49402	-0.22901	2.60134
C	-3.02191	2.61544	-0.07636

C	-1.79372	1.92085	0.03425
C	-0.61528	2.70693	-0.02458
C	-0.67262	4.10105	-0.06731
C	-1.89225	4.76682	-0.10240
C	-3.06167	4.00100	-0.13623
H	-3.95375	2.05484	-0.14422
H	0.25770	4.67672	-0.09157
H	-1.93408	5.85694	-0.13969
H	-4.03248	4.49572	-0.22716
C	4.38189	-0.66866	0.67059
C	3.34622	-1.39629	1.24141
H	3.41110	-1.71417	2.28266
C	2.23613	-1.72759	0.45121
C	2.16030	-1.26042	-0.86842
C	3.22335	-0.53895	-1.42533
H	3.14840	-0.18151	-2.45454
C	4.35175	-0.25578	-0.66899
H	5.19679	0.29327	-1.08652
H	1.28489	-1.52488	-1.52881
H	1.47070	-2.39058	0.85392
Cl	5.76519	-0.25952	1.63725

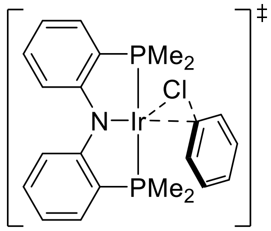

(L3)Ir-Cl-TS

52

(L3)Ir-Cl-TS

Ir	-0.41598	-0.33295	-0.57159
P	0.72760	-2.27898	-0.27047
P	-0.65328	1.81902	-1.30954
N	1.24876	0.49613	0.25511
C	0.41357	-3.62272	0.94604
H	1.27881	-4.29968	1.00510
H	0.23301	-3.17880	1.93388
H	-0.46748	-4.20738	0.64366
C	0.98222	-3.25256	-1.80383
H	1.33645	-2.57489	-2.59103
H	1.71468	-4.05972	-1.65268
H	0.02255	-3.68884	-2.11788
C	-2.17014	2.81906	-1.55754
H	-2.66867	2.98792	-0.59415
H	-1.93023	3.79242	-2.01028
H	-2.85366	2.27928	-2.22896
C	0.24294	2.12962	-2.87979
H	0.35267	3.20880	-3.06626
H	-0.29995	1.65987	-3.71262
H	1.23361	1.66342	-2.80064

C	1.17706	1.82462	0.66226
C	1.87015	2.35951	1.76600
C	1.72250	3.69687	2.11791
C	0.88500	4.54961	1.39647
C	0.16545	4.02895	0.32231
C	0.29573	2.68866	-0.03343
H	2.50093	1.70827	2.37153
H	2.26202	4.07596	2.98993
H	0.77518	5.59731	1.68319
H	-0.51776	4.67491	-0.23601
C	3.50358	-2.43414	0.28010
C	4.74847	-1.86722	0.52402
C	4.82905	-0.47500	0.63694
C	3.70101	0.32691	0.55168
C	2.41543	-0.23520	0.35528
C	2.35818	-1.64191	0.17808
H	3.42114	-3.51749	0.14751
H	5.64273	-2.48878	0.59735
H	5.80219	0.00210	0.78202
H	3.81469	1.40896	0.61090
H	-2.73433	-2.48314	3.40961
C	-2.85738	-1.67846	2.68064
C	-2.54607	-1.92248	1.34791
H	-2.20149	-2.90088	1.01884
C	-2.66332	-0.87482	0.42466
Cl	-2.52696	-1.26436	-1.32660
C	-3.23601	0.34962	0.79491
H	-3.42442	1.10997	0.04021
C	-3.53697	0.56978	2.13483
H	-3.94632	1.53760	2.43387
C	-3.33364	-0.43028	3.08675
H	-3.57417	-0.24806	4.13579

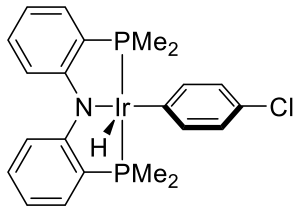

(L3)Ir(H)(Ar)

52

(L3)Ir(H)(Ar)

Ir	0.1145333101	0.0662317379	-0.2674152466
P	-0.0687904062	-2.1794118568	-0.6430000610
P	-0.2711618216	2.2998596805	0.0270356165
N	-1.9313891511	-0.0903565178	0.1700282087
C	-0.0654568271	-2.7901706322	-2.3631928378
H	-0.3501546887	-3.8518553344	-2.4013489770
H	-0.7804316043	-2.1982247450	-2.9493848445
H	0.9403196914	-2.6640581756	-2.7889365469
C	1.0776565123	-3.3091103716	0.2169718424

H	1.0311041602	-3.1097771218	1.2958424890
H	0.8377103456	-4.3653014258	0.0274366879
H	2.0972247043	-3.0900780583	-0.1324834968
C	0.2380303693	3.4449711707	-1.2967728344
H	-0.1753162758	3.0889381500	-2.2493925530
H	-0.1082136344	4.4707497681	-1.1051785300
H	1.3357243329	3.4324365606	-1.3573532252
C	0.3081083950	3.1292047287	1.5500380194
H	-0.0823795035	4.1557266319	1.6132023969
H	1.4073889298	3.1500892009	1.5519300853
H	-0.0392429530	2.5585241805	2.4218520589
C	4.9379767204	0.0957153837	0.4078100947
C	4.0422415767	0.2718380966	1.4592257328
H	4.4150675649	0.3945032061	2.4781995221
C	2.6751877542	0.2897317174	1.1878512450
C	2.1475137573	0.1295098307	-0.1107570901
C	3.0985087677	-0.0434086980	-1.1364326087
H	2.7607504962	-0.1730814070	-2.1687795204
C	4.4723725790	-0.0624842779	-0.8936837699
H	5.1834375944	-0.1992697552	-1.7111665428
H	0.5197715804	0.2913335070	-1.7325850628
H	1.9898236544	0.4293525474	2.0329467830
Cl	6.6534724730	0.0769919274	0.7256715146
C	-3.4001971088	-3.9941743452	0.9577893869
C	-2.2056477592	-3.7903067418	0.2746741704
C	-1.7188449995	-2.5033093258	0.0429568967
C	-2.4480385701	-1.3513284378	0.4536599468
C	-3.6192962922	-1.5877003079	1.2079516358
C	-4.0807411989	-2.8766716494	1.4445738684
H	-3.7743484549	-5.0030098076	1.1408395915
H	-1.6251892601	-4.6525276308	-0.0673860253
H	-4.1584185426	-0.7457644855	1.6407524758
H	-4.9903522028	-3.0096073642	2.0363311745
C	-4.1231868491	1.0552638340	-0.0858312473
C	-2.7202926495	1.0525082455	0.0859286321
C	-2.0849215827	2.3271868144	0.0754501379
C	-2.8240554938	3.5087581402	0.0101402620
C	-4.2109779404	3.4838529526	-0.0932645038
C	-4.8422163867	2.2413434516	-0.1691179953
H	-4.6553561616	0.1108456346	-0.1893212095
H	-2.2999675804	4.4693614445	0.0189551837
H	-4.7849790414	4.4105831420	-0.1482152146
H	-5.9253992376	2.1898655495	-0.3087431584

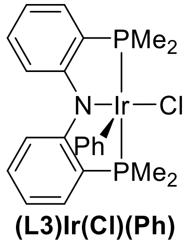

(L3)Ir(Cl)(Ph)

52

(L3)Ir(Cl)(Ph)

Ir	0.18288	-0.62487	-0.70465
P	-2.10884	-0.83115	-0.75762
P	2.37687	0.03936	-0.50482
N	-0.24181	1.31081	-0.16201
C	-2.57299	0.69811	0.08588
C	-1.50392	1.60137	0.33809
C	0.77288	2.25131	-0.25217
C	2.12859	1.82160	-0.31893
C	-2.91899	-0.84244	-2.39273
H	-4.01331	-0.79609	-2.29243
H	-2.57188	0.02421	-2.97128
H	-2.62875	-1.76275	-2.91980
C	-2.88993	-2.20765	0.13867
H	-2.52649	-2.20521	1.17455
H	-3.98592	-2.12531	0.12310
H	-2.58265	-3.14680	-0.34302
C	3.43796	-0.18998	-1.96958
H	2.95070	0.26932	-2.83980
H	4.43120	0.25900	-1.82574
H	3.53482	-1.26955	-2.15305
C	3.40016	-0.55896	0.87733
H	4.35533	-0.01555	0.91277
H	3.58741	-1.63289	0.73608
H	2.85207	-0.41834	1.81768
H	0.35269	-4.14095	2.97091
C	0.29740	-3.06345	2.79264
C	0.26568	-2.17633	3.86774
H	0.29169	-2.54721	4.89501
C	0.20344	-0.80948	3.60964
H	0.17954	-0.09458	4.43667
C	0.17063	-0.33019	2.29846
H	0.12970	0.74558	2.13458
C	0.19607	-1.21598	1.20998
C	0.26434	-2.59434	1.47961
H	0.29865	-3.30276	0.65106
Cl	0.63574	-2.62117	-1.89881
C	1.60471	4.54264	-0.43330
H	1.38188	5.60898	-0.52500
C	2.93390	4.11009	-0.40488
H	3.75763	4.82472	-0.44872
C	3.17816	2.74322	-0.37075
H	4.20874	2.37721	-0.40779
C	-3.08270	2.92461	1.65291
C	-4.13944	2.07105	1.32823
H	-5.14836	2.26070	1.69863
C	-3.86246	0.94715	0.55897
H	-4.66131	0.23355	0.33665
H	-3.25835	3.78246	2.30737
C	-1.79722	2.69954	1.17946
H	-0.99652	3.36767	1.49430

C	0.54994	3.64455	-0.36754
H	-0.47020	4.02014	-0.43666

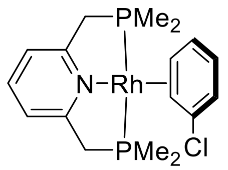

Rh-SC-1

46

Rh-SC-1

Rh	-0.0018574783	0.2997037345	0.2837914084
P	-0.7014496717	-1.9066835060	0.2229041213
P	1.2825079981	2.1758115899	0.1615992433
N	1.8571694760	-0.6154709942	-0.0782568808
C	0.8856740553	-2.6692834179	0.7862468653
C	2.0420387205	-1.9283150604	0.2004224832
C	3.2628534554	-2.5554957542	-0.0389174847
C	4.3218620658	-1.8187796661	-0.5537170998
C	4.1279203078	-0.4708028376	-0.8320730200
C	2.8824894056	0.1053594009	-0.5987346304
C	2.6119071021	1.5370271816	-0.9406276716
C	-1.9123632275	-2.8574957018	1.2029336137
H	-1.6741963472	-3.9294986189	1.1310958563
H	-1.8835267624	-2.5406299540	2.2534241306
H	-2.9239293445	-2.6966967836	0.8128166277
C	-0.9226777823	-2.5615119396	-1.4665104528
H	-0.0950540077	-2.2141897276	-2.1004908560
H	-0.9587851133	-3.6610096277	-1.4722448108
H	-1.8570463534	-2.1604342071	-1.8849316340
C	2.1493251881	2.6965544415	1.6808559242
H	2.6181228273	1.8197193041	2.1486915275
H	2.9170085053	3.4543299162	1.4639808344
H	1.4221505812	3.1149528908	2.3912920396
C	0.7594795762	3.7437620607	-0.6082526235
H	1.6015652514	4.4472449285	-0.6854142865
H	-0.0290852830	4.2081155618	0.0018211087
H	0.3567009895	3.5515908018	-1.6125287460
H	0.8991841919	-2.5580896760	1.8845888187
H	0.9435909641	-3.7456774409	0.5645586595
H	3.3750369225	-3.6154715842	0.1930443683
H	5.2892571023	-2.2903908421	-0.7388989408
H	4.9321462350	0.1394078741	-1.2454077092
H	3.5308130497	2.1418939978	-0.9191555970
H	2.2024965819	1.5902720507	-1.9645355927
H	-4.2253365521	-0.6847397446	2.1493949008
C	-3.5465661949	-0.1267364229	1.5006058616
C	-3.7280402921	-0.1934115697	0.0990822381
H	-4.5344298524	-0.7902330570	-0.3310232052
C	-2.8878911888	0.5169583421	-0.7257386206
Cl	-3.1006483228	0.4683434292	-2.4418620218

C	-1.8579438685	1.3573478455	-0.2045006555
H	-1.4979538694	2.1527085907	-0.8591280552
C	-1.6924103280	1.4123853557	1.2060274972
H	-1.1717396581	2.2552571308	1.6667315964
C	-2.5415852017	0.6368024365	2.0467120778
H	-2.4322912612	0.7132629401	3.1304301442

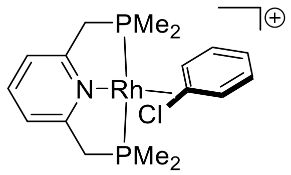

Rh-SC-2

46

Rh-SC-2

Rh	0.3021720417	0.3888920612	-0.0969682613
P	1.7075105555	2.1763424881	0.0388439796
P	-0.6132547579	-1.7095710537	-0.1633414954
N	2.0357737730	-0.6605315022	0.1030834514
C	3.0172554278	1.3608262642	1.0507859680
C	3.1461475984	-0.0643399790	0.6126860148
C	4.3469275173	-0.7572138636	0.7378224980
C	4.4133087743	-2.0938701151	0.3637655530
C	3.2717898721	-2.7010077766	-0.1446035127
C	2.0993729625	-1.9626150427	-0.2808251384
C	0.8633896014	-2.5603802638	-0.8750757307
C	2.5752066044	2.7391083183	-1.4647393050
H	3.3940726442	3.4321059392	-1.2197688281
H	2.9773894550	1.8658894673	-1.9969881679
H	1.8595920446	3.2436791501	-2.1289646281
C	1.3159922184	3.7151106580	0.9328058081
H	0.8622197122	3.4695462831	1.9025878584
H	2.2151202418	4.3295440504	1.0894054139
H	0.5877958633	4.2954292734	0.3480653584
C	-1.9956901063	-2.2468764106	-1.2227854355
H	-1.8919458301	-1.8024261472	-2.2218776526
H	-2.0199409540	-3.3437013806	-1.3066209396
H	-2.9421970409	-1.9022291497	-0.7835777996
C	-0.9011948603	-2.6023232074	1.4046106189
H	-1.0436459455	-3.6788682629	1.2263293544
H	-1.7978883920	-2.1986207146	1.8936785344
H	-0.0415066649	-2.4504593560	2.0719287075
H	3.9864805252	1.8813983014	1.0270771691
H	2.6489779417	1.3879659688	2.0909308736
H	5.2191918214	-0.2429258087	1.1436484534
H	5.3441865519	-2.6552453279	0.4661529929
H	3.2818191509	-3.7465051047	-0.4559046380
H	0.8410573208	-2.3441319095	-1.9572322062
H	0.8444090827	-3.6539970362	-0.7536832927
H	-6.0413855980	-0.1160620330	-1.3081576860
C	-5.2409029420	-0.0039976512	-0.5737753934

C	-5.3630009516	-0.5794963338	0.6903909686
H	-6.2624290368	-1.1422817435	0.9480575587
C	-4.3430608050	-0.4277215239	1.6312003258
C	-3.1923071005	0.2912064754	1.3121557278
H	-2.3814386101	0.4249824451	2.0303665308
C	-3.0932560273	0.8418713467	0.0394381411
Cl	-1.6338625884	1.7262158188	-0.3877140243
C	-4.0960711863	0.7178620292	-0.9136079892
H	-3.9822809500	1.1677843394	-1.9011802414
H	-4.4460509363	-0.8644028452	2.6272048773

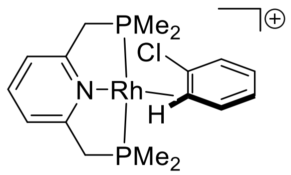

Rh-SC-3

46

Rh-SC-3

Rh	0.0928965078	0.2729141443	0.1163773358
P	-0.3690909839	-1.9667018182	-0.0990410561
P	1.1344685244	2.2893699094	0.3251795188
N	2.0184450970	-0.3916884664	-0.1382583159
C	1.0638008140	-2.3641462854	-1.1977364312
C	2.2551591443	-1.5871790944	-0.7363667516
C	3.5527449273	-2.0574849552	-0.9224370575
C	4.6303688645	-1.2810052782	-0.5152836421
C	4.3807656366	-0.0516997941	0.0832339489
C	3.0671145274	0.3672444801	0.2730502806
C	2.7484158867	1.6583731216	0.9587100952
C	-0.1174971676	-3.0579762736	1.3403103816
H	-0.1019106289	-4.1151360395	1.0357904989
H	0.8334558663	-2.8008382972	1.8272310892
H	-0.9306088652	-2.8949612890	2.0597102737
C	-1.7970041037	-2.6835954380	-0.9738742052
H	-2.0082390723	-2.0962152154	-1.8778848832
H	-1.5981927136	-3.7302750599	-1.2478568844
H	-2.6778928247	-2.6487921233	-0.3191251355
C	0.6377532450	3.5763562988	1.5155607892
H	0.4571115299	3.1184058486	2.4977329973
H	1.4089333075	4.3554229947	1.6080400314
H	-0.2976418932	4.0420196945	1.1729440271
C	1.5791271262	3.2252209613	-1.1794233116
H	2.3004736160	4.0242074810	-0.9507589383
H	0.6761700818	3.6759542598	-1.6151055250
H	2.0128408263	2.5383716171	-1.9194223625
H	1.2825634438	-3.4407908798	-1.2618382059
H	0.7748801683	-2.0225595254	-2.2067434049
H	3.7058011086	-3.0262200736	-1.4000313872
H	5.6545324702	-1.6298256131	-0.6626544319
H	5.1991314216	0.5855870533	0.4211470213

H	2.5938719789	1.4674989706	2.0348679071
H	3.5714536197	2.3827019550	0.8648964465
H	-1.3142332857	1.1341139932	0.8421752946
C	-2.0935574440	0.8473437344	0.0456585124
C	-2.2595157392	1.5868073142	-1.1332717634
H	-1.4691678702	2.2687972872	-1.4515106815
C	-3.4145289793	1.4472836084	-1.8986263728
C	-4.4411179480	0.6144994764	-1.4548290131
H	-5.3584300524	0.5170227723	-2.0397040673
C	-4.3239032604	-0.0792471848	-0.2501366272
H	-5.1365445470	-0.7072036029	0.1184096481
C	-3.1532473674	0.0432478424	0.4895513250
Cl	-2.9995926183	-0.8173650477	1.9853862047
H	-3.5245799324	2.0062001917	-2.8297420498

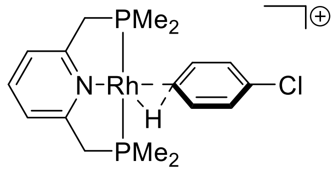

Rh-H-TS

46

Rh-H-TS

Rh	0.3259462168	-0.0677487114	-0.3187799268
P	0.2321966852	2.2167649535	-0.3532626834
P	1.0392826864	-2.2482932213	-0.3127781146
N	2.3480139782	0.2831544809	0.2621517679
C	2.0384028885	2.5921613534	-0.4853505931
C	2.8733476591	1.5274825490	0.1626752339
C	4.1691334355	1.7931573980	0.5995008128
C	4.9423830801	0.7565731913	1.1099206704
C	4.4017065999	-0.5222385426	1.1823965879
C	3.0901470602	-0.7327126158	0.7633662075
C	2.4429809658	-2.0825513418	0.8824580534
C	-0.5284457246	3.2481784150	-1.6445118349
H	-0.1923058790	4.2916249927	-1.5526033246
H	-0.2637652807	2.8609254548	-2.6376679135
H	-1.6204150055	3.2191924206	-1.5302042289
C	-0.3063920885	2.9820463354	1.2106666976
H	0.2414333668	2.5314085115	2.0501385636
H	-0.1440648503	4.0700535669	1.2075676418
H	-1.3767274374	2.7702033737	1.3476207740
C	1.8220316058	-2.7902209271	-1.8674242661
H	2.5048690712	-2.0070802476	-2.2245281219
H	2.3769296242	-3.7298295195	-1.7275926144
H	1.0422345151	-2.9380490183	-2.6277619712
C	0.1212912101	-3.7132021736	0.2579540283
H	0.7522387734	-4.6126811006	0.2079131697
H	-0.7599984005	-3.8531284244	-0.3840709040
H	-0.2239096451	-3.5701891699	1.2906034906
H	2.2633424002	2.5974056634	-1.5667839065

H	2.2941333713	3.5917378053	-0.1018315925
H	4.5694292933	2.8048605233	0.5199462516
H	5.9644191359	0.9440395175	1.4464707995
H	4.9842713305	-1.3566538556	1.5754680928
H	3.1856347477	-2.8893393402	0.7844921067
H	1.9984963953	-2.1788856874	1.8888306431
C	-4.4386236767	-0.3740619543	0.3308266906
C	-3.5220224381	-1.1110720603	1.0791059528
H	-3.8689748942	-1.7187622975	1.9167364897
C	-2.1712211987	-1.0716728469	0.7419973926
C	-1.6974762610	-0.2666086828	-0.3083729740
C	-2.6513813104	0.4423014955	-1.0582575692
H	-2.3406817594	1.0404541028	-1.9177103493
C	-4.0089384763	0.3956420882	-0.7470107449
H	-4.7356632179	0.9520603682	-1.3414918414
H	-0.5547604362	-0.4227041084	-1.5189547787
Cl	-6.1203697261	-0.4305805439	0.7344879545
H	-1.4760630593	-1.6631402072	1.3413152622

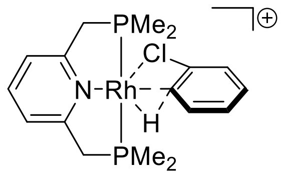

Rh-H-TS'

46

Rh-H-TS’

Rh	0.0225154872	-0.0209935020	-0.4248325744
P	0.1467956036	-2.2819886254	-0.1771704375
P	-0.7291756352	2.1258533162	-0.7402996289
N	-1.9864476304	-0.3227253235	0.1332599161
C	-1.6481692067	-2.6944570686	-0.3530162021
C	-2.5044699578	-1.5743532710	0.1596716864
C	-3.8021542302	-1.7990859285	0.6123762101
C	-4.5816273118	-0.7191377114	1.0122442909
C	-4.0448037813	0.5621760560	0.9642577342
C	-2.7317701835	0.7361623353	0.5325281781
C	-2.0834474593	2.0897605120	0.5178169044
C	0.9983794267	-3.3979551080	-1.3322368852
H	0.7304196222	-4.4463952065	-1.1345193262
H	0.7352142222	-3.1357583172	-2.3658276860
H	2.0827112070	-3.2768680054	-1.2025538485
C	0.6236191343	-2.8876052849	1.4738617502
H	0.0287442813	-2.3693226535	2.2388580658
H	0.4817717552	-3.9745393458	1.5666829196
H	1.6819825443	-2.6396400901	1.6400865729
C	-1.5938297877	2.4146902637	-2.3195440493
H	-2.2707230847	1.5736443157	-2.5237386600
H	-2.1668215233	3.3536301437	-2.2933803105
H	-0.8542732751	2.4639227860	-3.1310088158
C	0.1822493099	3.6685242773	-0.4352075256

H	-0.4843043311	4.5406709055	-0.5045830138
H	0.9752531176	3.7620715883	-1.1907625313
H	0.6512164516	3.6284011926	0.5561517264
H	-1.8257283111	-2.8034721540	-1.4375141506
H	-1.9181600864	-3.6530761692	0.1154799428
H	-4.1983000839	-2.8152468729	0.6340713814
H	-5.6051165281	-0.8762664759	1.3597186980
H	-4.6305339761	1.4287452770	1.2739579545
H	-2.8272350611	2.8889362543	0.3783680374
H	-1.5905450946	2.2648641511	1.4902227939
H	5.8883491360	0.1244670121	0.6179456525
C	4.8314980699	0.1096618594	0.3445252066
C	3.8905149515	0.6914904699	1.1909270986
H	4.1899568805	1.1466504469	2.1368830776
C	2.5450682885	0.6782848523	0.8347970586
Cl	1.4082401116	1.3481815324	1.9893460802
C	2.0788582977	0.0856842227	-0.3534549691
C	3.0595183458	-0.4970234922	-1.1777343073
H	2.7512670751	-0.9539609606	-2.1215250719
C	4.4119344780	-0.4896579526	-0.8397181185
H	5.1394734364	-0.9477810904	-1.5131247184
H	1.0990700229	0.3867904948	-1.4645282205

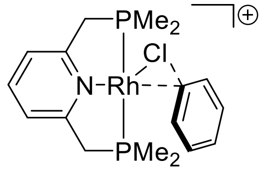

Rh-Cl-TS

46

Rh-Cl-TS

Rh	-0.1199999619	-0.0493190752	-0.5791059514
P	0.6457235243	-2.2253516184	-0.6223980851
P	-0.0551598592	2.2523504353	-0.5677034710
N	1.6968324335	0.1848131840	0.3854239658
C	1.5103030740	-2.1603159644	1.0091035341
C	2.2351855317	-0.8525149228	1.0759946508
C	3.4279230112	-0.7038346498	1.7788962617
C	4.0812244484	0.5227985640	1.7683048014
C	3.5467357823	1.5646350526	1.0177161870
C	2.3613308268	1.3665557361	0.3165318350
C	1.7840276180	2.4121389946	-0.5846174903
C	-0.2165960434	-3.8315975267	-0.6652617139
H	0.5144317685	-4.6475064939	-0.7601367307
H	-0.8045361281	-3.9924187147	0.2472684023
H	-0.8940407990	-3.8544856787	-1.5308432266
C	1.9820232599	-2.4485567434	-1.8464918539
H	2.6546468809	-1.5803944296	-1.8230158111
H	2.5568954635	-3.3662655609	-1.6516097131
H	1.5346692544	-2.5071665719	-2.8490216089
C	-0.4943427457	3.2242005695	0.9149092106

H	-0.0948295316	2.7211016002	1.8067877183
H	-0.0740166530	4.2385463057	0.8444657804
H	-1.5833485953	3.2920788534	1.0234094925
C	-0.6459819309	3.2473586624	-1.9707139219
H	-0.3108843826	4.2920859013	-1.8934382562
H	-1.7449186137	3.2232706035	-1.9880093518
H	-0.2827900744	2.8050921961	-2.9082305994
H	0.7092088263	-2.1892389896	1.7690576622
H	2.1900066785	-3.0052417221	1.1949998048
H	3.8330297848	-1.5525347358	2.3316288687
H	5.0106509223	0.6605982934	2.3247843376
H	4.0521112465	2.5292879023	0.9549111348
H	2.1357018650	3.4201474134	-0.3199160775
H	2.1076964688	2.2059929412	-1.6197703991
H	-3.0493700794	-2.3049423908	2.7499274613
C	-2.9644698089	-1.3906375749	2.1576578956
C	-2.2977120055	-1.4363876359	0.9333301475
H	-1.9230113348	-2.3803991352	0.5477889252
C	-2.1509497357	-0.2583474213	0.1922124675
Cl	-2.0638424762	-0.4303398683	-1.8244766612
C	-2.8310992052	0.9045790375	0.5803064060
H	-2.8562106368	1.7672122698	-0.0860918802
C	-3.4987625792	0.9299917399	1.7992044565
H	-4.0086026732	1.8455559110	2.1090142503
C	-3.5482755759	-0.2083215584	2.6069191042
H	-4.0826883696	-0.1849884177	3.5582525450

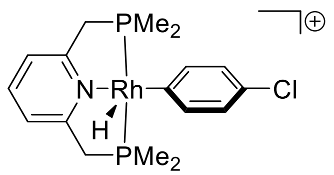

Rh(H)(Ar)

46

Rh(H)(Ar)

Rh	0.3102746550	-0.0058545867	-0.0663548851
P	0.5160167137	2.2596578553	-0.0447230941
P	0.6670476203	-2.2667252674	-0.2417440261
N	2.4504992026	0.0400441747	0.1417452879
C	2.3187914195	2.4179230899	-0.4379126087
C	3.1110799558	1.2062289568	-0.0230634926
C	4.4964286075	1.2647326034	0.1189432625
C	5.2012241362	0.0973162886	0.3921257267
C	4.5095266016	-1.1023262159	0.5241815013
C	3.1210687656	-1.1025197010	0.4060550620
C	2.3154688029	-2.3591789622	0.6067502455
C	-0.3448843692	3.3352179656	-1.2253227661
H	-0.0143156799	4.3798820965	-1.1337228481
H	-0.1665323714	2.9769698801	-2.2482707229
H	-1.4233228944	3.2728201650	-1.0210033434
C	0.2953881401	3.0623669116	1.5766328881

H	0.8936829605	2.5394853646	2.3362284427
H	0.5999521319	4.1189305142	1.5396985964
H	-0.7644335612	2.9994850691	1.8623459868
C	0.9493863593	-2.9281817570	-1.9123895403
H	1.6845997913	-2.2997939890	-2.4336212316
H	1.3134100384	-3.9652918841	-1.8721195375
H	0.0038493398	-2.8946080398	-2.4717926907
C	-0.3718620678	-3.5045394273	0.5904063653
H	0.0547475331	-4.5144374418	0.5033037887
H	-1.3684226703	-3.4857426027	0.1265451083
H	-0.4862924925	-3.2437083039	1.6513684858
H	2.3787432360	2.5040526911	-1.5376009523
H	2.7561003989	3.3412352387	-0.0281263701
H	5.0158437918	2.2163519017	-0.0045392769
H	6.2882497149	0.1211477067	0.4953300502
H	5.0377309466	-2.0342291990	0.7316452028
H	2.8900922419	-3.2504907279	0.3112095674
H	2.0957228057	-2.4724542861	1.6835782003
C	-4.4853439944	-0.0440186832	0.1461036640
C	-3.6881371155	-0.4375848179	1.2188367434
H	-4.1519172139	-0.7411036968	2.1591171091
C	-2.3017912358	-0.4463833824	1.0647317966
C	-1.6855564048	-0.0480808753	-0.1327910727
C	-2.5184693777	0.3407129693	-1.1919823465
H	-2.0884850707	0.6523350393	-2.1474861435
C	-3.9073300293	0.3400327630	-1.0621586739
H	-4.5458279543	0.6406878722	-1.8950260962
H	0.3297898636	0.0740689560	-1.5672574718
Cl	-6.2110903335	-0.0364915267	0.3162649586
H	-1.6961772842	-0.7763843651	1.9195709393

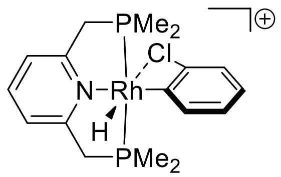

Rh(H)(Ar)'

46

Rh(H)(Ar)’

H	-0.0881399767	0.1262491999	1.7765178579
Rh	0.0719579578	0.0291879584	0.2695637209
P	-0.1669253995	-2.2401533595	0.4444272526
P	-0.2585247686	2.2823321859	0.2370523410
N	-2.0375166802	-0.0427310026	-0.0480339213
C	-1.7140143152	-2.4082140734	-0.5637340267
C	-2.6138383572	-1.2141960666	-0.3924378564
C	-3.9868143833	-1.2914162125	-0.6187406267
C	-4.7571611365	-0.1380983496	-0.5136663223
C	-4.1462810528	1.0615401311	-0.1637642614
C	-2.7749104251	1.0799292713	0.0843029814
C	-2.0799387356	2.3231786237	0.5697810597

C	1.0241975180	-3.3971359676	-0.2900804254
H	0.6354357040	-4.4256286899	-0.3052868034
H	1.2651599034	-3.0704398015	-1.3108906236
H	1.9469546599	-3.3641740975	0.3064309544
C	-0.5792018044	-2.9368014732	2.0728692964
H	-1.3981926265	-2.3552571333	2.5182252518
H	-0.8773907178	-3.9921628969	1.9895850943
H	0.2991851872	-2.8567958841	2.7284502432
C	-0.0219090453	3.1523829609	-1.3440527928
H	-0.5697085070	2.6282029658	-2.1390496102
H	-0.3668798036	4.1950668272	-1.2818598355
H	1.0478043528	3.1354610387	-1.5968977873
C	0.4920125982	3.3636135884	1.4871397412
H	0.1043935655	4.3906059648	1.4232041998
H	1.5788918567	3.3728934287	1.3221607664
H	0.2999357599	2.9531589168	2.4875590933
H	-1.3752005217	-2.4480240600	-1.6145962671
H	-2.2531982898	-3.3473886849	-0.3669672899
H	-4.4398937096	-2.2462481766	-0.8895395965
H	-5.8326040062	-0.1746617490	-0.7004704090
H	-4.7285736189	1.9788290124	-0.0639865290
H	-2.5608698160	3.2313102110	0.1758058111
H	-2.1788099668	2.3637539383	1.6692109302
H	5.1342473661	0.2937068012	1.8339124792
C	4.3930833697	0.1978703514	1.0368120100
C	3.0315055617	0.2031532221	1.3582020985
H	2.7231066426	0.3022821278	2.4023374122
C	2.0675987962	0.0794002405	0.3519473084
C	2.5538431496	-0.0421421865	-0.9468518541
Cl	1.2395662719	-0.1886945932	-2.1486539142
C	3.8879122195	-0.0536921846	-1.3173403975
H	4.1920589835	-0.1534010887	-2.3609536211
C	4.8215553418	0.0705042194	-0.2855727259
H	5.8876455944	0.0673484135	-0.5213385600

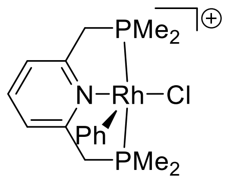

Rh(Cl)(Ph)

46

Rh(Cl)(Ph)

Rh	0.2089899715	-0.1921974227	-0.7231371087
P	-0.9653975184	-2.1541580724	-0.4424522762
P	1.0538769958	1.9369028196	-0.8532066831
N	-1.4499517855	0.7213872862	0.0636630798
C	-2.6515412468	-1.4020990585	-0.2970608611
C	-2.6067776171	0.0229704776	0.1834069275
C	-3.7497510803	0.6295807502	0.6998043807
C	-3.7124653457	1.9694113159	1.0655462134

C	-2.5239385749	2.6755155468	0.9214289219
C	-1.3923520156	2.0268857102	0.4347217847
C	-0.0666511317	2.7298376010	0.3769846886
C	-1.0612367053	-3.3283749883	-1.8215416816
H	-1.8146485404	-4.1074413635	-1.6353157087
H	-1.2944623235	-2.7914161350	-2.7508346699
H	-0.0705560601	-3.7893606181	-1.9409690398
C	-0.7036343485	-3.1646011333	1.0422913889
H	-0.7645276792	-2.5358916144	1.9399287024
H	-1.4442421292	-3.9757006792	1.0934800732
H	0.3087589659	-3.5907666498	0.9959505184
C	0.7813522167	2.7859822093	-2.4369966840
H	-0.2734436395	2.6926652789	-2.7318238409
H	1.0504144118	3.8504594398	-2.3706756457
H	1.4008073720	2.2957926522	-3.2018577217
C	2.7472725331	2.3280352390	-0.3506409348
H	2.9174936843	3.4142217832	-0.3380733579
H	3.4316648732	1.8562648832	-1.0701486440
H	2.9380873623	1.9016995260	0.6434763072
H	-4.6669081846	0.0467537842	0.7971821216
H	-4.6039678939	2.4603002123	1.4614887157
H	-2.4581088060	3.7269339285	1.2046177522
H	-0.1932373254	3.8114010287	0.2231552835
H	0.4330822694	2.5937995813	1.3530945819
H	4.0147209806	-1.2651817113	2.4363266298
C	2.9735051458	-0.9346258868	2.4045108821
C	2.2865658348	-0.6655791101	3.5868438132
H	2.7823612894	-0.7792990603	4.5529792533
C	0.9585561273	-0.2557721161	3.5204594471
H	0.4003892113	-0.0472081850	4.4367325702
C	0.3187568193	-0.1102479382	2.2855766679
H	-0.7280380126	0.1945464582	2.2832110290
C	1.0130021123	-0.3702933265	1.0981419742
C	2.3482607410	-0.7904127347	1.1645220789
H	2.9005620102	-1.0093387755	0.2507970615
H	-3.3342215422	-2.0017379415	0.3239846981
H	-3.0745602437	-1.4101233826	-1.3175662284
Cl	1.8018532835	-1.1100735516	-2.1301862442

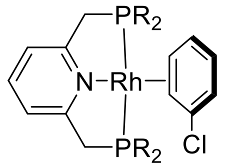

(tBuL1)Rh-SC-1

R = tBu

82

(tBuL1)Rh-SC-1

Rh	-0.1114988113	-0.1126675083	0.1431065767
P	2.1484575552	0.6360635923	0.2290684847
P	-2.4523304048	-0.0662181932	0.0555721979

N	-0.4124733952	1.8494048842	-0.5808097721
C	1.7356190274	2.4375688605	0.3619318577
C	0.5254758912	2.8058384261	-0.4104288187
C	0.3506482639	4.1035909175	-0.8904705323
C	-0.8259278524	4.4405020104	-1.5435381789
C	-1.8006334906	3.4618035260	-1.6960218023
C	-1.5669037628	2.1787142868	-1.2100136201
C	-2.5787900060	1.1020429323	-1.3675285490
C	3.2836237505	0.5160942574	1.7538719366
C	3.1345420721	0.5533661328	-1.3873534068
C	-3.1488416252	0.8718234515	1.5570923990
C	-3.6379153600	-1.4477552353	-0.5006772121
H	1.5158300288	2.6056750212	1.4294223093
H	2.5823849835	3.0881657132	0.1069958893
H	1.1392539262	4.8403919136	-0.7321747703
H	-0.9853757434	5.4518162063	-1.9232574081
H	-2.7444879070	3.6785348135	-2.1979846770
H	-3.5845759283	1.5113506333	-1.5325982474
H	-2.3116356349	0.5023702316	-2.2512008853
H	2.7479894055	-3.7081032459	2.1512770596
C	1.9621323540	-3.2857575831	1.5212317033
C	2.0186239517	-3.4944595402	0.1244164948
H	2.8377302903	-4.0605566410	-0.3218722682
C	0.9972408884	-3.0205686174	-0.6623907879
Cl	0.9578293837	-3.3634850640	-2.3599788830
C	-0.1115371530	-2.3231885135	-0.1130359166
H	-1.0141892040	-2.3344095254	-0.7167776250
C	-0.1465734256	-2.1055740025	1.2873316290
H	-1.0894978540	-1.8850887532	1.7906178506
C	0.9133237100	-2.6017532712	2.0916653385
H	0.8461442016	-2.5123227887	3.1776103738
C	-4.3038290755	1.8061624799	1.1943712306
H	-4.6431304858	2.3224633106	2.1067017916
H	-3.9896803054	2.5855315652	0.4849439230
H	-5.1701770355	1.2819745388	0.7744022683
C	-2.0156066979	1.7165310558	2.1579441423
H	-1.7240049326	2.5509068526	1.5066097827
H	-2.3682607164	2.1504381697	3.1072024585
H	-1.1170161010	1.1118162068	2.3612170279
C	-3.5877685867	-0.1330940912	2.6253956780
H	-4.4687807410	-0.7165034545	2.3300803922
H	-2.7795556102	-0.8314867477	2.8923076111
H	-3.8545056149	0.4166526760	3.5415574766
C	-5.0985679135	-0.9895246411	-0.5043575543
H	-5.4957749970	-0.8321688473	0.5064872775
H	-5.2512254485	-0.0700976409	-1.0873287021
H	-5.7109749594	-1.7760684037	-0.9731744595
C	-3.2659124536	-1.8302151671	-1.9432055541
H	-3.7458216133	-2.7902254818	-2.1877897892
H	-3.6344136876	-1.0941189904	-2.6709386428
H	-2.1856053512	-1.9536172463	-2.1113604418
C	-3.5083974347	-2.6876883365	0.3938335389

H	-4.2419037002	-3.4372880321	0.0575752629
H	-2.5192399043	-3.1569694764	0.3329434313
H	-3.7224874607	-2.4776287359	1.4484415925
C	2.3467647867	0.3727480524	2.9565442816
H	1.6423767724	1.2153673069	3.0387295242
H	1.7578076913	-0.5476341645	2.9014756753
H	2.9426592363	0.3530769835	3.8828862144
C	4.1446273776	1.7662102197	1.9701892037
H	4.7860076182	2.0063241128	1.1143709892
H	3.5514497006	2.6556967530	2.2211691320
H	4.8067503299	1.5780017194	2.8303697625
C	4.2071066795	-0.6972665634	1.6598077970
H	3.6636408593	-1.6120446645	1.3979064297
H	5.0083510384	-0.5441883950	0.9236398214
H	4.6894735976	-0.8605554542	2.6366026027
C	3.5442923379	-0.8904398572	-1.6732063554
H	2.6686050430	-1.5389624523	-1.7772319854
H	4.0896615157	-0.9214647364	-2.6296668605
H	4.2022682217	-1.3112763920	-0.9037759790
C	2.1698742236	0.9958685566	-2.4941442219
H	1.2362102965	0.4125631121	-2.4685305643
H	1.9162068861	2.0640097434	-2.4323429823
H	2.6495555007	0.8297901414	-3.4715016539
C	4.3756663895	1.4439683752	-1.4058545625
H	4.8043609089	1.4291761405	-2.4205589500
H	4.1592129416	2.4949837977	-1.1653954249
H	5.1558103801	1.0831686340	-0.7225232945

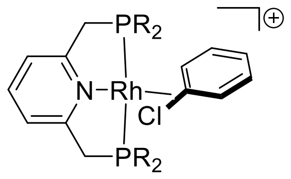

(tBuL1)Rh-SC-2

R = tBu

82

(tBuL1)Rh-SC-2

Rh	0.0119317143	-0.1862918546	0.1279565347
P	-2.2196424778	-0.7669086223	0.2241101057
P	2.3317617119	-0.1951739314	0.0525102904
N	0.2371874127	-2.0260941417	-0.7310797279
C	-1.9854864789	-2.5959384776	0.1079033251
C	-0.7589189898	-2.9451442078	-0.6665293424
C	-0.6286117622	-4.2032983143	-1.2509550149
C	0.5554057311	-4.5500416361	-1.8870982974
C	1.5850544958	-3.6186811467	-1.9209259008
C	1.4013470916	-2.3643744466	-1.3448478351
C	2.4771987430	-1.3333162554	-1.3987083385
C	-3.1388821237	-0.5901972597	1.8653494547
C	-3.2910943617	-0.3337852568	-1.2673277198
C	3.0573665335	-1.1659394998	1.5138291849

C	3.4338505969	1.2660478427	-0.4303339285
H	-1.8330973841	-2.9345745602	1.1459874683
H	-2.8661000517	-3.1334345249	-0.2723602580
H	-1.4581038654	-4.9085822980	-1.1830868475
H	0.6772013012	-5.5346794238	-2.3429952748
H	2.5367629140	-3.8481909735	-2.4020080434
H	3.4670968387	-1.7927220950	-1.5253909835
H	2.2969365328	-0.6967008131	-2.2785900964
H	-0.6346987839	4.6864310169	1.7075182037
C	-0.5736507443	4.5252271449	0.6298901286
C	-0.4107342821	3.2462957092	0.1105932089
Cl	-0.2983591944	1.9036540093	1.2423821085
C	-0.3244106058	2.9950323912	-1.2523894476
H	-0.2008310880	1.9638512800	-1.5956562404
C	-0.4049471879	4.0747045586	-2.1304631968
H	-0.3401099470	3.8982250992	-3.2064203677
C	-0.5681111349	5.3693859961	-1.6396495443
H	-0.6303412037	6.2114780910	-2.3317168012
C	-0.6525671997	5.5911296931	-0.2649870264
H	-0.7804107629	6.6047502079	0.1207434234
C	3.0460547348	1.6797281383	-1.8549854318
H	3.4719939022	2.6731505252	-2.0647190973
H	3.4431862692	0.9920408882	-2.6149262764
H	1.9568161666	1.7578246970	-1.9811199042
C	4.9267137711	0.9409596262	-0.4065026028
H	5.4838840354	1.7999939527	-0.8132030420
H	5.3029979710	0.7646992265	0.6095896778
H	5.1763178627	0.0681594312	-1.0280286233
C	3.3390729954	-0.1934064056	2.6616168900
H	4.1793076613	0.4806385323	2.4486864964
H	2.4546002355	0.4158911283	2.9049579677
H	3.6023144847	-0.7683383748	3.5635158600
C	4.3129833763	-1.9639137251	1.1610905992
H	5.1445191904	-1.3389009994	0.8162669176
H	4.6568415084	-2.5014591208	2.0594448125
H	4.1093881315	-2.7251932746	0.3938155414
C	1.9794825962	-2.1524206898	1.9820505590
H	2.3298010767	-2.6502772789	2.9003836999
H	1.0283021835	-1.6399313230	2.1947288532
H	1.7832905626	-2.9370574838	1.2378038047
C	-2.0809190146	-0.7551553221	2.9635656431
H	-1.3228319246	0.0388235128	2.9306951331
H	-2.5740395505	-0.7137089322	3.9477984315
H	-1.5557245825	-1.7205607379	2.9021158130
C	-3.7621896767	0.8030749675	1.9728970985
H	-3.0424776728	1.6007569803	1.7385311449
H	-4.6339345324	0.9149709015	1.3139858529
H	-4.1127790430	0.9618906743	3.0049378768
C	-4.2211588304	-1.6547649639	2.0638134683
H	-4.7164024827	-1.4803756457	3.0324066385
H	-4.9961030438	-1.6222675711	1.2892818808
H	-3.8093600296	-2.6735516623	2.0956820448

C	-3.4301990524	1.1870195303	-1.3646588094
H	-2.4546122106	1.6884667398	-1.3233184562
H	-3.8997137282	1.4460357764	-2.3269970193
H	-4.0591553062	1.6060334398	-0.5701264515
C	-4.6721119163	-0.9845614044	-1.2545306418
H	-5.3170483980	-0.5719431031	-0.4669983387
H	-5.1718997748	-0.7904082713	-2.2171551501
H	-4.6254911833	-2.0766323839	-1.1292039213
C	-2.4990729379	-0.8278245514	-2.4840595961
H	-1.4797211401	-0.4112845011	-2.4950439579
H	-2.4198962864	-1.9242028524	-2.5186989574
H	-3.0133345518	-0.5075152224	-3.4037469817
C	3.1342572388	2.4438857140	0.5023477466
H	3.7895969947	3.2873564387	0.2337912187
H	2.0970558186	2.7834313737	0.3939684866
H	3.3099223328	2.2122837098	1.5597036598

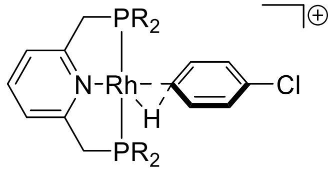

(tBuL1)Rh-H-TS

R = tBu

82

(tBuL1)Rh-H-TS

Rh	0.2439804725	0.0788859077	-0.0578455330
P	-1.2353595347	1.8809526168	-0.2108033022
P	2.1930309825	-1.1873985102	-0.0447149076
N	1.5731342206	1.5485329230	0.6981965080
C	0.0431275241	3.2254575962	-0.1832476279
C	1.2655561614	2.8587086603	0.5917975312
C	2.1023493143	3.8394834948	1.1230021369
C	3.2886167794	3.4619379524	1.7393281618
C	3.6158903903	2.1120651369	1.8065098095
C	2.7349396752	1.1704343930	1.2808369753
C	3.0299910689	-0.2944513712	1.3482357261
C	-2.1415490315	2.2533120444	-1.8262008919
C	-2.2841106129	2.2199708715	1.3189481409
C	3.2479461507	-0.8143126015	-1.5769282642
C	2.2697002522	-3.0106795967	0.4415617828
H	-0.3581166740	4.1963321951	0.1403272870
H	1.8258195671	4.8907112205	1.0315756047
H	3.9604446368	4.2157016463	2.1557985164
H	4.5454042962	1.7805460333	2.2711122536
H	4.1108816961	-0.4808022746	1.4164957481
H	2.5761012636	-0.6977314279	2.2668302981
C	-3.4245259481	-3.0780406702	0.2585928296
C	-2.5504990314	-2.7550716709	1.2971631292
H	-2.7103543037	-3.1733261262	2.2925283082
C	-1.4812634940	-1.8992407009	1.0525705483

C	-1.2672378728	-1.3196841330	-0.2128123097
C	-2.1575180420	-1.6791940148	-1.2353851943
H	-2.0246309055	-1.2875866069	-2.2463923416
C	-3.2271109047	-2.5475276767	-1.0110411014
H	-3.9040296183	-2.8141742574	-1.8244419212
H	-0.1506838468	-0.8810498062	-1.1917712430
Cl	-4.7509307009	-4.1502183964	0.5534588575
H	-0.8225310840	-1.6535324705	1.8888893284
C	2.9329573739	0.6159508665	-2.0329589370
H	3.4567070839	0.8034728897	-2.9836887766
H	1.8537354744	0.7625546374	-2.1909972437
H	3.2820593232	1.3699601224	-1.3138503837
C	2.8370187408	-1.7705640404	-2.6985083390
H	3.1217061213	-2.8104086650	-2.4934138150
H	1.7526322076	-1.7357566871	-2.8863402124
H	3.3437604765	-1.4695603409	-3.6287519916
C	4.7506933415	-0.9077530896	-1.3082883005
H	5.0735747102	-1.9020697231	-0.9811633511
H	5.2915250629	-0.6764398113	-2.2397380990
H	5.0784821535	-0.1717541264	-0.5595046002
C	3.6595037610	-3.6317827518	0.3028819712
H	3.6265263939	-4.6565165632	0.7053855332
H	3.9872896417	-3.7069405816	-0.7421712921
H	4.4244738312	-3.0837403825	0.8728732217
C	1.8402486692	-3.1126750321	1.9097684883
H	1.6422886129	-4.1694462534	2.1462744990
H	2.6236992209	-2.7687573985	2.6000925212
H	0.9166574289	-2.5554124775	2.1141778424
C	1.2583386486	-3.7778703447	-0.4187915478
H	1.2680887905	-4.8369636828	-0.1171520390
H	0.2374475365	-3.3989563217	-0.2806165359
H	1.4968392994	-3.7403125629	-1.4888301933
H	0.3517052549	3.3497450379	-1.2350047163
C	-1.2622637943	1.6790570631	-2.9422162240
H	-1.1690003810	0.5879172562	-2.8706415965
H	-1.7099739382	1.9234581091	-3.9184024195
H	-0.2446267162	2.0992652282	-2.9335448565
C	-3.5077041793	1.5652332695	-1.8421625470
H	-3.4498882430	0.5064556086	-1.5565718172
H	-4.2234779451	2.0646489948	-1.1754921170
H	-3.9221739317	1.6176709859	-2.8612753673
C	-2.3280080077	3.7542415598	-2.0696199276
H	-2.8817996171	3.8882060085	-3.0123543959
H	-2.9074297668	4.2456429070	-1.2787039464
H	-1.3742258323	4.2880164236	-2.1827052350
C	-3.3178872399	1.1044810183	1.4993281911
H	-2.8525384751	0.1111897301	1.5281218863
H	-3.8417110540	1.2608041281	2.4555700941
H	-4.0765702901	1.0996625886	0.7080402801
C	-2.9864190328	3.5759190810	1.2929063088
H	-3.7747015803	3.6165194130	0.5297146707
H	-3.4700344681	3.7469114801	2.2678151454

H	-2.2945354156	4.4143435429	1.1238919403
C	-1.3056804801	2.1711521045	2.4991281597
H	-0.7424554112	1.2249443080	2.5209744743
H	-0.5845343724	3.0015342172	2.4863317654
H	-1.8738479111	2.2461706196	3.4393884147

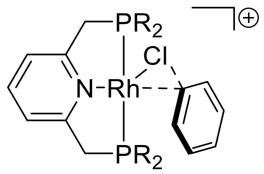

(tBuL1)Rh-Cl-TS

R = tBu

82

(tBuL1)Rh-Cl-TS

Rh	0.0607256832	0.1520513017	0.2091639656
P	2.4221788399	-0.0915411855	0.0522723667
P	-2.1867172528	-0.5158026977	0.3711855885
N	0.1794999273	-1.5583228761	-0.9756690994
C	2.4031851263	-0.8441903965	-1.6405530538
C	1.2677599518	-1.8026833362	-1.7436340347
C	1.3442598600	-2.9318441331	-2.5567587346
C	0.2983056762	-3.8458204713	-2.5622025840
C	-0.7709526176	-3.6373417644	-1.6991027561
C	-0.7907627353	-2.5016871377	-0.8945398426
C	-1.8077562852	-2.3137459922	0.1728150213
C	3.8374683306	1.1923067598	-0.0790250489
C	2.9105380270	-1.4588081955	1.2864595657
C	-3.3970673825	-0.2257131957	-1.0833049301
C	-3.0437003965	-0.4812545207	2.0547979884
H	2.2263155911	0.0044010643	-2.3203211194
H	3.3495175668	-1.3172901554	-1.9391271560
H	2.2300883136	-3.0848838628	-3.1745451787
H	0.3330291506	-4.7286377273	-3.2039245835
H	-1.5807998212	-4.3637179129	-1.6189326816
H	-2.6887996304	-2.9467254406	0.0193696018
H	-1.3403900672	-2.6269352057	1.1197288564
H	0.8793466127	3.9934631881	-2.7527785208
C	0.1374730304	3.6542146131	-2.0255394012
C	0.5390094988	2.8009437904	-0.9978159088
H	1.5780067478	2.5194591963	-0.8918466625
C	-0.4054256978	2.3163444367	-0.0926325222
Cl	0.1569400372	1.8940233159	1.7789769225
C	-1.7097205736	2.8268820678	-0.1092076151
H	-2.4078320448	2.5524982581	0.6777288605
C	-2.0900341390	3.6985292197	-1.1224309287
H	-3.1137319609	4.0805840243	-1.1374670627
C	-1.1796234940	4.0987869845	-2.1030863486
H	-1.4863783807	4.7855718208	-2.8938443897
C	-4.4268361894	0.8655378510	-0.7834691971
H	-3.9697160205	1.8164870115	-0.4946394141

H	-5.0099268992	1.0523844967	-1.6989750518
H	-5.1380022433	0.5637563847	-0.0028692238
C	-4.1634478932	-1.5003826703	-1.4548678505
H	-4.7353700623	-1.9227679296	-0.6169686960
H	-4.8866165549	-1.2476846768	-2.2462107988
H	-3.5076163941	-2.2799922096	-1.8665247456
C	-2.5391494450	0.1880469439	-2.2856098940
H	-1.9759100019	1.1103704955	-2.0983283799
H	-1.8232954536	-0.5972786442	-2.5672500067
H	-3.1986245614	0.3574622070	-3.1516543358
C	-3.3953454127	0.9554326762	2.4519158468
H	-2.4939738764	1.5782127358	2.5353318043
H	-4.1015373624	1.4327094832	1.7614227059
H	-3.8733820306	0.9389013305	3.4439235949
C	-4.2955203847	-1.3583000630	2.0792005628
H	-4.7108021313	-1.3630159498	3.0994623077
H	-5.0814297776	-0.9852987579	1.4097216243
H	-4.0810969548	-2.4036651750	1.8133937212
C	-2.0274003351	-1.0060526314	3.0771719507
H	-1.0729377257	-0.4633064358	3.0134864020
H	-2.4354629152	-0.8582053443	4.0890835749
H	-1.8293634054	-2.0817084604	2.9685087706
C	1.7110338118	-2.3862167897	1.5231107924
H	1.9601481532	-3.0599168443	2.3579001074
H	0.8066980851	-1.8203760344	1.7950326385
H	1.4883629525	-3.0162492268	0.6514826724
C	3.2082807076	-0.7725449392	2.6247951669
H	4.1054729844	-0.1429149331	2.6033724928
H	2.3590172759	-0.1508400024	2.9490855878
H	3.3692099791	-1.5433722671	3.3946181714
C	4.0713705893	-2.3388253038	0.8194670640
H	5.0215813563	-1.8063654686	0.7245278018
H	4.2208262767	-3.1441427820	1.5563672161
H	3.8470524717	-2.8249522048	-0.1422854311
C	3.5793195454	2.3034156478	0.9461451442
H	4.3909522479	3.0450643312	0.8820160429
H	2.6294929465	2.8266547891	0.7829953974
H	3.5626931575	1.9192837919	1.9747405553
C	5.2309309252	0.6055090318	0.1668872269
H	5.9669693146	1.4090926212	0.0068042482
H	5.3772196329	0.2381658695	1.1890744364
H	5.4757616231	-0.1998453336	-0.5395014761
C	3.8902063740	1.7736629906	-1.5015160667
H	4.3153180741	1.0535240583	-2.2145127572
H	2.9293858319	2.1115616897	-1.9071671141
H	4.5593277653	2.6479919769	-1.4896671904

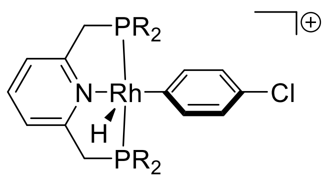

(tBuL1)Rh(H)(Ar)

R = tBu

82

(tBuL1)Rh(H)(Ar)

H	0.2462941717	0.0411290268	1.4904999813
Rh	0.2679462766	-0.0467779998	-0.0037730051
P	1.1877020692	2.0876510955	-0.0644066060
P	-0.0684479498	-2.3277808093	0.1557745546
N	2.3395895361	-0.6193448397	-0.0763069223
C	2.8173703521	1.6465483832	-0.8418698334
C	3.2733718941	0.2634288810	-0.4802566884
C	4.6126690353	-0.1114663839	-0.5871290635
C	4.9778902966	-1.4175663426	-0.2829638277
C	4.0045299126	-2.3135836825	0.1486507299
C	2.6852945967	-1.8799335717	0.2608037354
C	1.6097995514	-2.7589559105	0.8218770386
C	0.4648384762	3.3323321690	-1.2775959408
C	1.6431142632	2.8243053606	1.6077524377
C	-0.1005234191	-3.1198356486	-1.5636062417
C	-1.2633254930	-3.1003898626	1.3851882066
H	2.6480368060	1.6847127695	-1.9308663843
H	3.6024183775	2.3872126686	-0.6288477398
H	5.3547949728	0.6167771324	-0.9179016777
H	6.0191094059	-1.7348589924	-0.3727702891
H	4.2635340969	-3.3390640002	0.4161550040
H	1.8646175495	-3.8216552357	0.7198894385
H	1.5474057726	-2.5492238599	1.9021167660
H	-4.2805430220	1.6421436570	1.9265502371
C	-3.7506322008	1.2594261500	1.0522424308
C	-2.3912205711	0.9527557879	1.1221534568
H	-1.8882066910	1.1084465138	2.0781697306
C	-1.6800689097	0.4552436690	0.0182961059
C	-2.4133159292	0.2741336216	-1.1678424741
H	-1.9270098393	-0.1284310549	-2.0630540941
C	-3.7705476368	0.5798756960	-1.2641890048
H	-4.3138426384	0.4305105128	-2.1991079319
C	-4.4366321820	1.0733769056	-0.1450762073
Cl	-6.1268965667	1.4496788165	-0.2430109517
C	0.6680465579	-4.4396232706	-1.6289815618
H	0.2846588648	-5.1889260821	-0.9234871559
H	0.5682397450	-4.8617297132	-2.6419167355
H	1.7434246218	-4.3044382160	-1.4436906509
C	-1.5407735224	-3.3332932440	-2.0323258738
H	-2.0311805497	-4.1527968601	-1.4910034318
H	-2.1570818734	-2.4294826826	-1.9245463345
H	-1.5291872378	-3.6079846164	-3.0989241560

C	0.5781708018	-2.1035096705	-2.4942370733
H	0.5207654687	-2.4636873528	-3.5338384007
H	0.0776145214	-1.1192471188	-2.4695829036
H	1.6405557769	-1.9592719989	-2.2543643853
C	-0.9743484922	-2.4522388172	2.7443285899
H	-1.0681531710	-1.3577766538	2.6966626363
H	-1.7157006677	-2.8186489754	3.4708512724
H	0.0172329503	-2.7065329774	3.1448941924
C	-2.7097722421	-2.7622204699	1.0057922252
H	-3.3685579297	-3.1033176617	1.8195226397
H	-2.8612805229	-1.6818334300	0.8812334007
H	-3.0380227043	-3.2661186926	0.0906808544
C	-1.0761574183	-4.6153017637	1.4766042521
H	-1.7411151085	-5.0136148035	2.2589397054
H	-1.3416217671	-5.1209117786	0.5381539386
H	-0.0483919406	-4.8999030376	1.7473420433
C	0.0309786588	2.5060850812	-2.4940756701
H	-0.7474043928	1.7811297886	-2.2256281311
H	-0.3932564255	3.1811900432	-3.2535683858
H	0.8682216148	1.9739043060	-2.9728848794
C	1.4592365306	4.4018159638	-1.7340570338
H	0.9713417002	5.0282430524	-2.4975343035
H	1.7771516647	5.0665001118	-0.9231215330
H	2.3576690027	3.9711713657	-2.2000647577
C	-0.7739888263	3.9760188955	-0.6502104720
H	-1.4895789201	3.2210226310	-0.2916476212
H	-0.5166333945	4.6448367465	0.1830024665
H	-1.2851558001	4.5863633749	-1.4109629664
C	0.3887436172	2.8859509137	2.4823511728
H	-0.0050065802	1.8793688586	2.6788495437
H	0.6488954133	3.3397965207	3.4513269921
H	-0.4169640187	3.4832302905	2.0367335332
C	2.2724197256	4.2118669569	1.4956882275
H	1.5515958545	4.9669349794	1.1560348009
H	2.6264870739	4.5232486637	2.4909835228
H	3.1411582690	4.2294198655	0.8213035772
C	2.6464654450	1.8694081720	2.2646691793
H	2.2622533502	0.8404670956	2.3213136153
H	3.6154316054	1.8501716135	1.7454918180
H	2.8366483473	2.2122040861	3.2936486024

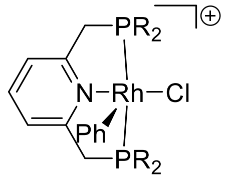

(tBuL1)Rh(Cl)(Ph)

R = tBu

82

(tBuL1)Rh(Cl)(Ph)

Rh	-0.0035338822	0.1583816586	0.3198111972

P	2.3728032790	0.3532097464	0.1535193309
P	-2.3111370392	0.3614584381	0.1024085415
N	0.0086810693	0.5683147518	-1.7068877019
C	2.3667016903	1.2226323497	-1.4805985713
C	1.1529658250	0.9607730816	-2.3150043004
C	1.1795277540	1.2277844020	-3.6838761503
C	0.0075367419	1.1545886906	-4.4229830554
C	-1.1743087087	0.8201623007	-3.7720132439
C	-1.1500973239	0.5182393730	-2.4136245363
C	-2.3937818185	0.0717623174	-1.7143596047
C	3.1439609703	1.6512202508	1.2936262508
C	-2.6645022557	2.1994658551	0.3654675868
C	-3.6379433225	-0.7564758338	0.8425501301
H	2.1213734506	1.5197095936	-4.1504544693
H	0.0105708692	1.3713317059	-5.4932791725
H	-2.1203994716	0.7721669393	-4.3127168676
H	-3.2919545735	0.4857010819	-2.1922515306
H	-2.4520309119	-1.0245687640	-1.8091682096
H	-0.3883401032	-4.6616332489	1.8955957972
C	-0.3621858435	-4.0307283182	1.0036571618
C	-0.5001157452	-4.5982051092	-0.2611383314
H	-0.6362555859	-5.6754534550	-0.3751810458
C	-0.4533900140	-3.7696844026	-1.3778951260
H	-0.5444470006	-4.1920577472	-2.3817811863
C	-0.2820522252	-2.3900758832	-1.2347447484
H	-0.2255163544	-1.7918061578	-2.1431944627
C	-0.1604796894	-1.8129380174	0.0355122642
C	-0.1948641578	-2.6534708104	1.1578008054
H	-0.0945065050	-2.2361368783	2.1583388690
H	3.2868775263	1.0524767218	-2.0576932179
H	2.3563744192	2.2995434189	-1.2406782275
Cl	-0.0548575210	0.1980286284	2.6588798019
C	-1.3717649698	2.9386767658	-0.0052488341
H	-1.4974644646	4.0112239268	0.2130189151
H	-0.5142994612	2.6012461194	0.6023140450
H	-1.1174621298	2.8483335135	-1.0701744699
C	-3.7956048773	2.7369581020	-0.5105762083
H	-3.5750207240	2.6294771241	-1.5829454472
H	-4.7632805509	2.2643282375	-0.3060101866
H	-3.9103532155	3.8149824378	-0.3135038609
C	-2.9453390868	2.4519189213	1.8492968071
H	-3.9041960846	2.0277181021	2.1728165470
H	-2.1476750445	2.0427655882	2.4874323779
H	-2.9944672284	3.5389668060	2.0195263119
C	-5.0366628353	-0.1710585921	0.6387376463
H	-5.2658047499	0.0029039674	-0.4233152353
H	-5.7726894098	-0.8991615548	1.0138247723
H	-5.1947995258	0.7630572579	1.1918348933
C	-3.3391942504	-0.9417454908	2.3322855749
H	-4.1009228582	-1.6112946392	2.7610367854
H	-2.3505556207	-1.3891684541	2.4913372908
H	-3.3676412535	0.0006824145	2.8918909990

C	-3.5930679435	-2.1259122776	0.1539971029
H	-3.9476355109	-2.0848579698	-0.8859236228
H	-2.5987510535	-2.5865722211	0.1761971096
H	-4.2790708514	-2.7951630994	0.6956263153
C	2.0796122691	2.7304851394	1.5202962599
H	1.7633682608	3.2167687175	0.5835846856
H	1.2002432572	2.3280245190	2.0408362507
H	2.5098302330	3.5211649356	2.1545721448
C	4.3831235332	2.3247131533	0.6969668238
H	4.1750486335	2.8340932669	-0.2555437302
H	4.7228921593	3.1000757548	1.4018727564
H	5.2206379469	1.6373726240	0.5432090748
C	3.4831518263	0.9957716506	2.6345764429
H	2.6190488465	0.4562616442	3.0481400826
H	4.3344103154	0.3061338342	2.5555735680
H	3.7659396197	1.7805295345	3.3535165108
C	4.9715397689	-0.7803106592	-0.1974060275
H	5.5320960078	-1.6945883056	-0.4484979079
H	5.1740217144	-0.0494056123	-0.9948022709
H	5.3833541247	-0.3980285916	0.7450156880
C	3.4915566814	-1.1457396036	-0.0877701618
C	3.2744299319	-2.1043610452	1.0862361270
H	3.8944710156	-3.0012801411	0.9318061872
H	3.5622454220	-1.6628389252	2.0486613674
H	2.2281851471	-2.4251205182	1.1551289816
C	3.0668029771	-1.8302849608	-1.3899151003
H	3.2573911070	-1.2066697240	-2.2757237233
H	3.6620666620	-2.7489592044	-1.5090245411
H	2.0117047290	-2.1249035207	-1.3804210255

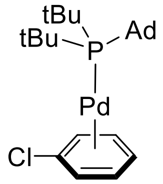

Pd-SC

65

Pd-SC

P	-0.573296	-1.209615	0.085261
C	-1.453366	-2.656565	-0.835083
C	-0.350596	-1.717356	1.933760
C	-2.625254	-3.319613	-0.091527
H	-3.071454	-4.087525	-0.735860
H	-2.306810	-3.817190	0.825920
H	-3.412373	-2.608303	0.164590
C	-1.956179	-2.153372	-2.202506
H	-1.157768	-1.654212	-2.758570
H	-2.284516	-3.017893	-2.791595
H	-2.806115	-1.475099	-2.122607
C	-0.381055	-3.720424	-1.161899
H	-0.828100	-4.486297	-1.807752

H	0.459826	-3.265013	-1.694036
H	0.012329	-4.223046	-0.280774
C	0.735098	-0.804988	2.550948
H	0.441153	0.240150	2.616296
H	0.948092	-1.155132	3.568823
H	1.656431	-0.853606	1.963710
C	-1.620448	-1.672033	2.800975
H	-2.012732	-0.660059	2.912361
H	-2.418558	-2.301411	2.404055
H	-1.381241	-2.037861	3.807376
C	0.237954	-3.140460	1.995005
H	-0.477692	-3.912615	1.710638
H	1.125395	-3.228936	1.361907
H	0.542674	-3.346284	3.027811
Pd	1.498802	-0.682333	-0.798858
C	-1.698798	0.345314	0.009539
C	-1.193695	1.378442	1.050398
C	-3.209880	0.115027	0.241046
C	-1.495395	1.006022	-1.384779
H	-1.321451	0.994228	2.063507
H	-0.125097	1.557892	0.895479
C	-1.969549	2.705075	0.947469
H	-3.384945	-0.331374	1.224115
H	-3.610660	-0.580632	-0.500660
C	-3.982632	1.447384	0.135487
H	-0.424558	1.188422	-1.529587
H	-1.809606	0.337717	-2.185493
C	-2.286068	2.322699	-1.497253
H	-1.584120	3.390456	1.712144
C	-3.464120	2.436154	1.193747
C	-1.775434	3.319055	-0.446411
H	-5.048377	1.249040	0.304688
C	-3.782743	2.046643	-1.267623
H	-2.137149	2.733859	-2.503416
H	-4.031851	3.373884	1.143645
H	-3.612830	2.022970	2.199984
H	-0.714189	3.535163	-0.615580
H	-2.320126	4.269019	-0.522340
H	-4.160105	1.352663	-2.030005
H	-4.358111	2.975974	-1.365412
C	3.020908	1.810569	0.124973
C	3.817825	1.238750	1.105644
C	4.444967	0.010406	0.843596
C	4.301976	-0.609529	-0.393734
C	3.532742	0.000257	-1.414916
C	2.852228	1.215142	-1.140392
H	3.578397	-0.376493	-2.433364
H	2.341969	1.759106	-1.926082
H	5.051622	-0.451683	1.615871
H	4.813913	-1.543637	-0.599810
H	3.935980	1.730688	2.064078
Cl	2.197436	3.330530	0.453278

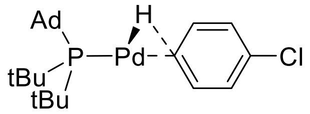

Pd-H-TS

65

Pd-H-TS

Pd	-1.0993756209	-0.2935143054	-0.1158158061
P	1.1627196404	-0.9600547194	0.0329523457
C	1.2924697740	-1.8262967119	1.7369335329
C	1.6061333938	-2.2165977674	-1.3369710510
C	2.4985062415	-2.7434330229	1.9371206703
C	1.2819657009	-0.7745127139	2.8521790715
C	-0.0106713798	-2.6218684569	1.9197521256
C	1.0497510162	-1.6720690517	-2.6626222576
C	3.0961549188	-2.5287630361	-1.4844559654
C	0.8442785651	-3.5214553760	-1.0811123098
C	2.3714516725	0.5072327217	-0.0249565801
C	2.5368980959	0.9781213713	-1.4844032759
C	3.7681666195	0.2469223553	0.5643546249
C	1.7072348918	1.6811408117	0.7328594275
C	3.4023681234	2.2405598315	-1.5642420726
C	4.6248116602	1.5175771520	0.4864785463
C	2.5779347377	2.9391499099	0.6688618773
C	4.7826424637	1.9435396829	-0.9737690767
C	2.7415115537	3.3755377262	-0.7851692340
C	3.9508161924	2.6413138929	1.2769338607
H	2.4731116095	-3.1602190176	2.9580403922
H	2.5024262183	-3.5923183924	1.2419890388
H	3.4505437522	-2.2080907077	1.8235894165
H	0.4273241279	-0.0880568856	2.7566349528
H	1.1808658988	-1.2958193224	3.8181395135
H	2.2037435274	-0.1823087170	2.8980996444
H	-0.0406186197	-3.0353439176	2.9415915852
H	-0.8977767118	-1.9704572025	1.8128664940
H	-0.1230684880	-3.4565736508	1.2212972887
H	1.5482008815	-0.7595859369	-3.0037012276
H	1.1871104232	-2.4386101371	-3.4431747118
H	-0.0247455878	-1.4530998554	-2.5738604755
H	3.6803212069	-1.6508018483	-1.7897931143
H	3.5377055027	-2.9245844836	-0.5606143024
H	3.2289565662	-3.2931137154	-2.2683056598
H	1.2195978420	-4.0821902104	-0.2159797496
H	-0.2330738775	-3.3390379100	-0.9488955592
H	0.9624608713	-4.1704220868	-1.9638731143
H	3.0168075684	0.1980532945	-2.0907277127
H	1.5423764205	1.1708436298	-1.9193852937
H	4.2688530610	-0.5701786798	0.0238568715
H	3.6926108285	-0.0673086547	1.6151081826
H	0.7157359406	1.8724487218	0.2904028573
H	1.5342295455	1.4250797364	1.7837354725

H	3.5045210552	2.5242649943	-2.6247827706
H	5.6154842854	1.3009749249	0.9196400013
H	2.0811245582	3.7370129329	1.2450635416
H	5.4264309120	2.8364921603	-1.0426723424
H	5.2802088670	1.1449663728	-1.5504746725
H	1.7578248690	3.6147059486	-1.2221381549
H	3.3546651392	4.2905314144	-0.8479691367
H	3.8421472303	2.3483770619	2.3353745237
H	4.5805390966	3.5468097879	1.2596387825
C	-3.0066083046	0.2909624138	-0.2269963021
C	-3.9538740116	-0.4598634454	-0.9442907437
C	-3.4882862731	1.3189333410	0.6019650746
C	-5.3235050040	-0.2419920516	-0.7984070419
C	-4.8537710563	1.5524136799	0.7611358566
C	-5.7614308484	0.7630837688	0.0598965913
Cl	-7.4712590119	1.0479428354	0.2477288082
H	-2.7840533921	1.9537369262	1.1468633494
H	-5.2167385767	2.3444931300	1.4188853355
H	-1.3815783432	0.7338954308	-1.2122115707
H	-3.6214802077	-1.2427896215	-1.6314818701
H	-6.0506857014	-0.8414294997	-1.3494729319

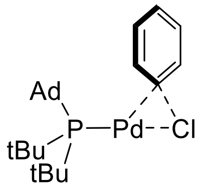

Pd-Cl-TS

65

Pd-Cl-TS

Pd	-1.7581152269	0.7883025392	-0.6802556199
P	0.4085517025	1.4312572890	-0.2251936955
C	1.1132016587	2.4197956493	-1.7115488610
C	0.4143830649	2.5765624331	1.3146291264
C	2.3485435018	3.2721749339	-1.4169549401
C	1.4373955026	1.4459977473	-2.8495663106
C	-0.0105085879	3.3179320446	-2.2519318479
C	-0.5619230361	1.9834355256	2.3413548169
C	1.7811344900	2.8025217054	1.9640911198
C	-0.1826053397	3.9353771983	0.9333818449
C	1.5467822864	-0.0623854609	0.1156462076
C	1.2143062712	-0.6478833968	1.5044717546
C	3.0578600166	0.2228338358	0.0500476314
C	1.2016145426	-1.1695351737	-0.9067592768
C	2.0296867055	-1.9165626664	1.7793715488
C	3.8607797578	-1.0551151033	0.3237705042
C	2.0223984345	-2.4367454393	-0.6455926935
C	3.5217812731	-1.5861711463	1.7175448300
C	1.6959073957	-2.9849595848	0.7416541287
C	3.5148158609	-2.1100554737	-0.7292132024
H	2.6863081055	3.7505340274	-2.3515953116

H	2.1398183732	4.0779357815	-0.7019870566
H	3.1877914157	2.6819902344	-1.0278570714
H	0.5821225185	0.7888785220	-3.0683727404
H	1.6501379959	2.0319872948	-3.7585535629
H	2.3188799514	0.8244840160	-2.6493254308
H	0.3387572063	3.8005929831	-3.1800276211
H	-0.9068466064	2.7231006211	-2.4896317583
H	-0.3090597771	4.1109863182	-1.5593217906
H	-0.2805849566	0.9884358259	2.6991223833
H	-0.6095438543	2.6567543291	3.2136631645
H	-1.5687975742	1.9052237539	1.9061159778
H	2.2037457387	1.8817163017	2.3853492049
H	2.5145816497	3.2239248678	1.2649288767
H	1.6693446852	3.5174410063	2.7965296512
H	0.4769213601	4.5358661143	0.2948256623
H	-1.1545600446	3.8175136536	0.4309435285
H	-0.3522462948	4.5108757615	1.8580607617
H	1.4417949719	0.0790509206	2.2951585870
H	0.1369427522	-0.8742278266	1.5610564320
H	3.3412785142	0.9967892345	0.7778706481
H	3.3354010303	0.6026783844	-0.9436372977
H	0.1253373216	-1.3897923330	-0.8374364532
H	1.3894348133	-0.8328947418	-1.9321857491
H	1.7693203276	-2.2815199911	2.7869076370
H	4.9353562146	-0.8129743284	0.2688189017
H	1.7611346771	-3.1838497190	-1.4136445694
H	4.1211908776	-2.4852037271	1.9399642866
H	3.7784821621	-0.8328800531	2.4824235116
H	0.6284610308	-3.2453260715	0.8042274184
H	2.2726838687	-3.9048549275	0.9392808667
H	3.7678276888	-1.7359068204	-1.7362317498
H	4.1149624844	-3.0213619940	-0.5661629891
C	-2.0419170224	-3.1637334471	0.3313610834
C	-2.0831835150	-3.0585061350	1.7232109852
C	-2.5322690586	-1.8711678592	2.3064863907
C	-2.9283555569	-0.7940796157	1.5205138440
C	-2.8094013680	-0.8868986724	0.1229505099
C	-2.4200141440	-2.0968894463	-0.4776481981
H	-1.7167632930	-4.0955393188	-0.1386286696
H	-1.7888847395	-3.9033578972	2.3489710306
H	-2.5907581663	-1.7834771493	3.3944373749
H	-3.3112329244	0.1215565724	1.9724836763
Cl	-4.0374921537	0.1851032054	-0.9210280296
H	-2.4099759601	-2.1827061115	-1.5651264112

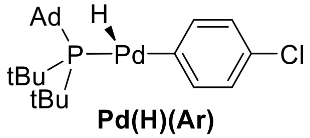

Pd(H)(Ar)

65
Pd(H)(Ar)

P	-1.1668090118	-0.9460551265	-0.0228253396
C	-1.2780992096	-1.8104938472	-1.7287012077
C	-1.6062017212	-2.2073323846	1.3430138969
C	-2.4686461937	-2.7460511512	-1.9343775900
H	-2.4337140213	-3.1609873496	-2.9557608554
H	-2.4626965254	-3.5954345404	-1.2400475432
H	-3.4285507305	-2.2243255406	-1.8233969390
C	-1.2785676609	-0.7607134627	-2.8458444973
H	-0.4347132782	-0.0613098920	-2.7493977634
H	-1.1660237631	-1.2827819559	-3.8101530584
H	-2.2089544497	-0.1825864066	-2.8969397033
C	0.0397938720	-2.5841015849	-1.9024685612
H	0.0787015525	-3.0095262742	-2.9191015264
H	0.9157362559	-1.9138640112	-1.8103296738
H	0.1705220158	-3.4069770809	-1.1932924471
C	-1.0606661957	-1.6600462554	2.6720265309
H	-1.5642135585	-0.7491738898	3.0097382135
H	-1.2008262373	-2.4268160671	3.4517705555
H	0.0136496706	-1.4385734497	2.5908461502
C	-3.0947759482	-2.5299864814	1.4822440307
H	-3.6862952744	-1.6559214565	1.7847342228
H	-3.5287899990	-2.9286450331	0.5560213605
H	-3.2263151769	-3.2953353880	2.2652897030
C	-0.8322805535	-3.5058724724	1.0908579702
H	-1.1933602705	-4.0670907370	0.2200750624
H	0.2450969935	-3.3149752543	0.9716417706
H	-0.9549882737	-4.1583294249	1.9703854199
C	-2.3821119628	0.5141508594	0.0305528557
C	-2.5550401483	0.9854311452	1.4890170950
C	-3.7762159641	0.2449220293	-0.5619864786
C	-1.7225496794	1.6901546323	-0.7281786490
H	-3.0330371121	0.2030967049	2.0940558985
H	-1.5637002797	1.1845681380	1.9280578857
C	-3.4271690676	2.2434306066	1.5647228380
H	-4.2740440022	-0.5737260991	-0.0211705203
H	-3.6965717207	-0.0708606453	-1.6120333315
C	-4.6396674745	1.5112748108	-0.4883262378
H	-0.7321192918	1.8873727559	-0.2867048853
H	-1.5485568350	1.4334244941	-1.7786423607
C	-2.5999206980	2.9436904727	-0.6669960388
H	-3.5336491202	2.5278377852	2.6246262725
C	-4.8041474994	1.9384907207	0.9708090468
C	-2.7695441552	3.3806589128	0.7861578406
H	-5.6278705819	1.2886814837	-0.9240507841
C	-3.9695789490	2.6377000993	-1.2782683005
H	-2.1058794394	3.7435503091	-1.2427254560
H	-5.4526926762	2.8282131356	1.0364585236
H	-5.2993469140	1.1382935936	1.5473014272
H	-1.7882647442	3.6250876929	1.2255976193
H	-3.3871788406	4.2927665831	0.8463887303
H	-3.8566797833	2.3443651140	-2.3361476706
H	-4.6042980505	3.5397304972	-1.2635618865

Pd	1.0985541926	-0.2982358401	0.1083118701
C	3.0213555659	0.2447656645	0.1825914049
C	3.9658110260	-0.5125734934	0.8976284453
C	3.5127296847	1.2822212466	-0.6294349336
C	5.3367931255	-0.2856567402	0.7752418219
C	4.8792386396	1.5265247764	-0.7661663290
H	2.8154776021	1.9208320473	-1.1795699836
C	5.7813915343	0.7337376498	-0.0621294178
H	5.2470596027	2.3287993558	-1.4088724836
H	1.1393729445	0.7146150445	1.2387904183
H	3.6318817676	-1.3092987225	1.5687069682
Cl	7.4929863827	1.0301869675	-0.2221396694
H	6.0595288134	-0.8892526191	1.3278151013

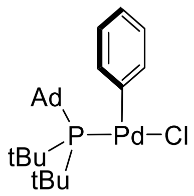

Pd(Cl)(Ph)

65

Pd(Cl)(Ph)

P	0.0327013457	1.1515446064	-0.1564711810
C	0.2481073655	2.5417536509	1.1481624947
C	0.1060136962	1.9387670164	-1.9006739471
C	-0.5286645509	3.8288943958	0.8729374088
H	-0.3535994740	4.5346249559	1.7019855373
H	-0.2101599356	4.3302185570	-0.0492270517
H	-1.6108479296	3.6553988097	0.8111734212
C	-0.1412540407	2.0170933272	2.5343389471
H	0.3832106602	1.0809855323	2.7764863492
H	0.1592047474	2.7684255906	3.2824474191
H	-1.2208608823	1.8593992724	2.6465147298
C	1.7561668058	2.8396716398	1.2271815458
H	1.9339986016	3.5759999978	2.0284747961
H	2.3413184768	1.9435161762	1.5187659764
H	2.1816426801	3.2475382237	0.3062889631
C	0.4545840399	0.8352389247	-2.9079319655
H	-0.2720474722	0.0175029268	-2.9421219498
H	0.5052259936	1.2831022948	-3.9137958269
H	1.4413205746	0.4098210813	-2.6775786003
C	-1.1739730065	2.6565504453	-2.3326596505
H	-2.0207373818	1.9698846067	-2.4548991190
H	-1.4737039438	3.4457186313	-1.6317330022
H	-0.9994577353	3.1323885093	-3.3116412374
C	1.2773189315	2.9255818396	-1.9715954669
H	1.1214323418	3.8326280111	-1.3764833383
H	2.2229866287	2.4537592030	-1.6651450260
H	1.3951929999	3.2409162314	-3.0206392484
C	-1.6366344214	0.2795650765	0.0897722709
C	-1.9362609398	-0.5956844323	-1.1460898919

C	-2.8295288092	1.2249666239	0.3248152530
C	-1.5175537024	-0.6748475825	1.2986486747
H	-2.0552547052	0.0294320115	-2.0412249115
H	-1.0960163319	-1.2828781308	-1.3271271196
C	-3.2262583922	-1.3977186232	-0.9496835165
H	-2.9525162778	1.9183670803	-0.5190347753
H	-2.6609956674	1.8382544738	1.2217826008
C	-4.1197557703	0.4136212510	0.5145137552
H	-0.6813677498	-1.3654666875	1.1361179469
H	-1.3009043640	-0.1201288563	2.2174057434
C	-2.8115051877	-1.4699695864	1.4953960631
H	-3.4016890474	-1.9948925446	-1.8595612978
C	-4.3907862213	-0.4309051578	-0.7319192724
C	-3.0788093353	-2.3227407195	0.2566275474
H	-4.9527484185	1.1178548920	0.6760831957
C	-3.9755260696	-0.5058692339	1.7285973133
H	-2.6838821004	-2.1213252311	2.3755259939
H	-5.3346817169	-0.9887762533	-0.6126074747
H	-4.5144213538	0.2208189692	-1.6140743266
H	-2.2450546968	-3.0260818048	0.0947847396
H	-3.9941284897	-2.9233738436	0.3921185926
H	-3.7974886489	0.0919431324	2.6389742643
H	-4.9113566518	-1.0663833647	1.8920363869
Pd	2.0036288991	0.0381496415	0.2429361883
Cl	4.1509323838	-0.6123481378	0.7707417471
C	1.4394849800	-1.7888001933	-0.1666721301
C	1.2726225118	-2.6557027223	0.9186427473
C	1.2676341278	-2.2679258861	-1.4648001111
C	0.8715409664	-3.9744946529	0.7026381958
H	1.4597035952	-2.3068836974	1.9360224095
C	0.8705907706	-3.5923010675	-1.6722174207
H	1.4402377680	-1.6238464811	-2.3260141894
C	0.6559190761	-4.4451867955	-0.5924051314
H	0.7378844792	-4.6405380545	1.5591048019
H	0.7333595063	-3.9549033719	-2.6944645605
H	0.3430649056	-5.4785003409	-0.7590697356

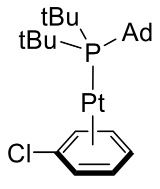

Pt-SC

65

Pt-SC

P	-0.6300453193	1.1291538827	-0.2443132354
C	-1.2247976704	2.6963274317	0.6834368888
C	-0.4364557007	1.5492250554	-2.1069394892
C	-2.3286005367	3.4954916842	-0.0110047577
H	-2.6251884195	4.3367015554	0.6377574758
H	-1.9985511114	3.9275900209	-0.9647400380

H	-3.2283124915	2.8961434438	-0.2018597264
C	-1.7045169973	2.2907926675	2.0814122888
H	-0.9456730583	1.6881066307	2.6022099791
H	-1.8705995838	3.2071054274	2.6711587698
H	-2.6505442344	1.7357817089	2.0688305107
C	0.0000070019	3.5944000954	0.9136531606
H	-0.2916223351	4.4297700319	1.5720471333
H	0.8064409897	3.0264277632	1.4046146448
H	0.4052308619	4.0244096425	-0.0076433379
C	0.4680844276	0.4843552604	-2.7484404357
H	0.0145998090	-0.5108459191	-2.7950252744
H	0.6956329759	0.7966945312	-3.7814546975
H	1.4117201228	0.3925801876	-2.1882340953
C	-1.7446376516	1.6626510793	-2.8898867554
H	-2.2836519928	0.7081413955	-2.9443366108
H	-2.4259224131	2.4132344328	-2.4694372294
H	-1.5179258334	1.9650211750	-3.9258941158
C	0.3388352970	2.8651155367	-2.2317967188
H	-0.2416045738	3.7418807984	-1.9194817427
H	1.2748475389	2.8306442432	-1.6540115193
H	0.6058246373	3.0117429741	-3.2910351319
Pt	1.3516633895	0.3961277880	0.5671785284
C	-1.9337402128	-0.2531925010	-0.0496877180
C	-1.6272832400	-1.3639402981	-1.0753679157
C	-3.4010453319	0.1786539206	-0.2142411754
C	-1.7517825479	-0.8946594398	1.3459345460
H	-1.7497195957	-0.9874389442	-2.0990453433
H	-0.5783568744	-1.6831773962	-0.9612472545
C	-2.5661389443	-2.5609151106	-0.8954558986
H	-3.5662072151	0.6255518793	-1.2056383036
H	-3.6638548362	0.9460958190	0.5283568384
C	-4.3341009055	-1.0270187979	-0.0305791026
H	-0.7013108664	-1.2129972699	1.4482932333
H	-1.9332132972	-0.1648060811	2.1426461760
C	-2.6994565674	-2.0827205348	1.5340448937
H	-2.3170245850	-3.3142702787	-1.6611241273
C	-4.0123383467	-2.0961673733	-1.0765323104
C	-2.3831768053	-3.1585541917	0.4976774176
H	-5.3757838690	-0.6868181537	-0.1551935274
C	-4.1465955972	-1.6130252198	1.3702075833
H	-2.5530133340	-2.4871503726	2.5494350688
H	-4.7042218516	-2.9490938014	-0.9722412419
H	-4.1565128831	-1.6889849620	-2.0921866129
H	-1.3464113480	-3.5127301046	0.6224318641
H	-3.0451426284	-4.0312133283	0.6310702194
H	-4.3887063116	-0.8547164139	2.1346784430
H	-4.8417119867	-2.4558380604	1.5241862897
C	2.7800986042	-2.0483624167	-0.2212085173
C	3.7179256640	-1.5959628824	-1.1220663319
C	4.4408251512	-0.4166518397	-0.8368525612
C	4.2384691092	0.2633790011	0.3473135670
C	3.3026124759	-0.2141782719	1.3100282508

C	2.5228992498	-1.3716304252	1.0076786291
H	3.3465012365	0.1615864086	2.3361114874
H	1.9839397771	-1.9082177009	1.7922426858
H	5.1695487301	-0.0500761367	-1.5628653603
H	4.8234186677	1.1571435386	0.5751497473
H	3.8868451936	-2.1435338710	-2.0504415583
Cl	1.8694475926	-3.4904943468	-0.5620583033

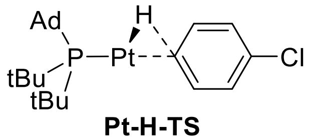

Pt-H-TS

65
Pt-H-TS

Pt	0.9493503452	-0.1796403380	0.2405791997
Cl	7.3560687253	1.0043616925	-0.3050816482
P	-1.2294052855	-0.9330656162	-0.1102787233
C	-1.2506644127	-1.7145965726	-1.8589211048
C	-1.6970079330	-2.2657579100	1.1798346598
C	-2.4253682962	-2.6470153904	-2.1523695507
C	-1.2205074914	-0.6040815398	-2.9150386434
C	0.0770747864	-2.4716206970	-2.0277125789
C	-1.2265509386	-1.7755472641	2.5586130558
C	-3.1815374717	-2.6301202799	1.2352222159
C	-0.8775737834	-3.5282745364	0.8923319520
C	-2.4784897127	0.5008385718	-0.0367794632
C	-2.7152196386	0.8875572657	1.4374099488
C	-3.8423143690	0.2284634377	-0.6939643464
C	-1.8256051711	1.7315546719	-0.7086407322
C	-3.6210519908	2.1185318398	1.5475978526
C	-4.7422225052	1.4668264018	-0.5827482469
C	-2.7382415793	2.9573098760	-0.6142017917
C	-4.9682258932	1.8109275816	0.8903055847
C	-2.9686013200	3.3117051427	0.8529083476
C	-4.0776047031	2.6506628394	-1.2885212749
C	2.8978926271	0.3865957664	0.3501392490
C	3.8488311187	-0.4164170454	1.0075567304
C	3.3773492042	1.4303398813	-0.4638956426
C	5.2150517833	-0.2411806117	0.8028267877
C	4.7400316670	1.6184379503	-0.6789011531
C	5.6497460551	0.7746396771	-0.0454696345
H	-2.3489729224	-3.0050711845	-3.1926408269
H	-2.4304564179	-3.5342131967	-1.5065890761
H	-3.3960453932	-2.1444371797	-2.0477182955
H	-0.3937328019	0.0990645531	-2.7362572930
H	-1.0542584081	-1.0702363906	-3.8998151994
H	-2.1580946860	-0.0387368338	-2.9775852142
H	0.1523624934	-2.8302009547	-3.0677673928
H	0.9391377948	-1.8064672433	-1.8419171344
H	0.1779135323	-3.3397475480	-1.3692839717
H	-1.8041495834	-0.9286575524	2.9420137982

H	-1.3319001432	-2.6034115424	3.2791176739
H	-0.1694203853	-1.4718091048	2.5207028349
H	-3.8097020084	-1.7859493287	1.5489805781
H	-3.5638193194	-2.9994265578	0.2747532207
H	-3.3252945045	-3.4328079531	1.9775407743
H	-1.1895923457	-4.0480277285	-0.0217627460
H	0.1964193313	-3.2979087546	0.8249062821
H	-1.0161504448	-4.2306819227	1.7300299071
H	-3.1957242925	0.0624602743	1.9797189159
H	-1.7442167027	1.0816915218	1.9217645235
H	-4.3374130451	-0.6309713327	-0.2176038091
H	-3.7166403903	-0.0254015152	-1.7562143633
H	-0.8563500446	1.9250132975	-0.2198868853
H	-1.6060580103	1.5349873607	-1.7637564363
H	-3.7713776034	2.3437986806	2.6164353184
H	-5.7085815154	1.2420991114	-1.0641095399
H	-2.2461858139	3.7986625141	-1.1294174256
H	-5.6432981948	2.6787084395	0.9791981590
H	-5.4601047847	0.9681152712	1.4056739494
H	-2.0092974697	3.5559916493	1.3384647202
H	-3.6119446965	4.2037988211	0.9399301376
H	-3.9208489334	2.4169913108	-2.3555772507
H	-4.7373016079	3.5339020738	-1.2485226236
H	2.6661973420	2.1006033315	-0.9524100644
H	5.1008871883	2.4174561031	-1.3290321096
H	1.6307213903	0.7952501019	1.2668090847
H	3.5119526180	-1.2061635959	1.6830577508
H	5.9441254546	-0.8830513374	1.3005732836

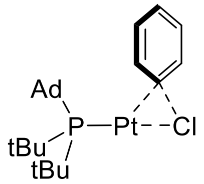

Pt-Cl-TS

65
Pt-Cl-TS

Pt	1.4559018177	-0.6972117829	-0.3796869714
Cl	3.7355762610	-0.0602403595	-0.8024464983
P	-0.6286766047	-1.1835358335	0.1627546873
C	-1.3112703649	-2.6099928416	-0.9304555314
C	-0.6958995697	-1.7433358175	1.9989211608
C	-2.5734970428	-3.2941390162	-0.4024238504
C	-1.5839734126	-2.0652888236	-2.3366875581
C	-0.1992753487	-3.6553368926	-1.1041115769
C	0.2571044981	-0.8492039988	2.8062277434
C	-2.0853387620	-1.7312826624	2.6407199874
C	-0.1121912077	-3.1562191502	2.1096171941
C	-1.7529654827	0.3474991154	-0.0518847759
C	-1.4498994021	1.3521704065	1.0790934075
C	-3.2643312679	0.0607967537	-0.0653328200

C	-1.3682928708	1.0562142799	-1.3707240496
C	-2.2530660478	2.6453996587	0.9026742691
C	-4.0572658787	1.3622330262	-0.2429830638
C	-2.1784726685	2.3435256290	-1.5593494127
C	-3.7472302847	2.3180491899	0.9102750731
C	-1.8798932408	3.3130228099	-0.4182487056
C	-3.6726129698	2.0144662326	-1.5724836416
C	1.8519560708	3.4969249169	-0.3985087920
C	1.8622414570	3.7054513280	0.9818326073
C	2.3665338974	2.7114038946	1.8240037651
C	2.8465487345	1.5138942843	1.3056937836
C	2.7530447899	1.2926901539	-0.0762269833
C	2.3111394044	2.3021164699	-0.9432230331
H	-2.8968628386	-4.0555697263	-1.1318372968
H	-2.4016288093	-3.8154191341	0.5480226610
H	-3.4110590930	-2.5990706948	-0.2622178645
H	-0.7064739553	-1.5308904038	-2.7306192100
H	-1.7880279750	-2.9171703784	-3.0056850055
H	-2.4549377530	-1.3998359260	-2.3811375328
H	-0.5394055975	-4.4087378949	-1.8343226577
H	0.7189686380	-3.1815979266	-1.4837341775
H	0.0546462250	-4.1807228804	-0.1781804763
H	-0.0224485557	0.2091848666	2.8114111332
H	0.2694374866	-1.2029620964	3.8510501761
H	1.2741052475	-0.9191928000	2.3939065825
H	-2.5027191088	-0.7198875882	2.7273981935
H	-2.8090357479	-2.3517939708	2.0974271390
H	-2.0076668130	-2.1367593545	3.6634437292
H	-0.7644835640	-3.9294259765	1.6854919127
H	0.8738483801	-3.2154526167	1.6254990912
H	0.0212785154	-3.3928513278	3.1779097949
H	-1.7094446340	0.9241428714	2.0557762988
H	-0.3697191578	1.5720983136	1.0906228550
H	-3.5776281942	-0.4350729506	0.8644679209
H	-3.5174792875	-0.6196817055	-0.8904751984
H	-0.2887594414	1.2728201078	-1.3477295559
H	-1.5359669595	0.4016516898	-2.2331568243
H	-2.0131318932	3.3191215287	1.7422156443
H	-5.1331433170	1.1195222685	-0.2455515356
H	-1.8892338758	2.7982850290	-2.5215829317
H	-4.3417026573	3.2419615177	0.8097800023
H	-4.0319445549	1.8559979419	1.8713413525
H	-0.8109770672	3.5748130383	-0.4143685833
H	-2.4484482596	4.2500402017	-0.5485602985
H	-3.9054069435	1.3328343493	-2.4086150217
H	-4.2649017700	2.9316516126	-1.7319339548
H	2.3990878670	2.8725561388	2.9042587494
H	3.2595354435	0.7397348414	1.9527297635
H	1.4980541522	4.6466038225	1.3983686479
H	1.4876028270	4.2792142935	-1.0687397415
H	2.3108429084	2.1300368368	-2.0199868817

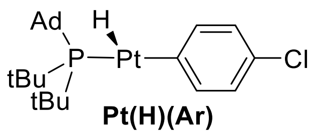

Pt(H)(Ar)

65

Pt(H)(Ar)

P	-1.2683532894	-0.8674242644	-0.0118900930
C	-1.2730317593	-1.6730310378	-1.7503878513
C	-1.7243213980	-2.1872344105	1.2914983577
C	-2.3973193667	-2.6660555257	-2.0361970107
H	-2.2884774179	-3.0531264707	-3.0632772391
H	-2.3845369616	-3.5307396320	-1.3616051638
H	-3.3873390980	-2.1952019608	-1.9656530281
C	-1.2868111719	-0.5946478372	-2.8400492100
H	-0.4849688742	0.1438061172	-2.6940830001
H	-1.1116367702	-1.0858788671	-3.8112326591
H	-2.2444825206	-0.0654746322	-2.9131732101
C	0.0995523918	-2.3575158558	-1.8748806662
H	0.2235428533	-2.7507272401	-2.8977120015
H	0.9293232054	-1.6290092500	-1.7371447379
H	0.2545292135	-3.1849559523	-1.1761783947
C	-1.2537701393	-1.6712037010	2.6610981132
H	-1.7976118517	-0.7871627849	3.0077929149
H	-1.4064382687	-2.4688545473	3.4066769141
H	-0.1829894286	-1.4216593377	2.6349156057
C	-3.2069359495	-2.5578155398	1.3524560804
H	-3.8370281076	-1.7135957378	1.6622536804
H	-3.5887874150	-2.9322979463	0.3935585247
H	-3.3477059603	-3.3571420092	2.0989222197
C	-0.8957770516	-3.4502704509	1.0305383524
H	-1.1893921664	-3.9866074716	0.1199286359
H	0.1781467205	-3.2162878158	0.9779920304
H	-1.0427887793	-4.1417758341	1.8758376099
C	-2.5229703007	0.5559594154	0.0401168274
C	-2.7621928370	0.9777475523	1.5044128234
C	-3.8876078102	0.2601609436	-0.6075740808
C	-1.8762888800	1.7717121357	-0.6649742592
H	-3.2390777483	0.1638209250	2.0669705739
H	-1.7954929711	1.1915829929	1.9888809144
C	-3.6730030309	2.2075413006	1.5833964872
H	-4.3782588429	-0.5873946198	-0.1062303937
H	-3.7619687611	-0.0246037195	-1.6619854311
C	-4.7920906608	1.4973227473	-0.5293154379
H	-0.9060546854	1.9868737921	-0.1898989069
H	-1.6611459651	1.5480841214	-1.7153918777
C	-2.7941900310	2.9955772345	-0.5997798790
H	-3.8239582604	2.4593153549	2.6461344596
C	-5.0190215579	1.8780970585	0.9346545759
C	-3.0252130216	3.3852177349	0.8583309969
H	-5.7576828239	1.2566590915	-1.0043319363
C	-4.1322965100	2.6652487215	-1.2653721735
H	-2.3064120359	3.8259980648	-1.1360952376

H	-5.6968041368	2.7456058898	1.0017613394
H	-5.5080796666	1.0472636427	1.4716532627
H	-2.0668195959	3.6461419479	1.3371342294
H	-3.6718920981	4.2765600739	0.9236704265
H	-3.9748162060	2.4052502655	-2.3261992802
H	-4.7955010438	3.5464872278	-1.2480478747
Pt	0.9671831585	-0.2361770791	0.1578271139
C	2.9423802211	0.2052436082	0.1330333728
C	3.8849758025	-0.5588676761	0.8488156996
C	3.4554880827	1.2262101879	-0.6913735439
C	5.2578634729	-0.3442906574	0.7335934539
C	4.8237110360	1.4597012569	-0.8223915889
H	2.7672102881	1.8635984707	-1.2540486166
C	5.7161883680	0.6664606965	-0.1067722371
H	5.2009212786	2.2530083249	-1.4707645836
H	0.8616280368	0.7563098660	1.3075167276
H	3.5418087389	-1.3508770627	1.5206899899
Cl	7.4311430021	0.9485886307	-0.2587484473
H	5.9722583947	-0.9514789101	1.2930470944

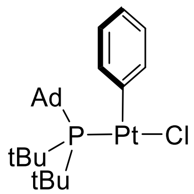

Pt(Cl)(Ph)

65
Pt(Cl)(Ph)

P	-0.1774444224	1.1496359440	-0.1650906253
C	0.0526627371	2.5613469682	1.1135573835
C	-0.1294100425	1.9137860666	-1.9213666992
C	-0.7099254263	3.8536507742	0.8262186453
H	-0.5163161212	4.5696744427	1.6420225465
H	-0.3987203840	4.3375794024	-0.1074238026
H	-1.7949819511	3.6914206082	0.7824970945
C	-0.3290370650	2.0670930152	2.5131287767
H	0.1880502844	1.1299506764	2.7653740654
H	-0.0139317871	2.8295697296	3.2438061524
H	-1.4091977526	1.9239540237	2.6378122829
C	1.5666130249	2.8338017770	1.1686851562
H	1.7740936967	3.5641225490	1.9684231131
H	2.1391299783	1.9256024316	1.4629538164
H	1.9883291776	3.2287899807	0.2407366102
C	0.2000538611	0.8006404107	-2.9236490831
H	-0.5176140793	-0.0256521058	-2.9268384391
H	0.2175166487	1.2368989216	-3.9357319843
H	1.1967621744	0.3901604909	-2.7137170724
C	-1.4169238130	2.6268735582	-2.3387527995
H	-2.2624891572	1.9366192676	-2.4493151504
H	-1.7112216818	3.4183666623	-1.6380033382
H	-1.2550489762	3.0993352212	-3.3214392055

C	1.0418478414	2.8980487176	-2.0247705485
H	0.8951664378	3.8167568338	-1.4452971254
H	1.9885390037	2.4276088806	-1.7207239616
H	1.1461330760	3.1937204220	-3.0809224206
C	-1.8453554180	0.2835827917	0.1129135383
C	-2.1595867823	-0.6152366475	-1.1024727017
C	-3.0310764712	1.2403178139	0.3402413915
C	-1.7217909880	-0.6452876083	1.3409484591
H	-2.2839329538	-0.0073110839	-2.0085896198
H	-1.3247295696	-1.3104522031	-1.2793116608
C	-3.4525058779	-1.4049960619	-0.8779041752
H	-3.1596693436	1.9176235868	-0.5153671905
H	-2.8498749720	1.8699267675	1.2233586352
C	-4.3241406318	0.4406177342	0.5573193945
H	-0.8921516726	-1.3446213792	1.1858662203
H	-1.4931833690	-0.0728863214	2.2458780401
C	-3.0182943929	-1.4292610404	1.5647614302
H	-3.6396366377	-2.0198814316	-1.7735041614
C	-4.6096704215	-0.4271488386	-0.6698926841
C	-3.3003878001	-2.3058317440	0.3460078614
H	-5.1516973804	1.1530166566	0.7110008337
C	-4.1752412559	-0.4548655535	1.7885639296
H	-2.8861504561	-2.0631216314	2.4569627143
H	-5.5556739693	-0.9771616988	-0.5320890818
H	-4.7367589263	0.2077355590	-1.5637852130
H	-2.4720625116	-3.0171100134	0.1915766161
H	-4.2179084665	-2.8983252871	0.5013037091
H	-3.9860350534	0.1600942505	2.6851550178
H	-5.1127112323	-1.0067012422	1.9710744291
Pt	1.7369538495	0.0158676164	0.2706359524
Cl	3.8655474210	-0.6377099622	0.8871652976
C	1.1891219289	-1.8071491236	-0.2231134091
C	0.9568832073	-2.7160275964	0.8194886416
C	1.0913488267	-2.2554349614	-1.5433275769
C	0.5625062957	-4.0242109819	0.5402336472
H	1.0869083965	-2.4019267467	1.8566729082
C	0.6993406305	-3.5681701755	-1.8149167012
H	1.3215564306	-1.5886926188	-2.3722729370
C	0.4166287055	-4.4542155208	-0.7781463390
H	0.3777954077	-4.7147659518	1.3673282678
H	0.6217947321	-3.8971966925	-2.8546433314
H	0.1069724641	-5.4791044443	-0.9949278604
